# Web of venom: exploration of big data resources in animal toxin research

**DOI:** 10.1093/gigascience/giae054

**Published:** 2024-09-09

**Authors:** Giulia Zancolli, Björn Marcus von Reumont, Gregor Anderluh, Figen Caliskan, Maria Luisa Chiusano, Jacob Fröhlich, Evroula Hapeshi, Benjamin-Florian Hempel, Maria P Ikonomopoulou, Florence Jungo, Pascale Marchot, Tarcisio Mendes de Farias, Maria Vittoria Modica, Yehu Moran, Ayse Nalbantsoy, Jan Procházka, Andrea Tarallo, Fiorella Tonello, Rui Vitorino, Mark Lawrence Zammit, Agostinho Antunes

**Affiliations:** Department of Ecology and Evolution, University of Lausanne, 1015 Lausanne, Switzerland; SIB Swiss Institute of Bioinformatics, 1015 Lausanne, Switzerland; Goethe University Frankfurt, Faculty of Biological Sciences, 60438 Frankfurt, Germany; LOEWE Centre for Translational Biodiversity Genomics, 60325 Frankfurt, Germany; Department of Molecular Biology and Nanobiotechnology, National Institute of Chemistry, 1000 Ljubljana, Slovenia; Department of Biology, Faculty of Science, Eskisehir Osmangazi University, 26040 Eskişehir, Turkey; Department of Agricultural Sciences, University Federico II of Naples, 80055 Portici, Naples, Italy; Department of Research Infrastructures for Marine Biological Resources, Stazione Zoologica Anton Dohrn, Villa Comunale, 80121 Naples, Italy; Veterinary Center for Resistance Research (TZR), Freie Universität Berlin, 14163 Berlin, Germany; Department of Health Sciences, School of Life and Health Sciences, University of Nicosia, 1700 Nicosia, Cyprus; Veterinary Center for Resistance Research (TZR), Freie Universität Berlin, 14163 Berlin, Germany; Madrid Institute of Advanced Studies in Food, Precision Nutrition & Aging Program, 28049 Madrid, Spain; SIB Swiss Institute of Bioinformatics, Swiss-Prot Group, 1211 Geneva, Switzerland; Laboratory Architecture et Fonction des Macromolécules Biologiques, Aix-Marseille University, Centre National de la Recherche Scientifique, Faculté des Sciences, Campus Luminy, 13288 Marseille, France; Department of Ecology and Evolution, University of Lausanne, 1015 Lausanne, Switzerland; SIB Swiss Institute of Bioinformatics, 1015 Lausanne, Switzerland; Department of Biology and Evolution of Marine Organisms, Stazione Zoologica Anton Dohrn, 00198 Rome, Italy; Department of Ecology, Evolution and Behavior, Alexander Silberman Institute of Life Sciences, Faculty of Science, The Hebrew University of Jerusalem, 9190401 Jerusalem, Israel; Engineering Faculty, Bioengineering Department, Ege University, 35100 Bornova-Izmir, Turkey; Laboratory of Transgenic Models of Diseases, Institute of Molecular Genetics of the Czech Academy of Sciences, 252 50 Vestec, Czech Republic; Institute of Research on Terrestrial Ecosystems (IRET), National Research Council (CNR), 73100 Lecce, Italy; Neuroscience Institute, National Research Council (CNR), 35131 Padua, Italy; Department of Medical Sciences, iBiMED, University of Aveiro, 3810-193 Aveiro, Portugal; Department of Clinical Pharmacology & Therapeutics, Faculty of Medicine & Surgery, University of Malta, 2090 Msida, Malta; Malta National Poisons Centre, Malta Life Sciences Park, 3000 San Ġwann, Malta; CIIMAR/CIMAR, Interdisciplinary Centre of Marine and Environmental Research, University of Porto, 4450-208 Porto, Portugal; Department of Biology, Faculty of Sciences, University of Porto, 4169-007 Porto, Portugal

**Keywords:** venom resources, toxin databases, machine learning, drug discovery, antivenom, proteomics, peptidomics, transcriptomics, genomics

## Abstract

Research on animal venoms and their components spans multiple disciplines, including biology, biochemistry, bioinformatics, pharmacology, medicine, and more. Manipulating and analyzing the diverse array of data required for venom research can be challenging, and relevant tools and resources are often dispersed across different online platforms, making them less accessible to nonexperts. In this article, we address the multifaceted needs of the scientific community involved in venom and toxin-related research by identifying and discussing web resources, databases, and tools commonly used in this field. We have compiled these resources into a comprehensive table available on the VenomZone website (https://venomzone.expasy.org/10897). Furthermore, we highlight the challenges currently faced by researchers in accessing and using these resources and emphasize the importance of community-driven interdisciplinary approaches. We conclude by underscoring the significance of enhancing standards, promoting interoperability, and encouraging data and method sharing within the venom research community.

## Background

Venomous organisms possess the remarkable ability to synthesize and deliver potent cocktails of bioactive compounds known as venoms, which can elicit profound physiological effects in other organisms. These complex mixtures of proteins, peptides, small organic molecules, and inorganic elements have undergone millions of years of evolution, primarily driven by selective pressure such as predation or defense [1]. Animal venoms have captivated human curiosity for centuries, and recently, technological advancements in diverse research fields, especially in molecular biology, have propelled an increasing interest within the scientific community. This has attracted attention from industry, which recognizes the opportunities presented by animal toxins as drug candidates [2–7], diagnostic tools [8, 9], biopesticides, antimicrobial and antiparasitic agents [10, 11], and biological markers to study human physiology [12, 13].

Modern venom research is thus highly multidisciplinary, and it requires the ability to manipulate and analyze a heterogeneous array of data [14]. The emergence and integration of multiomics technologies such as proteomics, transcriptomics, and, more recently, whole-genome data has revolutionized the characterization of venom components and highlighted their biotechnological potential [14]. Despite the abundance of venom research methods, tools, and resources, their scattered nature limits their comprehensive utilization. Addressing this challenge requires centralized and coordinated web-based resources that could serve as repositories of data and knowledge, facilitating the seamless utilization of analytical tools, bioinformatics pipelines, and related databases, ultimately driving cutting-edge venom research.

In this article, we address the multifaceted requirements of the scientific community by discussing web resources, databases, and tools generally used in venom- and toxin-related research. We compiled them into a comprehensive, interactive table freely available on VenomZone [15]. To gather insights into the most prevalent resources used by both novice and seasoned venom researchers, we carried out a survey targeting the members of the European Venom Network (EUVEN) COST Action CA19144 [16] and the participants of the First International Congress of the EUVEN held virtually in September 2021. While this survey primarily focused on European researchers, limiting its comprehensiveness of the global venom research landscape, it served as a springboard to populate our resource list. More important, it enabled us to identify the key challenges and needs faced by venom scientists. Here, we highlight these challenges and discuss the necessity for user-friendly tools and innovative, community-driven approaches. Furthermore, we emphasize the importance of raising standards, enhancing interoperability, and promoting data and method sharing within the field of venom research. Lastly, we spur the idea to compose, curate, and mine a unified venom-specific database that would report venoms and toxins of diverse animal species, including genome architecture and function, whole proteome composition, toxin targets, mechanism of action, and ecological and evolutionary data.

## Resources in Venom Research: State-of-the-Art

### Overview of main web resources

**Figure 1:**
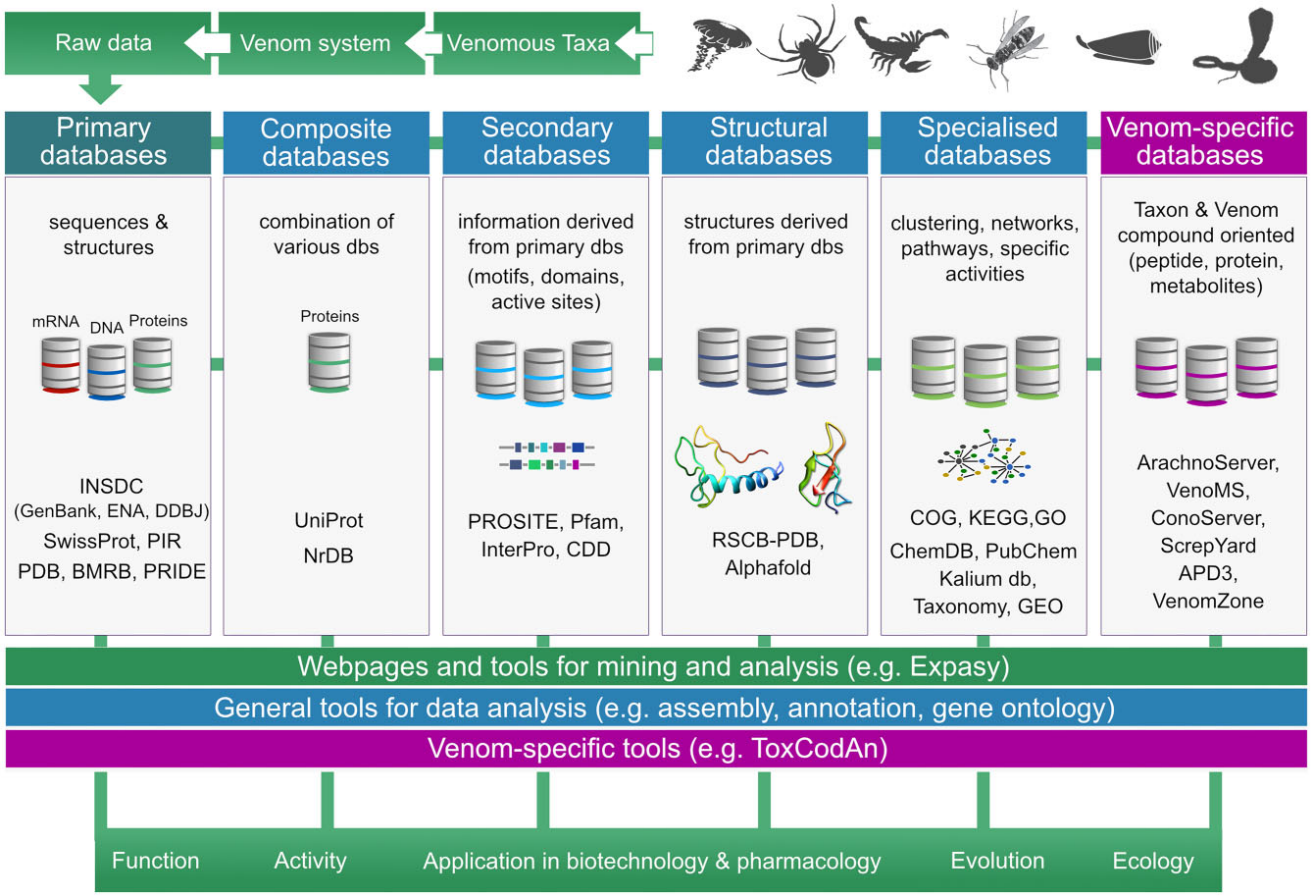
Specialized and generalist web resources, databases, and tools used in venom research. In a typical venom research workflow, raw data from venoms or venom glands are deposited in primary databases, and information is generally subsequently stored in secondary and specialized databases. Such information can be accessed and analyzed using different tools for a variety of research purposes. Dbs = databases.

The cornerstone of virtually any venom research endeavors entails the identification of venom compounds, encompassing their compositional diversity (e.g., protein families), variability (e.g., intra- and interspecies, sex-linked, seasonal, environmental), evolutionary traits, mode of action, and toxicity attributes (e.g., neurotoxicity, hemolytic potency, enzymatic activity, LD50, ED50, clearance rates). This initial step heavily relies on information found in several biological databases (Fig. 1).

The raw data are generally deposited in generalist repositories such as the Proteomics IDEntification (PRIDE) database for mass spectrometry data [17] or the DNA Data Bank of Japan (DDBJ), the European Nucleotide Archive (ENA), and the NCBI GenBank for nucleic acid data. Nucleotide sequences can also be found in venom-specific databases like ArachnoServer [18] and ConoServer [19], which additionally provide protein sequences, classification of gene superfamilies, cysteine frameworks, information on pharmacological activities of toxins, and sequence analysis tools (see following section). Amino acid sequences derived from direct sequencing or from translated nucleotide sequences are mostly available in 2 generalist databases, UniProtKB and NCBI protein. The Tox-Prot annotation project of UniProtKB/Swiss-Prot provides access to venom protein sequences and links to additional web resources [20]. Considering tools, UniProtKB supports BLAST searches (otherwise directly available on the NCBI website), sequence alignment, searches for similar proteins, and links to various features in the Expasy Resource Portal [21]. The species from which the data originate are generally reported in the metadata and linked to taxonomy databases such as NCBI or UniProtKB Taxonomy (Supplementary Information Table S1).

The 3-dimensional (3D) structure of peptides and proteins is important to understand their function and mode of interaction with their molecular targets. The most comprehensive databases holding structural information are the Research Collaboratory for Structural Bioinformatics Protein Data Bank (PDB) [22], the Biological Magnetic Resonance Data Bank (BMRB) [23], and the Electron Microscopy Data Bank (EMDB) [24]. The structures in PDB are primarily determined through X-ray crystallography or nuclear magnetic resonance (NMR) spectroscopy and increasingly by cryo-electron microscopy (cryo-EM), although the latter is only from molecules or molecular complexes with masses less than 100 kDa. BMRB is a database of NMR spectroscopic data from peptides, proteins, nucleic acids, and other biologically relevant molecules, while EMDB archives 3D maps of biological specimens from transmission electron microscopy experiments. Cryo-EM holds great potential for investigating toxin-receptor binding [25, 26]. Visualization of toxin 3D structures is provided in ArachnoServer and ConoServer, as well as in UniProtKB. Additionally, the AlphaFold Protein Structure Database [27] provides access to over 200 million 3D structures predicted by AlphaFold, an artificial intelligence (AI) system developed by Google DeepMind based on a neural network model [28].

A wide array of specialized databases for researchers interested in exploring biological pathways (e.g., the KEGG [29]), gene function classification (e.g., the Gene Ontology [GO] Resource [30]), or more specific information on compounds (e.g., PubChem [31], KaliumDB [32], ScrepYard, KNOTTIN [33, 34]) is discussed in the sections below and listed in Supplementary Information Table S1.

Currently, information on venoms and toxins is dispersed across a multitude of resources, both generalists and specialists, each offering varying types of data and occasionally resulting in redundancy. This scenario presents both advantages and disadvantages. On one hand, the proliferation of openly accessible data represents a goldmine for basic as well as applied research. Conversely, differences in data formats and content between disparate sources make it challenging to aggregate information and sometimes result in inconsistencies. For instance, the annotation related to the mature and precursor sequence of a toxin might differ between a generalist database like UniProtKB, which provides the amino acid sequence of a whole gene, and a venom-specialist database like Arachnoserver or ConoServer, which is instead focused on reporting the active, mature sequence [35].

An additional inconvenience in the database landscape is that some have become obsolete (e.g., SCORPION2 [36]), while others offer limited utility (e.g., ATDB [37] primarily available in Chinese) or are at times unavailable (e.g., ArachnoServer), highlighting the need to constantly curate the available databases [35]. Nonetheless, enduring venom-specific databases and resources include ConoServer, VenoMS [38], T3DB [39], or Tox-Prot. Furthermore, VenomZone [40] is a free web resource that provides information on venoms from 6 major venomous taxa (i.e., snakes, scorpions, spiders, cone snails, sea anemones, and insects), as well as on their molecular targets. Information is structured and accessible through pages on taxonomy (∼170 pages), activity (∼50 pages), and venom protein families (∼40 pages). Each page also provides links to the corresponding proteins in Tox-Prot, classified by species or protein family. Importantly, VenomZone is consulted by around 2,000 visitors every month (average from January to May 2024) and has been regularly updated since its creation in 2015.

Many of the aforementioned websites include some tools for predicting mature peptide boundaries, pharmacological activity, theoretical molecular mass, and so on, while generalist web-based portals (e.g., Expasy [21] and Galaxy [41]) provide comprehensive resources for the analysis of gene expression data, structural biology, text mining, machine learning, and more.

### Resources in genomics

Genomics is increasingly playing a central role in venom research. The advancements and decreasing costs of sequencing technologies have facilitated the availability of genome data from venomous species; consequently, genomics has become indispensable for elucidating the complexity of venom-related genes. Indeed, genomic information is crucial to assess whether divergence in venom composition among species or populations arises from variation in gene copy number, nucleotide sequence, or regulation of gene expression [42–45].

One major advantage of genome data is that it eliminates artifacts from *de novo* proteo-transcriptomics, providing highly accurate results for predicting venom genes and identifying gene and protein variants, including all related transcript and protein-based modifications [46]. To achieve this, transcriptomic data can be assembled using a genome-guided transcriptome assembly approach (e.g., Trinity assembler [47]). Typically, the preferred method for creating genomes is to map transcripts against the genome sequences (scaffolds) with aligners such as BOWTIE2 [48] and splice-aware tools like HISAT2 [49], STAR, [50] and Tophat2 [51, 52] (although no longer supported). High-quality or reference genomes are generally annotated using transcriptomes from multiple tissue samples, comprehensively identifying most gene variants, which is especially relevant to properly characterize multigene families like many venom proteins [46].

Generating genomes involves using a plethora of tools and software, primarily command line based due to the specificity, computational demands, and challenges associated with genome analysis [53, 54]. Several pipelines have been developed by genome consortia, and the recently developed automated pipeline in Galaxy is expected to revolutionize the pace of reference genome production and annotation [55].

Resources and tools related to genomic data are currently not widely available in a venom-related context. However, there are several web resources for accessing genomes, with NCBI Genome being the primary platform that provides genomic data in conjunction with their respective publications. While NCBI offers a comprehensive collection of genomes, some thematic databases, such as Ensembl Metazoa, focus specifically on metazoan reference genomes and offer more tailored data and information [56]. Additionally, Ensembl provides cross-genome resources, annotations, syntenies, and other features, with the added benefit of being more accessible than NCBI through a server and application program interface (API) service. Many genome sequencing consortia, such as G10K, GIGA, i5K, B10K, VGP, EBP, DToL, T2T, and ERGA, provide prepublication information on their planned genomes through dedicated websites, often including unpublished data [57–63]. For example, GenomeArk houses hundreds of high-quality reference genomes and assembly data.

Arguably, the venomous organisms benefiting from the richest genomic resources are Cnidaria (sea anemones, corals, hydroids, and jellyfish). The original reason for the construction of these datasets was the use of several cnidarian species as models for evolutionary developmental biology (“evo-devo”) [64–67] and the specific importance of reef-building corals for marine ecology [68–70]. The availability of these chromosome-scale assemblies, along with rich datasets on small RNA sequencing [71, 72], Chromatin immunoprecipitation sequencing of histone modification marks, and transcriptional regulator proteins [73, 74] for several key species, makes them an excellent resource for studying venom regulatory genomics and evolution. Some of these data can be easily accessed through the SIMRbase genome portal of the Stowers Institute for Medical Research and the *Hydra* 2.0 Genome Project Portal of the National Institutes of Health (NIH).

Despite these advancements, challenges persist in annotating and analyzing toxin-coding genes, as many venom components are part of large, multigene families, and gene comparison tools typically perform better for single-copy genes. Recent studies have demonstrated that analyzing the genome structure and arrangements of genes and their flanking regions across multiple species, known as micro-synteny, is the most effective method for unambiguously unraveling the origin and evolution of many understudied multigene venom protein families or short toxin genes [46, 75–77]. Another challenge is that many venom gene families are poorly studied and functionally characterized, with misleading naming conventions often implying phylogenetic relationships based on similar allergenic responses in bioactivity tests (e.g., venom allergens). Therefore, availability of a dedicated database based on phylogenetic relationships rather than naming conventions would be valuable for analyzing venom gene families. An example of a similar database is PhylomeDB, a catalog of gene phylogenies (phylomes) with multisequence alignments, phylogenetic trees, and ortholog predictions [78]. A promising specialized new resource is ToxCodAn-Genome, an automated pipeline for annotating toxin genes in genomes [79]. While it relies on prior knowledge of venom genes, and it has been tested on a set of well-known venomous lineages, it still overlooks rare venomous taxa and more species-specific gene families.

A branch of biology that is increasingly being explored for insights into venom production and phenotype changes is epigenetics [42, 80], the study of heritable traits occurring without DNA change (e.g., DNA methylation, histone modifications, chromatin architecture, noncoding RNA). Such changes are not erased by cell division, regulating gene expression, and altering cellular/physiological phenotypic traits influenced by environmental factors. A popular web-based genomic data exploration tool that provides visualization, integration, and analysis of epigenomic datasets is the WashU Epigenome Browser [81]. This browser enables the interaction of 1D (genomic features), 2D (Hi-C data), 3D (chromatin structure), and 4D (gene/genomic regions as a function of time) data assessment, serving and expanding the data hubs from large consortia such as 4DN, Roadmap Epigenomics, TaRGET, and ENCODE. However, it currently does not include any venomous taxa.

### Resources in transcriptomics

RNA sequencing is one of the most widely employed strategies used to characterize venom components by sequencing mRNA from dissected venom glands. This technique enables the acquisition of complete precursor sequences, which can then be used to build a custom database for mass spectrometry (MS)–based searches of crude venom (proteo-transcriptomics). Although genomes from venomous organisms are now becoming available, *de novo* transcriptome assembly (often coupled with subsequent proteome analysis) remains the most common method to describe venom compositions and for identifying novel toxin isoforms.

Due to the high computational demands of this process, most transcriptomics analyses are conducted on workstation computers, high-performance clusters, or via cloud computing and therefore use command-line tools. The most widely used assembler for venom gland transcriptomes is undoubtedly Trinity [47] and its companion Trinotate pipeline [82], which predicts coding regions and searches for homology against multiple databases. However, as of March 2024, Trinotate is no longer under active development or support. There are also more bioinformatics knowledge-wise demanding multiassembly pipelines that combine different assemblers and cover a larger space of gene models and reconstructed transcripts; 1 example is the Oyster River pipeline [83].

Functional annotation is typically performed manually through BLAST searches of translated amino acid sequences against UniProtKB, NCBI RefSeq, and other relevant databases (Supplementary Information Table S1), along with domain searches using tools like HMMER [84] or InterProScan [85] against Pfam [86], CDD [87], or own custom databases (e.g., [88]). To facilitate the identification of toxins, several predictor tools have been developed specifically for venom components. Some of these pipelines can be run locally from the command line (e.g., Venomix [89], ToxClassifier [90], TOXIFY [91], and DeTox [92]), while others, such as ToxDL [93] and ConoPrec on ConoServer [19], among others (Supplementary Information Table S1), can be run online through web interfaces where the translated amino acid sequences can be directly uploaded. Additionally, transcripts can be functionally annotated with GO terms using online deep learning approaches such as Pannzer2 [94] and filtering for transcripts annotated with terms like “toxin activity” or “modulation of process of another organism.”

While most transcriptomics studies on venomous animals focus on venom glands, comparative transcriptomics, which compares gene expression between venom glands and other tissues, provides further valuable insights. For instance, this approach can help with the annotation of a transcript as a venom protein, since toxin genes are generally uniquely or predominantly expressed in venom glands. Additionally, it helps identify pathways and genes involved in venom component biosynthesis and secretion [95–97]. After transcript quantification using command-line tools like Kallisto [98], differential expression analysis can be performed in R using various packages (e.g., edgeR [99]). The resulting list of venom gland upregulated genes can be subjected to enrichment analysis for GO terms and KEGG pathways, revealing chaperones and other proteins important for protein folding and maturation or those secreted with toxins to facilitate their targeting.

RNA sequencing data, both raw and processed, can be archived in NCBI. Raw reads are deposited directly in the SRA archive or through the ENA either interactively or through the command line, while assemblies can be archived in the Transcriptome Shotgun Assembly (TSA) sequence database, although it does not accept sequences below 200 bp. Unlike the compulsory raw data submission, assemblies are not mandatory in most journals and are therefore often not uploaded or published as supplementary data (e.g., [100–104]). Gene expression quantifications can be uploaded on the NCBI Gene Expression Omnibus (GEO) archive.

Archiving sequencing and gene expression data is crucial and highly recommended for ensuring their accessibility and reproducibility. By making the assemblies and the expression levels of the corresponding transcripts freely available, researchers can prevent the duplication of effort and unnecessary reassembly and mapping of raw reads, allowing others to readily access and use this essential information for their own studies.

### Resources in proteomics and peptidomics

Proteomics analysis plays a crucial role in venom research, as animal venoms are mostly composed of peptides and proteins. MS methods are commonly employed to identify venom components using 2 main approaches: bottom-up and top-down proteomics [14]. In bottom-up proteomics, venom components are enzymatically digested, and the resulting peptides are individually analyzed by tandem MS. Conversely, top-down approaches analyze intact venom proteins without any prior fragmentation, necessitating high-resolution MS instruments. In both approaches, peptides and proteins are identified through database-based or *de novo* searches. A database-based search matches spectra against an existing database, often derived from venom gland *de novo* transcriptome assembly or from other aforementioned datasets, while a *de novo* search infers peptide sequences directly from the mass spectra without relying on prior genomics or transcriptomics data [105]. Advancements in bottom-up proteomics have led to the development of user-friendly tools, democratizing complex data analysis. Similar to genomics and transcriptomics, proteomics analyses on the raw data are mostly performed locally or on a computer cluster, while online resources are applied for downstream analyses.

For bottom-up proteomics, prominent proprietary database search engines like Mascot [106] and PEAKS DB [107] are commonly used for venom protein identification. Additionally, software tools like ProteomeDiscoverer [108] integrate multiple search algorithms such as Sequest [109], Mascot, and Byonic [110] for peptide identification and protein characterization. Freely available platforms, including pFind 3 [111], MSFragger [112], and PeptideShaker [113], offer powerful tools for identifying venom components and characterizing posttranslational modifications (PTMs). Other software solutions like MaxQuant [114] and Skyline [115] enable identification and quantification of venom proteins using data-dependent acquisition (DDA) methods. To overcome the limitation of DDA, platforms such as DIA-NN [116] and MaxDIA [117] use data-independent acquisition (DIA) methods [118]. In contrast to database-based searches, *de novo* sequencing software like Novor [119] and pNovo [120] facilitate fast and accurate peptide sequencing, although it can be challenging for complex spectra and peptides with extensive PTMs.

Top-down approaches aim to characterize entire toxins, including their isoforms and PTMs, and have recently been applied to venom research [121]. In database-based searches, software such as OpenMS [122], MZmine [123], MS-Deconv [124], and Msconvert [125] are commonly used for deconvoluting complex data. Additionally, MS-Align+ [126], MASH Suite [127], pTop [128], and TopMG [129] allow for high-throughput and automated protein sequence matching of multiple isoforms with high confidence. For *de novo* searches, license-based software like PEAKS (Bioinformatics Solutions Inc.) and ProSight PC (Thermo Fisher Scientific) are generally used, as well as free academic licenses for TopPIC [130] and Informed-Proteomics [131].

AI tools are emerging in proteomics to predict protein structures, pharmacological properties, and interaction partners. Toxin-specific web server tools include ToxinPred [132], ToxinPred2 [133], and ToxClassifier [90] (although unavailable as of April 2024), while non-toxin-specific platforms include Peptide Ranker [134] and PEP-FOLD3 [135], which use machine learning algorithms to predict and design peptides from amino acid sequences. The newest version of PEP-FOLD4 [136] accounts for pH conditions and salt concentration conformations, which are critical parameters for accurate structure prediction. Well-known servers based on machine learning approaches include AlphaFold2 [28], available in ColabFold [137], RoseTTAFold [138], and RaptorX [139], which are based on PDB structures, multiple sequence alignments, and specific algorithms to learn the backbone conformations and side chain–side chain contacts. However, limitations exist, particularly with the accuracy of predictions when signal peptides, pro-peptides, or PTM positions are not specified in the input amino acid sequence. Despite challenges, AI tools offer promising capabilities in predicting unknown protein structures.

Raw proteomics data can be deposited in repositories like PRIDE [17] and Mass Spectrometry Interactive Virtual Environment (MassIVE) [140], which play a crucial role in facilitating collaboration and reproducibility. Additionally, MassiVE offers tools for reanalyzing spectral datasets, comparing results, and more.

### Resources in metabolomics

The main objective of metabolomics is to identify and quantify the metabolites that exist in biological fluids, cells, and tissues. Amines, organic acids, steroids, alkaloids, and sugars are considered the substances of the metabolome. To date, the elucidation of metabolite structures is mainly performed by studying the literature and comparing the MS/MS spectra of related metabolites. Comprehensive databases include the Human Metabolome Database (HMDB) [141] and KEGG [29], which offer different qualitative and quantitative data for human metabolites and information about metabolomic pathways. HMDB is currently the database containing the largest data collection of MS/MS fragmentation spectra of metabolites [141, 142]. An interesting tool is offered by the Global Natural Products Social Molecular Networking (GNPS) [143], a web-based mass spectrometry ecosystem that aims to be an open-source and open-access knowledge base for community-wide organization and sharing of raw, processed, or identified tandem mass (MS/MS) spectrometry data. GNPS aids in identification and discovery throughout the entire life cycle of data, from initial data acquisition to postpublication.

The only existing venom-specialist metabolite database is VenoMS [38], which focuses on low molecular mass metabolites from spider venoms. VenoMS gathers known structures of spider venom metabolites and offers a fragment ion calculator (FRIOC) for the prediction of fragment ions for the linear polyamine derivatives. This website can be considered complementary to *ArachnoServer*. Despite its usefulness, this resource is limited to spiders and is not included in the typical automated MS analyses.

A suggestion for a future endeavor could be to extend the content of VenoMS to other venomous organisms and create a more comprehensive online database of venom metabolites. As venom metabolomics is still in its infancy, challenges rely mostly in the chemical identification of metabolites and the integration with data from other omics platforms.

### Resources in translational research

The vast biotechnological and biomedical potential of animal venoms and toxins is undeniable, with well-documented bioactivities ranging from analgesic, immunomodulatory, anticancer, antimicrobial, and antiparasitic properties [2–5, 144]. This potential translates into a growing number of venom-derived drugs, with already 11 approved by the US Food and Drug Administration and European Medicines Agency, and many more in preclinical or clinical development. Beyond medicine, venom toxins hold promise for diagnostics, nanopore-based sensing, agrochemicals, and cosmetics [8, 10–13, 145]. However, despite the evident opportunities, the translation of basic research into concrete applications is a lengthy process that requires the generation of a variety of data and access to a wide array of different tools and databases. In this section, we provide an overview of the available resources pertinent to venom and toxin research from a biomedical and translational perspective.

In a typical workflow for venom component discovery, the first step involves candidate identification. This can be achieved by generating new data by means of genomics or proteo-transcriptomics analysis or by mining existing databases. Typical databases include ArachnoServer, Toxin and Toxin Target Database (T3DB), PubChem, and UniProtKB/Swiss-Prot, among others (Supplementary Information Table S1). T3DB is particularly useful as it combines detailed toxin data with comprehensive receptor information, molecular and biological properties, toxin effects, and potential therapeutic applications [39]. For peptide-based cancer research, CancerPPD4 [146], canSAR [147], ApInAPDB [148], PaccMann [149], and EviCor [150] provide platforms for the exploration of the mechanism of action, function, binding target, affinity, structural information, and other physicochemical features of peptides. Furthermore, they offer AI-based predictions of anticancer compound sensitivity and other properties to inform drug discovery. A comprehensive database useful in translational research was the discontinued VenomKB [151], which included data on venom’s molecular components and their potential applications in drug discovery and development.

The databases can be mined manually to select a list of potential candidates, which can be further screened using the prediction tools mentioned earlier. Alternatively, databases can be used to build machine learning models based on random forest, support vector machine, or artificial neural network algorithms, which can process a vast amount of data and identify patterns to predict potential drug targets. This first crucial step of target identification poses a challenge in venom research as the toxin information is scattered across several databases. Thanks to the advent of the Semantic Web (SW), the tedious process to manually mine different life science databases can be significantly reduced [152]. SW provides a common framework that enables data to be shared and reused across different data sources. Combining and querying these data sources are possible by using a standard semantic query language like SPARQL. A solution to meaningfully access the databases containing animal venom information is to federate them by applying SW technologies that enable semantic queries across them [153]. For instance, currently UniProtKB and PubChem can be jointly queried by writing a single federated SPARQL query [154].

Once potential candidates are characterized, further steps include prediction of molecular targets and interactions with the toxins. Databases such as the mousephenotype.org for mammals [155], zfin.org for zebrafish [156], and flybase.org for insects [157] can be explored for predicting the effects of toxin intervention on a systemic level and specific regulatory functions, as well as identifying promising pharmaceutical or bioinsecticides targets. Web-based prediction tools for molecular docking include SwissDock [158], the more recently developed PPI-Affinity model [159], and the CAMP model [160] to elaborate on target predictions for peptides and proteins. Molecular docking and molecular dynamics simulation models such as quantitative structure–activity relationship (QSAR), quantitative structure–property relationship (QSPR) analysis, pharmacophore modeling, and iBitter-SCM are frequently used to decipher peptide and protein interactions [161].

Once a lead compound has been identified and selected, it can be modified to have unique and desirable properties, for instance, to modulate their target selectively and induce a therapeutic rather than a harmful toxic effect [162]. ToxinPred and ToxinPred2 include tools to design all possible single mutant analogues of a peptide and predict whether they are toxic or not, as well as to optimize the peptide sequence to get maximum, minimum, and desired toxicity. In addition, ToxinPred offers users to calculate various physicochemical properties.

While the approaches delineated above facilitate the search among known venom compounds, enduring challenges remain for the prediction of toxins with undescribed new mechanisms of action and the identification of the potential off-target effects that might limit the usefulness of the molecule as a putative therapeutic drug [163], although current machine learning algorithms present a promising avenue for the discovery of molecules with novel activities. Despite the potential benefits, it is important to acknowledge that the principles of open science may not always be guaranteed in translational and applied research, often due to confidentiality agreements associated with preliminary studies on toxin activity prediction and application.

### Resources in antivenom production and administration

Scientists working in the field of antivenom research are typically interested in a variety of information spanning from the geographic distribution of the venomous species to their venom composition and variation, toxin structure, and bioactivity, which all impact antivenom efficiency. Most of the resources related to this kind of information have been already discussed in previous sections and are listed in Supplementary Information Table S1; therefore, here we focus on the resources available for antivenom producers.

A first important resource is represented by the World Health Organization (WHO) guidelines, which provides comprehensive and important manuals for antivenom manufacturers on the design, production, control, and regulation of high-quality antivenom immunoglobulins. These guidelines are regularly updated to provide framework guidance to national regulatory bodies for securing the products they offer. Technical bulletins, reports, and documents are also available on the WHO website. Within the scope of WHO web resources, in addition to pharmacopoeia requirements, current antidote production, especially the improvement of studies and technologies carried out under GMP quality system conditions, is ensured. Additionally, WHO manages the snakebite information and data platform as part of the 2019–2030 global strategy for the prevention and control of snakebite envenoming, which is within the scope of neglected tropical diseases by WHO. This web source platform is part of a collaboration between the departments for the control of Neglected Tropical Diseases (WHO/NTD) and the Dissemination of Data for Impact and analytics (WHO/DDI). Another data source created for easy access to antivenom in cases of envenoming caused by poisonous animals is the Munich AntiVenom INdex (MAVIN) created by the Munich Poison Center. MAVIN gathers a list of venomous animals, antivenom holding centers, antivenoms, and correlated information.

In addition to international web resources such as WHO and MAVIN, some countries have developed national web resources to help staff at zoos and aquariums managing the supply of antivenom and finding the right antivenom when they need it. For instance, an online Antivenom Index was created in 2006 by the Association of Zoos and Aquariums (AZA) and America’s Poison Centers (previously known as American Association of Poison Control Centers). The University of Arizona College of Pharmacy is currently responsible for maintaining, updating, and hosting this index. However, only representatives of poison control centers and AZA-accredited institutions have access to the Antivenom Index.

### Resources in clinical toxinology

Several freely available resources offer information on venoms and venomous animals, which are relevant to clinical toxicologists and toxinologists. A central resource is the “Clinical Toxinology Resources” website, which provides comprehensive information on venomous and poisonous animals, plants, and mushrooms from around the world (Supplementary Information Table S1). This repository receives support from experts around the world, and it features a searchable database that allows users to find specific organisms by common or scientific names, family, country, or region. Another useful resource is PubChem, which gathers information on chemical structure, chemical and physical properties, biological activity, toxicity, and medical management guidance, among others.

Most clinical toxinology and toxicology databases cater specifically to poison centers and are accessible only to registered health care professionals. Nonetheless, some are reachable upon subscription fees and may offer free or reduced-cost access, particularly for users in low-income countries. For instance, AfriTox offers online and offline versions, primarily for registered health care professionals, with subscription-based access. This database focuses on substances, including venomous exposures, from an African perspective. The Merative Micromedex® POISINDEX® System is widely used worldwide, especially in North America, and provides both summary and in-depth clinical toxicology information, including details on venomous animals, through subscription-based access. Another useful resource is TOXBASE, produced by poison specialists and medical toxicologists, which offers advice on toxin features and exposure management to toxins and venomous animals. While primarily accessible to UK health care professionals, TOXBASE is also used internationally, with special arrangements for certain countries. Lastly, TOXINZ provides information and treatment guidelines, including venomous animal exposures. While primarily designed for use in New Zealand, TOXINZ is accessible in other countries through paid subscriptions.

## Challenges, Needs, and Perspectives of Web Resources in Venom Research

The survey that we conducted within the framework of the EUVEN COST Action [16], although representing only a sample of the worldwide venom research community, provided important insights into the challenges and needs of researchers and clinicians working with animal venoms or toxins. Here, we have summarized and discussed them.

### Challenges

Many scientists in the venom research community expressed disappointment due to the bottleneck caused by the limited expertise in bioinformatics and data management, especially concerning the handling of complex “-omics” pipelines essential for cutting-edge research. Despite the improvements in accessibility offered by databases, there is still a demand for more user-friendly interfaces that seamlessly integrate data and tools into existing pipelines, facilitating the translation of research findings into clinical applications. However, achieving a unified graphical user interface (GUI) software is not easy due to the variety, volume, and complexity of current data, requesting storage on servers alongside the necessary analysis tools. Toxinologists are encouraged to collaborate with bioinformaticians and relevant technology experts in cross-disciplinary projects. Initiatives like EUVEN and organizations such as the Swiss Institute of Bioinformatics provide support and facilitate collaborations by offering access to databases of researchers and their corresponding expertise.

Another challenge faced by venom researchers, particularly those involved in applied aspects like drug discovery, was related to the scattered and diverse nature of information about venoms and toxins across several databases. This issue is not unique to venom researchers but is prevalent among biologists. As the production of biological and health data continues to exponentially grow, so does the number of databases [164]. However, querying is still largely limited to a single database at a time, making it difficult to integrate multiple data types to answer complex biological questions [152]. A step forward in addressing this challenge is the adoption of query languages like SPARQL to search across different databases and perform data manipulation tasks such as exploration, extraction, and annotation. Furthermore, to effectively manage and analyze datasets, standardized terminologies and classification systems are essential. Ontologies and glossaries serve as structured vocabularies that provide a common language for annotating and organizing biological information (e.g., GO, UniProtKB/Swiss-Prot controlled vocabularies). Even though the use of such resources is generally well consolidated along the research pipelines, often different terms are employed to denote the same concept, or conversely, the same term is used to represent multiple concepts across web resources, thereby hindering interoperability [165]. For instance, in Ontobee [166], a catalog and web-based linked data server for semantic terminologies, the term “venom” is described differently in 8 ontologies. This highlights the need for mapping terms between the semantic resources commonly used in the field.

To access the wealth of data, a reliable database needs to be regularly maintained and updated. Its longevity depends on several factors, including the underlying technology and system, the frequency of data updates, its ability to handle growing data volumes, their regular backup, and the database capacity to continue to meet user needs. Ultimately, the decision to maintain a database largely depends on the funding required to support the work of developers and curators, which in turn depends on the size of the database and the number of users.

In terms of data analysis, as venom omics data accumulate, the challenge evolves from basic descriptive comparative findings to the more sophisticated task of integrating multiomics data. This approach ultimately aims to gain a comprehensive understanding of the complexity of biological systems and their underlying mechanisms. To this end, data standardization, advanced computational methods (e.g., machine learning techniques), and interpretation of diverse data types are key to provide meaningful insights. While multiomics integration tools are currently applied in studying complex human diseases [167], they hold great promise for deciphering equally complex venom phenotypes.

### Needs

Despite the abundance of databases containing information on animal toxins, some data remain disorganized and inaccessible due to a lack of structured datasets. For the data to be accessible through query languages, databases need to be machine-readable, meaning they must be formatted in a way that can be processed by software tools. This is also crucial for full implementation of the Findable Accessible Interoperable Reusable (FAIR) principles [168]. The Resource Description Framework (RDF), for instance, is a SW standard data model adopted by many databases for sharing and linking data. Data in RDF can be queried, retrieved, and manipulated using the SPARQL language, which has the advantage that it is graph-based, thus allowing users to join data from multiple, diverse sources (in contrast to SQL, which is a table-based query language). Therefore, there is a need to standardize the structure of databases to run queries on animal venoms and toxin research across them. Furthermore, it is advisable to use existing ontologies and incorporate controlled terms already in use or map redundant terms among them. This can be facilitated by searching existing terms in semantic resource catalogs such as Ontobee, the Ontology Lookup Service (OLS), or BioPortal [152, 169]. This practice prevents unnecessary duplications, reduces redundancy, and enhances data reusability and interoperability, which is particularly relevant to a high multidisciplinary field like venom research.

Another issue raised by the venom research community is the absence of a repository for protocols and methods for recombinantly producing or chemically synthesizing venom peptides, which would benefit researchers by preventing redundant protocol optimization efforts, especially in the case of toxins difficult to refold. Additionally, there is a need for a centralized, nonprofit database of biological materials related to venoms and natural or engineered toxins stored or generated in research institutes, similar to plasmid repositories or even catalogs for museum specimens, to aid researchers in accessing preexisting materials for their own studies.

Ensuring data and information accessibility and standardization to the research and clinician communities and the public remains crucial, as discussed in previous sections. The importance of making these data publicly available is further emphasized by the FAIR principles [159] and the recent European Open Access policies [170], which advocate for open access not only to publications but also to all underlying data. Addressing these needs and challenges will require collaboration and concerted efforts from researchers, clinicians, and organizations to advance venom research and its applications.

### Perspectives on a unified venom web resource

Steps toward satisfying the needs of the venom research community include the creation of a venom-specific resource containing detailed information on venomous species and their venoms and toxins. This database could encompass genome architecture and function of venomous species, venom gland transcriptomes, toxin genes and their translated amino acid sequences, PTMs, 3D structures, pharmacological activities and toxicity levels, molecular and cellular targets, and mechanisms of action, coupled with ecological and evolutionary information of the corresponding species (e.g., diet and geographical distribution). By consolidating such diverse information into a single resource or interface uniting a range of resources, scientists working in the interdisciplinary field of animal venoms and toxins would have a valuable tool at their disposal. It would enable them to access both general and specific information on a vast number of venomous species and toxins and would decrease the time spent on extensive literature searches.

Such a resource could also significantly contribute to venom research by facilitating the classification of venom proteins, aiding in the design of peptides with desired pharmacological properties, and identifying potential interactions. However, the creation and maintenance of such a platform would present considerable challenges, requiring a substantial workforce, financial resources, and international interdisciplinary collaborations to ensure its continual updates and accuracy.

An existing resource like VenomZone could serve as a starting point toward realizing this unified resource. However, significant expansions would be necessary to incorporate the additional data proposed. A promising initiative is the interactive table that we have compiled within the framework of this work and made available on the VenomZone website [15] (Supplementary Information Fig. S4). It includes current web resources relevant to venom research in an interactive way. It therefore represents a positive step toward creating a comprehensive and accessible resource for the entire venom research community.

## Conclusions

- Modern venom research is a multidisciplinary field resulting in the generation and analysis of highly diverse datasets.
- Currently, information on venom and toxin data is scattered across different resources, ranging from generalist to specialized platforms.
- Most multiomics analyses are performed using software and command-line tools that require advanced computational and command-line skills, while most available web resources mainly offer downstream analyses.
- One of the core challenges is accessing and providing information across the different databases. There is an urgent need to establish standards to facilitate interoperability and allow seamless querying of animal venom and toxin research across platforms.
- Progress toward meeting the needs of the venom research community requires the establishment of a dedicated venom-specific resource. VenomZone, together with our newly curated site on demanded tools and resources, represents an important first step towards this goal.

## Supplementary Material

giae054_GIGA-D-24-00165_Original_Submission

giae054_GIGA-D-24-00165_Revision_1

giae054_Response_to_Reviewer_Comments_Original_Submission

giae054_Reviewer_1_Report_Original_Submission Qiong Shi, PhD -- 6/7/2024 Reviewed

giae054_Reviewer_2_Report_Original_Submission Jason Macrander, Ph. D. -- 6/22/2024 Reviewed

giae054_Supplemental_Files

## Data Availability

Not applicable.

## Additional Files

**Additional file 1.pdf: Survey on web resources in venom research**. Questions included in the survey sent to the members of the EUVEN COST Action and the participants of the First International EUVEN Congress in 2021.

**Additional file 2.csv: Answers to the survey**. Anonymized answers to the survey.

**Additional file 3.tsv: Summary of venom research areas**. Contingency table of the research areas represented by the respondents of the survey used to create Supplementary Information Fig. S1.

**Additional file 4.tsv: Summary of organisms studied in venom research**. Contingency table of the organisms studied by the respondents of the survey used to create Supplementary Information Fig. S2.

**Additional file 5.pdf: Supplementary Information**. Overview of the results from the survey and the compiled list of web resources, databases, and online tools utilised in venom research (Supplementary Information Table S1) and available on the VenomZone website.

## Abbreviations

3D: 3-dimensional; AI: artificial intelligence; API: application program interface; AZA: Association of Zoos and Aquariums; BLAST: Basic Local Alignment Search Tool; BMRB: Biological Magnetic Resonance Data Bank; cryo-EM: cryo-electron microscopy; DDA: data-dependent acquisition; DDBJ: DNA Data Bank of Japan; DIA: data-independent acquisition; EMDB: Electron Microscopy Data Bank; ENA: European Nucleotide Archive; EUVEN: European Venom Network; FRIOC: fragment ion calculator; GEO: Gene Expression Omnibus; GNPS: Global Natural Products Social Molecular Networking; GO: Gene Ontology; GUI: graphical user interface; HMDB: Human Metabolome Database; KEGG: Kyoto Encyclopedia of Genes and Genomes; MassIVE: Mass Spectrometry Interactive Virtual Environment; MAVIN: Munich AntiVenom INdex; MS: mass spectrometry; NCBI: National Center for Biotechnology Information; NIH: National Institutes of Health; NMR: nuclear magnetic resonance; PDB: Protein Data Bank; PRIDE: Proteomics IDEntification; PTM: posttranslational modification; QSAR: quantitative structure–activity relationship; QSPR: quantitative structure–property relationship; RDF: Resource Description Framework; SW: Semantic Web; T3DB: Toxin and Toxin Target Database; TSA: Transcriptome Shotgun Assembly; WHO: World Health Organization.
