# Supplementary material for: Web of venom: exploration of big data resources in animal toxin research
Source: Gigascience. 2024 Sep 9;13:giae054. doi: 10.1093/gigascience/giae054 (PMC11382406; doi:10.1093/gigascience/giae054)
Supplement: giae054_GIGA-D-24-00165_Original_Submission [file giae054_giga-d-24-00165_original_submission.pdf]

GigaScience

Web of venom: exploration of big data resources in animal toxin research
--Manuscript Draft--

**Manuscript Number:**	GIGA-D-24-00165
**Full Title:**	Web of venom: exploration of big data resources in animal toxin research
**Article Type:**	Review
**Funding Information:**	European Cooperation in Science and Technology (CA19144) — Dr Maria Vittoria Modica
**Abstract:**	Research on animal venoms and their components spans multiple disciplines including biology, biochemistry, bioinformatics, pharmacology, medicine, and more. Manipulating and analysing the diverse array of data required for venom research can be challenging, and relevant tools and resources are often dispersed across different online platforms, making them less accessible to non-experts. In this paper, we address the multifaceted needs of the scientific community involved in venom and toxin-related research by identifying and discussing web resources, databases, and tools commonly utilised in this field. We have compiled these resources into a comprehensive table available on the VenomZone website (https://venomzone.expasy.org/10897). Furthermore, we highlight the challenges currently faced by researchers in accessing and utilising these resources and emphasise the importance of community-driven interdisciplinary approaches. We conclude by underscoring the significance of enhancing standards, promoting interoperability, and encouraging data and method sharing within the venom research community.
**Corresponding Author:**	Giulia Zancolli, PhD<br>University of Lausanne: Universite de Lausanne<br>Lausanne, SWITZERLAND
**Corresponding Author Secondary Information:**	
**Corresponding Author's Institution:**	University of Lausanne: Universite de Lausanne
**Corresponding Author's Secondary Institution:**	
**First Author:**	Giulia Zancolli, PhD
**First Author Secondary Information:**	
**Order of Authors:**	Giulia Zancolli, PhD
	Björn Marcus von Reumont
	Gregor Anderluh
	Figen Caliskan
	Maria Luisa Chiusano
	Jakob Fröhlich
	Evroula Hapeshi
	Benjamin-Florian Hempel
	Maria P Ikonomopoulou
	Florence Jungo
	Pascale Marchot
	Tarcisio Mendes de Farias
	Maria Vittoria Modica

Powered by Editorial Manager® and ProduXion Manager® from Aries Systems Corporation

	Yehu Moran
	Ayse Nalbantsoy
	Jan Procházka
	Andrea Tarallo
	Fiorella Tonello
	Rui Vitorino
	Mark Zammit
	Agonstinho Antunes
**Order of Authors Secondary Information:**	
**Additional Information:**	
**Question**	**Response**
Are you submitting this manuscript to a special series or article collection?	No
**Experimental design and statistics**<br><br>Full details of the experimental design and statistical methods used should be given in the Methods section, as detailed in our Minimum Standards Reporting Checklist. Information essential to interpreting the data presented should be made available in the figure legends.<br><br>Have you included all the information requested in your manuscript?	Yes
**Resources**<br><br>A description of all resources used, including antibodies, cell lines, animals and software tools, with enough information to allow them to be uniquely identified, should be included in the Methods section. Authors are strongly encouraged to cite Research Resource Identifiers (RRIDs) for antibodies, model organisms and tools, where possible.<br><br>Have you included the information requested as detailed in our Minimum Standards Reporting Checklist?	Yes
**Availability of data and materials**	Yes

All datasets and code on which the conclusions of the paper rely must be either included in your submission or deposited in publicly available repositories (where available and ethically appropriate), referencing such data using a unique identifier in the references and in the "Availability of Data and Materials" section of your manuscript.<br><br>Have you have met the above requirement as detailed in our Minimum Standards Reporting Checklist?	

Manuscript

Click here to access/download;Manuscript;Zancolli_et_al_main_submission.

Web of venom: exploration of big data resources in animal toxin research

Giulia Zancolli[1,2,*], Björn Marcus von Reumont[3,4,*], Gregor Anderluh[5], Figen Caliskan[6], Maria Luisa Chiusano[7,8], Jacob Fröhlich[9], Evroula Hapeshi[10], Benjamin-Florian Hempel[9], Maria P. Ikonomopoulou[11], Florence Jungo[12], Pascale Marchot[13], Tarcisio Mendes de Farias[2,1], Maria Vittoria Modica[14], Yehu Moran[15], Ayse Nalbantsoy[16], Jan Procházka[17], Andrea Tarallo[18], Fiorella Tonello[19], Rui Vitorino[20], Mark Lawrence Zammit[21,22], Agostinho Antunes[23,24]

*First co-authors

Corresponding authors: Giulia Zancolli: giulia.zancolli@gmail.com, Agostinho Antunes: aantunes@ciimar.up.pt

[1]Department of Ecology and Evolution, University of Lausanne, 1015 Lausanne, Switzerland. giulia.zancolli@gmail.com

[2]SIB Swiss Institute of Bioinformatics, 1015 Lausanne, Switzerland. tarcisio.mendes@sib.swiss

[3]Goethe University Frankfurt, Faculty of Biological Sciences, Max-von-Laue-Str. 13, 60438 Frankfurt, Germany. bmvr@arcor.de

[4]LOEWE Centre for Translational Biodiversity Genomics, Senckenberganlage 25, 60325 Frankfurt, Germany

[5]Department of Molecular Biology and Nanobiotechnology, National Institute of Chemistry, Hajdrihova 19, 1000 Ljubljana, Slovenia. gregor.anderluh@ki.si

[6]Department of Biology, Faculty of Science, Eskisehir Osmangazi University, 26040 Eskişehir, Turkey. fcalis@ogu.edu.tr

[7]Department of Agricultural Sciences, University Federico II of Naples, 80055 Portici, Naples, Italy. chiusano@unina.it

[8]Department of Research Infrastructures for Marine Biological Resources, Stazione Zoologica Anton Dohrn, Villa Comunale, 80121 Naples, Italy

[9]Veterinary Center for Resistance Research (TZR), Freie Universität Berlin, 14163 Berlin, Germany. jacobfroehlich@web.de; benjamin.hempel@fu-berlin.de

[10]Department of Health Sciences, School of Life and Health Sciences, University of Nicosia, 46 Makedonitissas Avenue, 1700, Nicosia, Cyprus. hapeshis.e@unic.ac.cy

[11]Madrid Institute of Advanced Studies in Food, Precision Nutrition & Aging Program, 28049 Madrid, Spain. maria.ikonomopoulou@alimentacion.imdea.org

[12]SIB Swiss Institute of Bioinformatics, Swiss-Prot Group, 1 rue Michel Servet, 1211 Geneva, Switzerland. florence.jungo@sib.swiss

[13]Laboratory Architecture et Fonction des Macromolécules Biologiques, Aix-Marseille University, Centre National de la Recherche Scientifique, Faculté des Sciences, Campus Luminy, 13288 Marseille, France. pascale.marchot@univ-amu.fr

[14]Department of Biology and Evolution of Marine Organisms, Stazione Zoologica Anton Dohrn, Via Po 25c, 00198 Rome, Italy. mariavittoria.modica@szn.it

[15]Department of Ecology, Evolution and Behavior, Alexander Silberman Institute of Life Sciences, Faculty of Science, The Hebrew University of Jerusalem, 9190401 Jerusalem, Israel. yehu.moran@mail.huji.ac.il

[16]Ege University, Engineering Faculty, Bioengineering Department, 35100 Bornova-Izmir, Turkey. analbantsoy@gmail.com

[17]Laboratory of Transgenic Models of Diseases, Institute of Molecular Genetics of the Czech Academy of Sciences, Prumyslova 595, 252 50 Vestec, Czech Republic. jan.prochazka@img.cas.cz

[18]Institute of Research on Terrestrial Ecosystems (IRET), National Research Council (CNR), SP Lecce-Monteroni, 73100 Lecce, Italy. andrea.tarallo@cnr.it

[19]Neuroscience Institute, National Research Council (CNR), Viale G. Colombo 3, 35131 Padua, Italy. fiorella.tonello@cnr.it

[20]Department of Medical Sciences, iBiMED, University of Aveiro, 3810-193 Aveiro, Portugal. rvitorino@ua.pt

[21]Department of Clinical Pharmacology & Therapeutics, Faculty of Medicine & Surgery, University of Malta, 2090 Msida, Malta. mark.zammit@um.edu.mt

[22]Malta National Poisons Centre, Malta Life Sciences Park, San Ġwann, Malta

[23]CIIMAR/CIMAR, Interdisciplinary Centre of Marine and Environmental Research, University of Porto, Terminal de Cruzeiros do Porto de Leixões, Av. General Norton de Matos, s/n, 4450-208 Porto, Portugal. aantunes@ciimar.up.pt

[24]Department of Biology, Faculty of Sciences, University of Porto, Rua do Campo Alegre, 4169-007, Porto, Portugal.

Abstract

Research on animal venoms and their components spans multiple disciplines including biology, biochemistry, bioinformatics, pharmacology, medicine, and more. Manipulating and analysing the diverse array of data required for venom research can be challenging, and relevant tools and resources are often dispersed across different online platforms, making them less accessible to non-experts. In this paper, we address the multifaceted needs of the scientific community involved in venom and toxin-related research by identifying and discussing web resources, databases, and tools commonly utilised in this field. We have compiled these resources into a comprehensive table available on the VenomZone website (https://venomzone.expasy.org/10897). Furthermore, we highlight the challenges currently faced by researchers in accessing and utilising these resources and emphasise the importance of community-driven interdisciplinary approaches. We conclude by underscoring the significance of enhancing standards, promoting interoperability, and encouraging data and method sharing within the venom research community.

Keywords

Venom resources, toxin databases, machine learning, drug discovery, antivenom, proteomics, peptidomics, transcriptomics, genomics.

Background

Venomous organisms possess the remarkable ability to synthesise and deliver potent cocktails of bioactive compounds known as venoms, which can elicit profound physiological effects in other organisms. These complex mixtures of proteins, peptides, small organic molecules, and inorganic elements have undergone millions of years of evolution primarily driven by selective pressure such as predation or defence [1]. Animal venoms have captivated human curiosity for centuries, and recently, technological advancements in diverse research

fields, especially in molecular biology, have propelled an increasing interest within the scientific community. This has attracted attention from industry which recognises the opportunities presented by animal toxins as drug candidates [2–7], diagnostic tools [8,9], biopesticides, antimicrobial and antiparasitic agents [10,11], as well as biological markers to study human physiology [12,13].

Modern venom research is thus highly multidisciplinary, and it requires the ability to manipulate and analyse a heterogeneous array of data [14]. The emergence and integration of multi-omics technologies such as proteomics, transcriptomics, and, more recently, whole-genome data has revolutionised the characterisation of venom components and highlighted their biotechnological potential [14]. Despite the abundance of venom research methods, tools and resources, their scattered nature limits their comprehensive utilisation. Addressing this challenge requires centralised and coordinated web-based resources that could serve as repositories of data and knowledge, facilitating the seamless utilisation of analytical tools, bioinformatics pipelines, and related databases, ultimately driving cutting-edge venom research.

In this paper, we address the multifaceted requirements of the scientific community by discussing web resources, databases, and tools generally utilised in venom and toxin-related research. We compiled them into a comprehensive, interactive table freely available on VenomZone (https://venomzone.expasy.org/10897). To gather insights into the most prevalent resources used by both novice and seasoned venom researchers, we carried out a survey targeting the members of the European Venom Network (EUVEN) COST Action CA19144 [15], and the participants of the 1° International Congress of the EUVEN held virtually in September 2021. While this survey primarily focused on European researchers, limiting its comprehensiveness of the global venom research landscape, it served as a springboard to populate our resource list. More importantly, it enabled us to identify the key challenges and needs faced by venom scientists. Here, we highlight these challenges and discuss the necessity for user-friendly tools and innovative, community-driven approaches. Furthermore, we emphasise the importance of raising standards, enhancing interoperability,

and promoting data and method sharing within the field of venom research. Lastly, we spur the idea to compose, curate and mine a unified venom-specific database that would report venoms and toxins of diverse animal species including genome architecture and function, whole proteome composition, toxin targets, mechanism of action, ecological and evolutionary data.

Main text

1. Resources in venom research: state-of-the-art

1.1 Overview of main web resources

The cornerstone of virtually any venom research endeavours entails the identification of venom compounds, encompassing their compositional diversity (e.g., protein families), variability (e.g., intra- and inter-species, sex-linked, seasonal, environmental), evolutionary traits, mode of action, and toxicity attributes (e.g., neurotoxicity, haemolytic potency, enzymatic activity, LD50, ED50, clearance rates). This initial step heavily relies on information found in several biological databases (Fig. 1).

The raw data are generally deposited in generalist repositories such as the Proteomics IDEntification (PRIDE) database for mass spectrometry data [16], or the DNA Data Bank of Japan (DDBJ), the European Nucleotide Archive (ENA), and the National Center for Biotechnology Information (NCBI) GenBank for nucleic acid data. Nucleotide sequences can also be found in venom-specific databases like ArachnoServer [17] and ConoServer [18], which additionally provide protein sequences, classification of gene superfamilies, cysteine frameworks, information on pharmacological activities of toxins, as well as sequence analysis tools (see following section). Amino acid sequences derived from direct sequencing, or from translated nucleotide sequences, are mostly available in two generalist databases, UniProtKB and NCBI protein. The Tox-Prot annotation project of UniProtKB/Swiss-Prot provides access to venom protein sequences and links to additional web-resources [19]. Considering tools, UniProtKB supports BLAST searches (otherwise directly available on the NCBI website),

sequence alignment, searches for similar proteins, and links to various features in the Expasy Resource Portal [20]. The species from which the data originate are generally reported in the metadata and linked to taxonomy databases such as NCBI or UniProtKB Taxonomy (Table S1).

The three-dimensional (3D) structure of peptides and proteins is important to understand their function and mode of interaction with their molecular targets. The most comprehensive databases holding structural information are the Research Collaboratory for Structural Bioinformatics Protein Data Bank (RCSB PDB) [21], the Biological Magnetic Resonance Data Bank (BMRB) [22], and the Electron Microscopy Data Bank (EMDB) [23]. The structures in PDB are primarily determined through X-ray crystallography or nuclear magnetic resonance (NMR) spectroscopy, and increasingly by cryo-electron microscopy (cryo-EM), although the latter is only from molecules or molecular complexes with masses less than 100kDa. BMRB is a database of NMR spectroscopic data from peptides, proteins, nucleic acids, and other biologically relevant molecules, while EMDB archives 3D maps of biological specimens from transmission electron microscopy experiments. Cryo-EM holds great potential for investigating toxin-receptor binding [24,25]. Visualization of toxin 3D structures is provided in ArachnoServer and ConoServer, as well as in UniProtKB. Additionally, the AlphaFold Protein Structure Database [26] provides access to over 200 million 3D structures predicted by AlphaFold, an Artificial Intelligence (AI) system developed by Google DeepMind based on a neural network model [27].

A wide array of specialised databases for researchers interested in exploring biological pathways (e.g., the Kyoto Encyclopedia of Genes and Genomes (KEGG) [28]), gene function classification (e.g., the Gene Ontology (GO) Resource [29]), or more specific information on compounds (e.g., PubChem [30], KaliumDB [31], ScrepYard, KNOTTIN [32,33]) are discussed in the sections below and listed in Table S1.

Currently, information on venoms and toxins is dispersed across a multitude of resources, both generalists and specialists, each offering varying types of data and occasionally resulting in redundancy. This scenario presents both advantages and disadvantages. On one hand, the

proliferation of openly accessible data represents a goldmine for basic as well as applied research. Conversely, differences in data formats and content between disparate sources makes it challenging to aggregate information and sometimes results in inconsistencies. For instance, the annotation related to the mature and precursor sequence of a toxin might differ between a generalist database like UniProtKB, which provides the amino acid sequence of a whole gene, and a venom-specialist database like Arachnoserver or ConoServer, which is instead focused on reporting the active, mature sequence [34].

An additional inconvenience in the database landscape is that some have become obsolete (e.g., SCORPION2 [35]), while others offer limited utility (e.g., ATDB [36] primarily available in Chinese), or are at times unavailable (e.g., ArachnoServer), highlighting the need to constantly curate the available databases [34]. Nonetheless, enduring venom-specific databases and resources include ConoServer, VenoMS [37], T3DB [38], or UniProtKB/Tox-Prot. Furthermore, VenomZone is a free web resource that provides information on venoms from six major venomous taxa (i.e., snakes, scorpions, spiders, cone snails, sea anemones and insects), as well as on their molecular targets. Information can be browsed through pages on taxonomy, activity, and venom protein families, with links to the corresponding UniProtKB/Tox-Prot page.

Many of the aforementioned websites include some tools for predicting mature peptide boundaries, pharmacological activity, theoretical molecular mass, etc., while generalists web-based portals (e.g., Expasy [20] and Galaxy [39]) provide comprehensive resources for the analysis of gene expression data, structural biology, text mining, machine learning, and more.

1.2 Resources in genomics

Genomics is increasingly playing a central role in venom research. The advancements and decreasing costs of sequencing technologies have facilitated the availability of genome data from venomous species; consequently, genomics has become indispensable for elucidating the complexity of venom-related genes. Indeed, genomic information is crucial to assess

whether divergence in venom composition among species or populations arises from variation in gene copy number, nucleotide sequence, or regulation of gene expression [40–43].

One major advantage of genome data is that it eliminates artefacts from *de novo* proteo-transcriptomics, providing highly accurate results for predicting venom genes and identifying gene and protein variants, including all related transcript and protein-based modifications [44]. To achieve this, transcriptomic data can be assembled using a genome-guided transcriptome assembly approach (e.g. Trinity assembler [45]). Typically, the preferred method for creating genomes is to map transcripts against the genome sequences (scaffolds) with aligners such as BOWTIE2 [46], and splice aware tools like HISAT2 [47], STAR and Tophat2 [48,49] (although no longer supported). High-quality or reference genomes are generally annotated using transcriptomes from multiple tissue samples, comprehensively identifying most gene variants, which is especially relevant to properly characterise multigene families like many venom proteins [44].

Generating genomes involves using a plethora of tools and software, primarily command line-based due to the specificity, computational demands, and challenges associated with genome analysis [50,51]. Several pipelines have been developed by genome consortia, and the recently developed automated pipeline in Galaxy is expected to revolutionise the pace of reference genome production and annotation [52].

Resources and tools related to genomic data are currently not widely available in a venom-related context. However, there are several web resources for accessing genomes, with NCBI Genome being the primary platform that provides genomic data in conjunction with their respective publications. While NCBI offers a comprehensive collection of genomes, there are some thematic databases, such as Ensembl Metazoa, which focuses specifically on metazoan reference genomes and offers more tailored data and information [53]. Additionally, Ensembl provides cross-genome resources, annotations, syntenies, and other features, with the added benefit of being more accessible than NCBI through a server and application program interface (API) service. Many genome sequencing consortia, such as G10K, GIGA, i5K, B10K, VGP, EBP, DToL, T2T, and ERGA provide pre-publication information on their planned genomes

through dedicated websites, often including unpublished data [54–60]. For example, GenomeArk houses hundreds of high-quality reference genomes and assembly data.

Arguably, the venomous organisms benefitting of the richest genomic resources are Cnidaria (sea anemones, corals, hydroids, and jellyfish). The original reason for the construction of these datasets was the use of several cnidarian species as models for evolutionary developmental biology ('evo-devo') [61–64] and the specific importance of reef-building corals for marine ecology [65–67]. The availability of these chromosome-scale assemblies, along with rich datasets on small RNA sequencing [68,69], ChIP-seq of histone modification marks and transcriptional regulator proteins [70,71] for several key species, makes them an excellent resource for studying venom regulatory genomics and evolution. Some of these data can be easily access through the SIMRbase genome portal of the Stowers Institute for Medical Research and the *Hydra* 2.0 Genome Project Portal of the National Institute of Health (NIH).

Despite these advancements, challenges persist in annotating and analysing toxin-coding genes, as many venom components are part of large, multigene families, and gene comparison tools typically perform better for single-copy genes. Recent studies have demonstrated that the comparative genomics approach, which involves analysing the genome structure and arrangements of genes and their flanking regions across multiple species, known as micro-synteny, is the most effective method for unambiguously unravel the origin and evolution of many understudied multigene venom protein families or short toxin genes [44,72–74]. Another challenge is that many venom gene families are poorly studied and known, with misleading naming conventions often implying phylogenetic relationships based on similar allergenic responses in bioactivity tests (e.g. venom allergens). Therefore, availability of a dedicated database based on phylogenetic relationships rather than naming conventions would be valuable for analysing venom gene families. An example of a similar database is PhylomeDB, a catalogue of gene phylogenies (phylomes) with multisequence alignments, phylogenetic trees, and ortholog predictions [75]. A promising specialised new resource is ToxCodAn-Genome, an automated pipeline for annotating toxin genes in genomes [76]. While it relies on prior knowledge of venom genes, and it has been tested on a set of

well-known venomous lineages, it still neglects rare venomous taxa and more species-specific gene families.

A branch of biology that is increasingly being explored for insights into venom production and phenotype changes is epigenetics [40,77], the study of heritable traits occurring without DNA change (e.g., DNA methylation, histone modifications, chromatin architecture, non-coding RNA). Such changes are not erased by cell division, regulating gene expression, and altering cellular/physiological phenotypic traits influenced by environmental factors. A popular web-based genomic data exploration tool that provides visualisation, integration, and analysis of epigenomic datasets is the WashU Epigenome Browser [78]. This browser enables the interaction of 1D (genomic features), 2D (Hi-C data), 3D (chromatin structure), and 4D (gene/genomic regions as a function of time) data assessment, serving and expanding the data hubs from large consortia such as 4DN, Roadmap Epigenomics, TaRGET and ENCODE. However, it currently does not include any venomous taxa.

1.3 Resources in transcriptomics

RNA-Seq is one of the most employed strategies used to characterise venom components by sequencing mRNA from dissected venom glands. This technique enables the acquisition of complete precursor sequences, which can then be used to build a custom database for mass spectrometry-based searches of crude venom (proteo-transcriptomics). Although genomes from venomous organisms are now becoming available, *de novo* transcriptome assembly (often coupled with subsequent proteome analysis) remains the most common method to describe venom compositions, and for identifying novel toxin isoforms.

Due to the high computational demands of this process, most transcriptomics analyses are conducted on workstation computers, high performance clusters, or via cloud computing, and therefore utilise command-line tools. The most widely used assembler for venom gland transcriptomes is undoubtedly Trinity [45] and its companion Trinotate pipeline [79], which predicts coding regions and searches for homology against multiple databases. However, as of March 2024, Trinotate is no longer under active development or support. There are also

more bioinformatics knowledge-wise demanding multi-assembly pipelines that combine different assemblers and cover a larger space of gene models and reconstructed transcripts, one example is the Oyster River pipeline [80].

Functional annotation is typically performed manually through BLAST searches of translated amino acid sequences against UniProtKB, NCBI RefSeq, and other relevant databases (Table S1), along with domain searches using tools like HMMER [81] or InterProScan [82] against Pfam [83], CDD [84], or own custom databases (e.g.,[85]). To facilitate the identification of toxins, several predictor tools have been developed specifically for venom components. Some of these pipelines can be run locally from the command-line, e.g., Venomix [68], ToxClassifier [87], TOXIFY [88], and DeTox [89], while others, such as ToxDL [90], ConoPrec on ConoServer [18], among others (Table S1), can be run online through web interfaces where the translated amino acid sequences can be directly uploaded. Additionally, transcripts can be functionally annotated with Gene Ontology (GO) terms using online deep learning approaches such as Pannzer2 [91], and filtering for transcripts annotated with terms like ‘toxin activity’ or ‘modulation of process of another organism’.

While most transcriptomics studies on venomous animals focus on venom glands, comparative transcriptomics, which compares gene expression between venom glands and other tissues, provides further valuable insights. For instance, this approach can help with the annotation of a transcript as a venom protein, since toxin genes are generally uniquely or predominantly expressed in venom glands. Additionally, it helps identify pathways and genes involved in venom component biosynthesis and secretion [74–76]. After transcript quantification using command-line tools like Kallisto [95], differential expression analysis can be performed in R using various packages (e.g., edgeR [96]). The resulting list of venom gland upregulated genes can be subjected to enrichment analysis for GO terms and KEGG pathways, revealing chaperones and other proteins important for protein folding and maturation, or those secreted with toxins to facilitate their targeting.

RNA-Seq data, both raw and processed, can be archived in NCBI. Raw reads are deposited directly in the SRA archive, or through the European Nucleotide Archive (ENA) either

interactively or through the command-line, while assemblies can be archived in the Transcriptome Shotgun Assembly (TSA) sequence database, although it does not accept sequences below 200bp. Unlike the compulsory raw data submission, assemblies are not mandatory in most journals, and are therefore often not uploaded or published as supplementary data. Gene expression quantifications can be uploaded on the NCBI Gene Expression Omnibus (GEO) archive.

Archiving sequencing and gene expression data is crucial and highly recommended for ensuring their accessibility and reproducibility. By making the assemblies and the expression levels of the corresponding transcripts freely available, researchers can prevent the duplication of effort and unnecessary re-assembly and mapping of raw reads, allowing others to readily access and utilise this essential information for their own studies.

1.4 Resources in proteomics and peptidomics

Proteomics analysis plays a crucial role in venom research, as animal venoms are mostly composed of peptides and proteins. Mass spectrometry (MS) methods are commonly employed to identify venom components using two main approaches: bottom-up and top-down proteomics [14]. In bottom-up proteomics, venom components are enzymatically digested, and the resulting peptides are individually analysed by tandem MS. Conversely, top-down approaches analyse intact venom proteins without any prior fragmentation, necessitating high-resolution MS instruments. In both approaches peptides and proteins are identified through database-based or *de novo* searches. A database-based search matches spectra against an existing database, often derived from venom gland *de novo* transcriptome assembly or from other aforementioned datasets, while a *de novo* search infers peptide sequences directly from the mass spectra without relying on prior genomics or transcriptomics data [97]. Advancements in bottom-up proteomics have led to the development of user-friendly tools, democratising complex data analysis. Similar to genomics and transcriptomics, proteomics analyses on the raw data are mostly performed locally or on a computer cluster, while online resources are applied for downstream analyses.

For bottom-up proteomics, prominent proprietary database search engines like Mascot [98] and PEAKS DB [99] are commonly used for venom protein identification. Additionally, software tools like ProteomeDiscoverer [100] integrate multiple search algorithms such as Sequest [101], Mascot, and Byonic [102], for peptide identification and protein characterisation. Freely available platforms including pFind 3 [103], MSFragger [104], and PeptideShaker [105], offer powerful tools for identifying venom components and characterising post-translational modifications (PTMs). Other software solutions like MaxQuant [106] and Skyline [107] enable identification and quantification of venom proteins using data-dependent acquisition (DDA) methods. To overcome the limitation of DDA, platforms such as DIA-NN [108] and MaxDIA [109], both freely available, use data-independent acquisition (DIA) methods [110]. In contrast to database-based searches, *de novo* sequencing software like Novor [111], and pNovo [112] facilitate fast and accurate peptide sequencing, although it can be challenging for complex spectra and peptides with extensive PTMs.

Top-down approaches aim to characterise entire toxins, including their isoforms and PTMs, and have recently been applied to venom research [113]. In database-based searches, software such as OpenMS [114], MZmine [115], MS-Deconv [116], and Msconvert [117], are commonly used for deconvoluting complex data. Additionally, MS-Align+ [118], MASH Suite [119], pTop [120], and TopMG [121] allow for high-throughput and automated protein sequence matching of multiple isoforms with high-confidence. For *de novo* searches, licence-based software like PEAKS (Bioinformatics Solutions Inc.) and ProSight PC (Thermo Fisher Scientific) are generally used, as well as free academic licences for TopPIC [122] and Informed-Proteomics [123].

Artificial Intelligence (AI) tools are emerging in proteomics to predict protein structures, pharmacological properties, and interaction partners. Toxin-specific web server tools include ToxinPred [124], ToxinPred2 [125], and ToxClassifier [87] (although unavailable as of April 2024), while non-toxin-specific platforms include Peptide Ranker [126] and PEP-FOLD3 [127], which utilise machine learning algorithms to predict and design peptides from amino acid sequences. The newest version of PEP-FOLD4 [128] accounts for pH conditions and salt

concentration conformations, which are critical parameters for accurate structure prediction. Well known servers based on machine learning approaches include AlphaFold2 [27], available in ColabFold [129], RoseTTAFold [130], and RaptorX [131] which are based on PDB structures, multiple sequence alignments and specific algorithms to learn the backbone conformations and side chain-side chain contacts. However, limitations exist, particularly with the accuracy of predictions when signal peptides, pro-peptides, or PTM positions are not specified in the input amino acid sequence. Despite challenges, AI tools offer promising capabilities in predicting unknown protein structures.

Raw proteomics data can be deposited in repositories like PRIDE [16] and Mass Spectrometry Interactive Virtual Environment (MassIVE) [132], which play a crucial role in facilitating collaboration and reproducibility. Additionally, MassiVE offers tools for re-analysing spectral datasets, compare results and more.

1.5 Resources in metabolomics

The main objective of metabolomics is to identify and quantify the metabolites that exist in biological fluids, cells, and tissues. Amines, organic acids, steroids, alkaloids, and sugars are considered as the substances of the metabolome. To date, the elucidation of metabolite structures is mainly performed by studying the literature and comparing the MS/MS spectra of related metabolites. Comprehensive databases include the Human Metabolome Database (HMDB) [133] and KEGG [28], which offer different qualitative and quantitative data for human metabolites and information about metabolomic pathways. HMDB is currently the database containing the largest data collection of MS/MS fragmentation spectra of metabolites [133,134]. An interesting tool is offered by the Global Natural Products Social Molecular Networking (GNPS) [135], a web-based mass spectrometry ecosystem that aims to be an open-source and open-access knowledge base for community-wide organisation and sharing of raw, processed, or identified tandem mass (MS/MS) spectrometry data. GNPS aids in identification and discovery throughout the entire life cycle of data; from initial data acquisition to post publication.

The only existing venom-specialist metabolite database is VenoMS [37], which focuses on low molecular mass metabolites from spider venoms. VenoMS gathers known structures of spider venom metabolites and offers a fragment ion calculator (FRIOC) for the prediction of fragment ions for the linear polyamine derivatives. This website can be considered complementary to *ArachnoServer.* Despite its usefulness, this resource is limited to spiders, and is not included in the typical automated MS analyses.

A suggestion for a future endeavour could be to extend the content of VenoMS to other venomous organisms and create a more comprehensive online database of venom metabolites. As venom metabolomics is still in its infancy, challenges rely mostly in the chemical identification of metabolites and the integration with data from other omics platforms.

1.6 Resources in translational research

The vast biotechnological and biomedical potential of animal venoms and toxins is undeniable, with well documented bioactivities ranging from analgesic, immunomodulatory, anticancer, antimicrobial and antiparasitic properties [2–5,136]. This potential translates into a growing number of venom-derived drugs, with already 11 approved by the FDA & EMA, and many more in preclinical or clinical development. Beyond medicine, venom toxins hold promise for diagnostics, nanopore-based sensing, agrochemicals, and cosmetics [8,10–12,137]. However, despite the evident opportunities, the translation of basic research into concrete applications is a lengthy process that requires the generation of a variety of data and access to a wide array of different tools and databases. In this section, we provide an overview of the available resources pertinent to venom and toxin research from a biomedical and translational perspective.

In a typical workflow for venom component discovery, the first step involves candidate identification. This can be achieved by generating new data by means of genomics or proteo-transcriptomics analysis or by mining existing databases. Typical databases include ArachnoServer, Toxin and Toxin Target Database (T3DB), PubChem, UniProtKB/Swiss-Prot among others (Table S1). T3DB is particularly useful as it combines detailed toxin data with

comprehensive receptor information, molecular and biological properties, toxin effects, and potential therapeutic applications [38]. For peptide-based cancer research, CancerPPD4 [138], canSAR [139], ApInAPDB [140], PaccMann [141], and EviCor [142] provide platforms for the exploration of the mechanism of action, function, binding target, affinity, structural information, and other physicochemical features of peptides. Furthermore, they offer AI-based predictions of anticancer compound sensitivity and other properties to inform drug discovery. A comprehensive database useful in translational research was the discontinued VenomKB [143], which included data on venom's molecular components and their potential applications in drug discovery and development.

The databases can be mined manually to select a list of potential candidates which can be further screened using the prediction tools mentioned earlier. Alternatively, databases can be used to build machine learning models based on Random Forest, Support Vector Machine, or Artificial Neural Networks algorithms, which can process a vast amount of data and identify patterns to predict potential drug targets. This first crucial step of target identification poses a challenge in venom research as the toxin information is scattered across several databases. Thanks to the advent of the Semantic Web (SW), the tedious process to manually mine different life science databases can be significantly reduced [144]. SW provides a common framework that enables data to be shared and reused across different data sources. Combining and querying these data sources are possible by using a standard semantic query language like SPARQL. A solution to meaningfully access the databases containing animal venom information is to federate them by applying SW technologies that enable semantic queries across them [145]. For instance, currently UniProtKB and PubChem can be jointly queried by writing a single federated SPARQL query [146].

Once potential candidates are characterised, further steps include prediction of molecular targets and interactions with the toxins. Databases such as the mousephenotype.org for mammals [147], zfin.org for zebrafish [148], and flybase.org for insects [149] can be explored for predicting the effects of toxin intervention on systemic level and specific regulatory functions, ant to identify promising pharmaceutical or bioinsecticides targets. Web-based

prediction tools for molecular docking include SwissDock [150], the more recently developed PPI-Affinity model [151], as well as the CAMP model [152] to elaborate on target-predictions for peptides and proteins. Molecular docking and molecular dynamics simulation models such as quantitative structure–activity relationship (QSAR), quantitative structure–property relationship (QSPR) analysis, pharmacophore modelling and iBitter-SCM are frequently used to decipher peptide and protein interactions [153].

Once a lead compound has been identified and selected, it can be modified to have unique and desirable properties, for instance to modulate their target selectively and induce a therapeutical rather than a harmful toxic effect [154]. ToxinPred and ToxinPred2 include tools to design all possible single mutant analogues of a peptide and predict whether they are toxic or not, and to optimise the peptide sequence to get maximum, minimum, and desired toxicity. In addition, ToxinPred offers users to calculate various physicochemical properties.

While the approaches delineated above facilitate the search among known venom compounds, enduring challenges remain the prediction of toxins with undescribed new mechanisms of action and the identification of potential for off-target effects that might limit the usefulness of the molecule as a putative therapeutic drug [155], although current machine learning algorithms present promising avenue for the discovery of molecules with novel activities. Despite the potential benefits, it is important to acknowledge that the principles of open science may not always be guaranteed in translational and applied research, often due to confidentiality agreements associated with preliminary studies on toxin activity prediction and application.

1.7 Resources in antivenom production and administration

Scientists working in the field of antivenom research are typically interested in a variety of information. These span from the geographic distribution of the venomous species to their venom composition and variation, toxin structure and bioactivity, which all impact antivenom efficiency. Most of the resources related to this kind of information have been already

discussed in previous sections and are listed in Table S1, therefore here we focus on the resources available for antivenom producers.

A first important resource is represented by the World Health Organization (WHO) guidelines, which provides comprehensive and important manuals for antivenom manufacturers on the design, production, control, and regulation of high-quality antivenom immunoglobulins. These guidelines are regularly updated to provide to national regulatory bodies with framework guidance for securing the products they offer. Technical bulletins, reports and documents are also available on the WHO website. Within the scope of WHO web resources, in addition to pharmacopoeia requirements, current antidote production, and especially the improvement of studies and technologies carried out under GMP quality system conditions, are ensured. Additionally, WHO manages the snakebite information and data platform as part of the 2019-2030 global strategy for the prevention and control of snakebite envenoming, which is within the scope of neglected tropical diseases by WHO. This web source platform is part of a collaboration between the departments for the control of Neglected Tropical Diseases (WHO/NTD) and the Dissemination of Data for Impact and analytics (WHO/DDI). Another data source created for easy access to antivenom in cases of envenoming caused by poisonous animals is the Munich AntiVenom INdex (MAVIN) created by the Munich Poison Center. MAVIN gathers a list of venomous animals, antivenom holding centres, antivenoms and correlated information.

In addition to international web resources such as WHO and MAVIN, some countries have developed national web resources to help staff at zoos and aquariums managing the supply of antivenom and finding the right antivenom when they need it. For instance, an online Antivenom Index was created in 2006 by the Association of Zoos and Aquariums (AZA) and the America's Poison Centers (previously known as American Association of Poison Control Centers – AAPCC). The University of Arizona College of Pharmacy is currently responsible for maintaining, updating, and hosting this index. However, only representatives of poison control centres and AZA-accredited institutions have access to the Antivenom Index.

1.8 Resources in clinical toxinology

Several freely available resources offer information on venoms and venomous animals, which are relevant to clinical toxicologists and toxinologists. A central resource is the 'Clinical Toxinology Resources' website which provides comprehensive information on venomous and poisonous animals, plants and mushrooms from around the world (Table S1). This repository receives support from experts around the world, and it features a searchable database that allows users to find specific organisms by common or scientific names, family, country, or region. Another useful resource is PubChem which gathers information on chemical structure, chemical and physical properties, biological activity, toxicity, medical management guidance, among others.

Most clinical toxinology and toxicology databases cater specifically to poison centres and are accessible only to registered healthcare professionals. Nonetheless, some are reachable upon subscription fees and may offer free or reduced-cost access, particularly for users in low-income countries. For instance, AfriTox offers online and offline versions, primarily for registered healthcare professionals, with subscription-based access. This database focuses on substances, including venomous exposures, from an African perspective. The Merative Micromedex® POISINDEX® System is widely utilised worldwide, especially in North America, and provides both summary and in-depth clinical toxicology information, including details on venomous animals, through subscription-based access. Another useful resource is TOXBASE, produced by poison specialists and medical toxicologists, which offers advice on toxin features and exposure management to toxins and venomous animals. While primarily accessible to UK healthcare professionals, TOXBASE is also utilised internationally, with special arrangements for certain countries. Lastly, TOXINZ provides information and treatment guidelines, including venomous animal exposures. While primarily designed for use in New Zealand, TOXINZ is accessible in other countries through paid subscriptions.

2. Challenges, needs and perspectives of web resources in venom research

The survey that we conducted within the framework of the EUVEN COST Action [15], although representing only a sample of the worldwide venom research community, provided important insights into the challenges and needs of researchers and clinicians working with animal venoms or toxins. Here, we have summarised and discussed them.

2.1 Challenges

Many scientists in the venom research community expressed disappointment due to the bottleneck caused by the limited expertise in bioinformatics and data management, especially concerning the handling of complex '-omics' pipelines essential for cutting-edge research. Despite the improvements in accessibility offered by databases, there is still a demand for more user-friendly interfaces that seamlessly integrate data and tools into existing pipelines, facilitating the translation of research findings into clinical applications. However, achieving a unified Graphical User Interface (GUI) software is not easy due to the variety, volume, and complexity of current data, requesting storage on servers alongside the necessary analysis tools. Toxinologists are encouraged to collaborate with bioinformaticians and relevant technology experts in cross-disciplinary projects. Initiatives like EUVEN and organisations such as the Swiss Institute of Bioinformatics provide support and facilitate collaborations by offering access to databases of researchers and their corresponding expertise.

Another challenge faced by venom researchers, particularly those involved in applied aspects like drug discovery, was related to the scattered and diverse nature of information about venoms and toxins across several databases. This issue is not unique to venom researchers but is prevalent among biologists. As the production of biological and health data continues to exponentially grow, so does the number of databases [156]. However, querying is still largely limited to a single database at a time, making it difficult to integrate multiple data types to answer complex biological questions [144]. A step forward in addressing this challenge is the adoption of query languages like SPARQL to search across different databases and perform data manipulation tasks such as exploration, extraction, and annotation. Furthermore, to effectively manage and analyse datasets, standardised terminologies and classification

systems are essential. Ontologies and thesauri serve as structured vocabularies that provide a common language for annotating and organising biological information (e.g. the Gene Ontology, the UniProtKB/Swiss-Prot controlled vocabularies). Even though the use of such resources is generally well consolidated along the research pipelines, often different terms are employed to denote the same concept, or conversely, the same term is utilised to represent multiple concepts across web resources, thereby hindering interoperability (A.T. personal communication). For instance, in Ontobee [157], a catalogue and web-based linked data server for semantic terminologies, the term “venom” is described differently in eight ontologies. This highlights the need for mapping terms between the semantic resources commonly used in the field.

As venom omics data accumulate, the challenge evolves from basic descriptive comparative findings to the more sophisticated task of integrating multi-omics data. This approach ultimately aims to gain a comprehensive understanding of the complexity of biological systems and their underlying mechanisms. To this end, data standardisation, advanced computational methods (e.g., machine learning techniques), and interpretation of diverse data types is key to provide meaningful insights. While multi-omics integration tools are currently applied in studying complex human diseases [158], they hold great promise for deciphering equally complex venom phenotypes.

2.2 Needs

Despite the abundance of databases containing information on animal toxins, some data remains disorganised and inaccessible due to a lack of structured datasets. For the data to be accessible through query languages, databases need to be machine-readable, meaning they must be formatted in a way that can be processed by software tools. This is also crucial for full implementation of the FAIR principles [159]. The Resource Description Framework (RDF) for instance, is a SW standard data model adopted by many databases for sharing and linking data. Data in RDF can be queried, retrieved, and manipulated using the SPARQL language, which has the advantage that it is graph-based, thus allowing users to join data from multiple,

diverse sources (in contrast to SQL which is a table-based query language). Therefore, there is a need to standardise the structure of databases to run queries on animal venoms and toxin research across them. Furthermore, it is advisable to utilise existing ontologies and incorporate controlled terms already in use, or map redundant terms among them. This can be facilitated by searching existing terms in semantic resource catalogues such as Ontobee, the Ontology Lookup Service (OLS), or BioPortal [144,160]. This practice prevents unnecessary duplications, reduces redundancy, and enhance data reusability and interoperability, which is particularly relevant to a high multidisciplinary field like venom research.

Another issue raised by the venom research community is the absence of a repository for protocols and methods for recombinantly producing or chemically synthesising venom peptides, which would benefit researchers by preventing redundant protocol optimisation efforts, especially in the case of toxins difficult to refold. Additionally, there is a need for a centralised, non-profit database of biological materials related to venoms and natural or engineered toxins stored or generated in research institutes, similar to plasmid repositories or even catalogues for museum specimens, to aid researchers in accessing pre-existing materials for their own studies.

Ensuring data and information accessibility and standardization to the research and clinician communities and the public remains crucial, as discussed in previous sections. The importance of making these data publicly available is further emphasised by the FAIR principles [159] and the recent European Open Access policies [161], which advocate for open access not only to publications but also to all underlying data. Addressing these needs and challenges will require collaboration and concerted efforts from researchers, clinicians, and organisations to advance venom research and its applications.

2.3 Perspectives on a unified venom web resource

Steps toward satisfying the needs of the venom research community include the creation of a venom-specific resource containing detailed information on venomous species and their

venoms and toxins. This database could encompass genome architecture and function of venomous species, venom gland transcriptomes, toxin genes and their translated amino acid sequences, PTMs, 3D structures, pharmacological activities and toxicity levels, molecular and cellular targets, mechanisms of action, coupled with ecological and evolutionary information of the corresponding species (e.g., diet and geographical distribution). By consolidating such diverse information into a single resource or interface uniting a range of resources, scientists working in the interdisciplinary field of animal venoms and toxins would have a valuable tool at their disposal. It would enable them to access both general and specific information on a vast number of venomous species and toxins and would decrease the time spent on extensive literature searches.

Such a resource could also significantly contribute to venom research by facilitating the classification of venom proteins, aiding in the design of peptides with desired pharmacological properties, and identifying potential interactions. However, the creation and maintenance of such a platform would present considerable challenges, requiring substantial workforce, financial resources, and international interdisciplinary collaborations to ensure its continual updates and accuracy.

An existing resource like VenomZone could serve as a starting point toward realising this unified resource. However, significant expansions would be necessary to incorporate the additional data proposed. A promising initiative is the interactive table that we have compiled within the framework of this work and made available on the VenomZone website (https://venomzone.expasy.org/10897). It includes current web resources relevant to venom research in an interactive way. It therefore represents a positive step toward creating a comprehensive and accessible resource for the entire venom research community.

Conclusions

- Modern venom research is a multidisciplinary field resulting in the generation and analysis of highly diverse datasets.

- Currently, information on venom and toxin data is scattered across different resources, ranging from generalist to specialised platforms.
- Most multi-omics analyses are performed using software and command-line tools that require advanced computational and command-line skills, while most available web resources mainly offer downstream analyses.
- One of the core challenges is accessing and providing information across the different databases. There is an urgent need to establish standards to facilitate interoperability and allow seamless querying of animal venom and toxin research across platforms.
- Progress towards meeting the needs of the venom research community requires the establishment of a dedicated venom-specific resource. VenomZone, together with our newly curated site on demanded tools and resources, represents an important first step towards this goal.

Declarations

Data Availability

Not applicable.

Competing Interests

The authors declare that they have no competing interests.

Funding

This work is funded by the European Cooperation in Science and Technology (COST, www.cost.eu) and based upon work from the COST Action CA19144 European Venom Network (EUVEN, https://euven-network.eu/). This review is an outcome of EUVEN Working Group 4 ("Web resources") led by A.A. and G.Z.. G.Z. was supported by the European Union's Horizon 2020 Research and Innovation program through Marie Sklodowska-Curie Individual Fellowship (grant agreement No. 845674). B.M.v.R. acknowledges funding from the German

Science Foundation (DFG RE3454/6–1). M.P.I. was supported by the TALENTO Program by the Regional Madrid Government (#2022-5A/BIO-24228) and the grant (#PID2021-126691OB-I00) funded by MICIU/AEI/10.13039/501100011000011033 and by the European Union. F.J. was supported by the Swiss federal government through the State Secretariat for Education, Research, and Innovation (SERI). R.V. acknowledges the Portuguese Foundation for Science and Technology (FCT), QREN, FEDER, and COMPETE for funding to the Institute of Biomedicine (iBiMED) (UIDB/04501/2020, PO-CI-01-0145-FEDER-007628).

Authors' contributions

Major conceptualisation by M.V.M., G.A., G.Z., A.A., and B.M.v.R.. G.Z. and F.J. analysed the survey data. M.L.C., F.J., P.M., and B.M.v.R conceptualised the graphics, B.M.v.R. made the figures. G.Z. lead the writing of the manuscript. All the authors contributed to the main text. All the authors have read and agreed to the published version of the manuscript.

Acknowledgments

The authors thank Ronald A. Jenner for his valuable comments on an earlier version of the manuscript, Marc Robinson-Rechavi, Sébastien Moretti and Valentine Rech De Laval for their feedback on additional useful web resources.

References

1. Schendel V, Rash LD, Jenner RA, Undheim EAB. The diversity of venom: The importance of behavior and venom system morphology in understanding its ecology and evolution. Toxins. 2019;11:666.

2. Lewis RJ, Garcia ML. Therapeutic potential of venom peptides. Nat Rev Drug Discov. 2003;2:790–802.

3. Holford M, Daly M, King GF, Norton RS. Venoms to the rescue. Science. 2018;361:842–4.

4. Herzig V, Cristofori-Armstrong B, Israel MR, Nixon SA, Vetter I, King GF. Animal toxins — Nature's evolutionary-refined toolkit for basic research and drug discovery. Biochemical Pharmacology. 2020;181:114096.

5. Waheed H, Moin SF, Choudhary MI. Snake venom: From deadly toxins to life-saving therapeutics. Current Medicinal Chemistry. 2017;24:1874–91.

6. Talukdar A, Maddhesiya P, Namsa ND, Doley R. Snake venom toxins targeting the central nervous system. Toxin Reviews. 2023;42:382–406.

7. Oliveira AL, Viegas MF, da Silva SL, Soares AM, Ramos MJ, Fernandes PA. The chemistry of snake venom and its medicinal potential. Nat Rev Chem. 2022;6:451–69.

8. Marsh NA. Diagnostic uses of snake venom. Pathophysiology of Haemostasis and Thrombosis. 2002;31:211–7.

9. Estevão-Costa M-I, Sanz-Soler R, Johanningmeier B, Eble JA. Snake venom components in medicine: From the symbolic rod of *Asclepius* to tangible medical research and application. The International Journal of Biochemistry & Cell Biology. 2018;104:94–113.

10. Windley MJ, Herzig V, Dziemborowicz SA, Hardy MC, King GF, Nicholson GM. Spider-venom peptides as bioinsecticides. Toxins. 2012;4:191–227.

11. King GF, Hardy MC. Spider-venom peptides: Structure, pharmacology, and potential for control of insect pests. Annual Review of Entomology. 2013;58:475–96.

12. Modahl CM, Brahma RK, Koh CY, Shioi N, Kini RM. Omics technologies for profiling toxin diversity and evolution in snake venom: Impacts on the discovery of therapeutic and diagnostic agents. Annu Rev Anim Biosci. 2020;8:91–116.

13. Dutertre S, Lewis RJ. Use of venom peptides to probe ion channel structure and function. Journal of Biological Chemistry. 2010;285:13315–20.

14. von Reumont BM, Anderluh G, Antunes A, Ayvazyan N, Beis D, Caliskan F, et al. Modern venomics—Current insights, novel methods, and future perspectives in biological and applied animal venom research. GigaScience. 2022;11:giac048.

15. Modica MV, Ahmad R, Ainsworth S, Anderluh G, Antunes A, Beis D, et al. The new COST Action European Venom Network (EUVEN)—synergy and future perspectives of modern venomics. GigaScience. 2021;10:giab019.

16. Perez-Riverol Y, Bai J, Bandla C, García-Seisdedos D, Hewapathirana S, Kamatchinathan S, et al. The PRIDE database resources in 2022: a hub for mass spectrometry-based proteomics evidences. Nucleic Acids Research. 2022;50:D543–52.

17. Pineda SS, Chaumeil P-A, Kunert A, Kaas Q, Thang MWC, Le L, et al. ArachnoServer 3.0: an online resource for automated discovery, analysis and annotation of spider toxins. Bioinformatics. 2018;34:1074–6.

18. Kaas Q, Yu R, Jin A-H, Dutertre S, Craik DJ. ConoServer: updated content, knowledge, and discovery tools in the conopeptide database. Nucleic Acids Research. 2012;40:D325–30.

19. Jungo F, Bougueleret L, Xenarios I, Poux S. The UniProtKB/Swiss-Prot Tox-Prot program: A central hub of integrated venom protein data. Toxicon. 2012;60:551–7.

20. Duvaud S, Gabella C, Lisacek F, Stockinger H, Ioannidis V, Durinx C. Expasy, the Swiss Bioinformatics Resource Portal, as designed by its users. Nucleic Acids Research. 2021;49:W216–27.

21. wwPDB consortium. Protein Data Bank: the single global archive for 3D macromolecular structure data. Nucleic Acids Research. 2019;47:D520–8.

22. Romero PR, Kobayashi N, Wedell JR, Baskaran K, Iwata T, Yokochi M, et al. BioMagResBank (BMRB) as a Resource for Structural Biology. In: Gáspári Z, editor. Structural Bioinformatics: Methods and Protocols. New York: Springer US; 2020. p. 187–218.

23. The wwPDB Consortium. EMDB—the Electron Microscopy Data Bank. Nucleic Acids Research. 2024;52:D456–65.

24. Haji-Ghassemi O, Chen YS, Woll K, Gurrola GB, Valdivia CR, Cai W, et al. Cryo-EM analysis of scorpion toxin binding to ryanodine receptors reveals subconductance that is abolished by PKA phosphorylation. Science Advances. 2023;9:eadf4936.

25. Nys M, Zarkadas E, Brams M, Mehregan A, Kambara K, Kool J, et al. The molecular mechanism of snake short-chain α-neurotoxin binding to muscle-type nicotinic acetylcholine receptors. Nat Commun. 2022;13:4543.

26. Varadi M, Anyango S, Deshpande M, Nair S, Natassia C, Yordanova G, et al. AlphaFold Protein Structure Database: massively expanding the structural coverage of protein-sequence space with high-accuracy models. Nucleic Acids Res. 2022;50:D439–44.

27. Jumper J, Evans R, Pritzel A, Green T, Figurnov M, Ronneberger O, et al. Highly accurate protein structure prediction with AlphaFold. Nature. 2021;596:583–9.

28. Kanehisa M, Goto S. KEGG: Kyoto encyclopedia of genes and genomes. Nucleic Acids Res. 2000;28:27–30.

29. Ashburner M, Ball CA, Blake JA, Botstein D, Butler H, Cherry JM, et al. Gene Ontology: tool for the unification of biology. Nat Genet. 2000;25:25–9.

30. Kim S, Chen J, Cheng T, Gindulyte A, He J, He S, et al. PubChem 2023 update. Nucleic Acids Research. 2023;51:D1373–80.

31. Krylov NA, Tabakmakher VM, Yureva DA, Vassilevski AA, Kuzmenkov AI. Kalium 3.0 is a comprehensive depository of natural, artificial, and labeled polypeptides acting on potassium channels. Protein Science. 2023;32:e4776.

32. Postic G, Gracy J, Périn C, Chiche L, Gelly J-C. KNOTTIN: the database of inhibitor cystine knot scaffold after 10 years, toward a systematic structure modeling. Nucleic Acids Research. 2018;46:D454–8.

33. Liu J, Maxwell M, Cuddihy T, Crawford T, Bassetti M, Hyde C, et al. ScrepYard: An online resource for disulfide-stabilized tandem repeat peptides. Protein Sci. 2023;32:e4566.

34. Jungo F, Estreicher A, Bairoch A, Bougueleret L, Xenarios I. Animal Toxins: How is complexity represented in databases? Toxins. 2010;2:262–82.

35. Tan PTJ, Veeramani A, Srinivasan KN, Ranganathan S, Brusic V. SCORPION2: a database for structure-function analysis of scorpion toxins. Toxicon. 2006;47:356–63.

36. He Q-Y, He Q-Z, Deng X-C, Yao L, Meng E, Liu Z-H, et al. ATDB: a uni-database platform for animal toxins. Nucleic Acids Res. 2008;36:D293–7.

37. Forster YM, Reusser S, Forster F, Bienz S, Bigler L. VenoMS—A website for the low molecular mass compounds in spider venoms. Metabolites. 2020;10:327.

38. Wishart D, Arndt D, Pon A, Sajed T, Guo AC, Djoumbou Y, et al. T3DB: the toxic exposome database. Nucleic Acids Res. 2015;43:D928-934.

39. The Galaxy Community. The Galaxy platform for accessible, reproducible and collaborative biomedical analyses: 2022 update. Nucleic Acids Research. 2022;50:W345–51.

40. Perry BW, Gopalan SS, Pasquesi GIM, Schield DR, Westfall AK, Smith CF, et al. Snake venom gene expression is coordinated by novel regulatory architecture and the integration of multiple co-opted vertebrate pathways. Genome Res. 2022;32:1–16.

41. Dowell NL, Giorgianni MW, Kassner VA, Selegue JE, Sanchez EE, Carroll SB. The deep origin and recent loss of venom toxin genes in rattlesnakes. Current Biology. 2016;26:2434–45.

42. Vonk FJ, Casewell NR, Henkel CV, Heimberg AM, Jansen HJ, McCleary RJR, et al. The king cobra genome reveals dynamic gene evolution and adaptation in the snake venom system. Proceedings of the National Academy of Sciences. 2013;110:20651–6.

43. Schield DR, Card DC, Hales NR, Perry BW, Pasquesi GM, Blackmon H, et al. The origins and evolution of chromosomes, dosage compensation, and mechanisms underlying venom regulation in snakes. Genome Res. 2019;29:590–601.

44. Drukewitz SH, von Reumont BM. The significance of comparative genomics in modern evolutionary venomics. Front Ecol Evol. 2019;7.

45. Grabherr MG, Haas BJ, Yassour M, Levin JZ, Thompson DA, Amit I, et al. Trinity: reconstructing a full-length transcriptome without a genome from RNA-Seq data. Nat Biotechnol. 2011;29:644–52.

46. Langmead B, Salzberg SL. Fast gapped-read alignment with Bowtie 2. Nat Methods. 2012;9:357–9.

47. Kim D, Paggi JM, Park C, Bennett C, Salzberg SL. Graph-based genome alignment and genotyping with HISAT2 and HISAT-genotype. Nat Biotechnol. 2019;37:907–15.

48. Kim D, Pertea G, Trapnell C, Pimentel H, Kelley R, Salzberg SL. TopHat2: accurate alignment of transcriptomes in the presence of insertions, deletions and gene fusions. Genome Biology. 2013;14:R36.

49. Musich R, Cadle-Davidson L, Osier MV. Comparison of short-read sequence aligners indicates strengths and weaknesses for biologists to consider. Front Plant Sci. 2021;12:657240.

50. Amarasinghe SL, Su S, Dong X, Zappia L, Ritchie ME, Gouil Q. Opportunities and challenges in long-read sequencing data analysis. Genome Biol. 2020;21:30.

51. Wang Y, Zhao Y, Bollas A, Wang Y, Au KF. Nanopore sequencing technology, bioinformatics and applications. Nat Biotechnol. 2021;39:1348–65.

52. Larivière D, Abueg L, Brajuka N, Gallardo-Alba C, Grüning B, Ko BJ, et al. Scalable, accessible and reproducible reference genome assembly and evaluation in Galaxy. Nat Biotechnol. 2024;42:367–70.

53. Cunningham F, Allen JE, Allen J, Alvarez-Jarreta J, Amode MR, Armean IM, et al. Ensembl 2022. Nucleic Acids Res. 2021;50:D988–95.

54. Rhie A, McCarthy SA, Fedrigo O, Damas J, Formenti G, Koren S, et al. Towards complete and error-free genome assemblies of all vertebrate species. Nature. 2021;592:737–46.

55. Koepfli K-P, Paten B, Genome 10K Community of Scientists, O'Brien SJ. The Genome 10K Project: a way forward. Annual review of animal biosciences. 2015;3:57–111.

56. Voolstra CR, Woerheide G, Lopez JV, COS GCS. Advancing genomics through the Global Invertebrate Genomics Alliance (GIGA). Invertebrate Systematics. 2017;31:1–7.

57. Lewin HA, Robinson GE, Kress WJ, Baker WJ, Coddington J, Crandall KA, et al. Earth BioGenome Project: Sequencing life for the future of life. Proc Natl Acad Sci USA. 2018;115:4325–33.

58. Formenti G, Theissinger K, Fernandes C, Bista I, Bombarely A, Bleidorn C, et al. The era of reference genomes in conservation genomics. Trends in Ecology & Evolution. 2022;37.

59. The Darwin Tree of Life Project Consortium. Sequence locally, think globally: The Darwin Tree of Life Project. Proceedings of the National Academy of Sciences. 2022;119:e2115642118.

60. Zhang G, Li C, Li Q, Li B, Larkin DM, Lee C, et al. Comparative genomics reveals insights into avian genome evolution and adaptation. Science. 2014;346:1311–20.

61. Zimmermann B, Montenegro JD, Robb SMC, Fropf WJ, Weilguny L, He S, et al. Topological structures and syntenic conservation in sea anemone genomes. Nat Commun. 2023;14:8270.

62. Kon-Nanjo K, Kon T, Horkan HR, Febrimarsa null, Steele RE, Cartwright P, et al. Chromosome-level genome assembly of *Hydractinia symbiolongicarpus*. G3. 2023;13:jkad107.

63. Chapman JA, Kirkness EF, Simakov O, Hampson SE, Mitros T, Weinmaier T, et al. The dynamic genome of *Hydra*. Nature. 2010;464:592–6.

64. Putnam NH, Srivastava M, Hellsten U, Dirks B, Chapman J, Salamov A, et al. Sea anemone genome reveals ancestral eumetazoan gene repertoire and genomic organization. Science. 2007;317:86–94.

65. Shinzato C, Shoguchi E, Kawashima T, Hamada M, Hisata K, Tanaka M, et al. Using the *Acropora digitifera* genome to understand coral responses to environmental change. Nature. 2011;476:320–3.

66. Baumgarten S, Simakov O, Esherick LY, Liew YJ, Lehnert EM, Michell CT, et al. The genome of *Aiptasia*, a sea anemone model for coral symbiosis. Proc Natl Acad Sci U S A. 2015;112:11893–8.

67. Bhattacharya D, Agrawal S, Aranda M, Baumgarten S, Belcaid M, Drake JL, et al. Comparative genomics explains the evolutionary success of reef-forming corals. Elife. 2016;5:e13288.

68. Grimson A, Srivastava M, Fahey B, Woodcroft BJ, Chiang HR, King N, et al. Early origins and evolution of microRNAs and Piwi-interacting RNAs in animals. Nature. 2008;455:1193–7.

69. Moran Y, Fredman D, Praher D, Li XZ, Wee LM, Rentzsch F, et al. Cnidarian microRNAs frequently regulate targets by cleavage. Genome Res. 2014;24:651–63.

70. Schwaiger M, Schönauer A, Rendeiro AF, Pribitzer C, Schauer A, Gilles AF, et al. Evolutionary conservation of the eumetazoan gene regulatory landscape. Genome Res. 2014;24:639–50.

71. Cazet JF, Siebert S, Little HM, Bertemes P, Primack AS, Ladurner P, et al. A chromosome-scale epigenetic map of the *Hydra* genome reveals conserved regulators of cell state. Genome Res. 2023;33:283–98.

72. Jackson TNW, Koludarov I. How the toxin got its toxicity. Front Pharmacol. 2020;11:574925.

73. Koludarov I, Velasque M, Senoner T, Timm T, Greve C, Hamadou B, Gupta DK, et al. Prevalent bee core venom genes evolved before the aculeate stinger and eusociality. BMC Biology. 2023;21:229.

74. Koludarov I, Senoner T, Jackson TNW, Dashevsky D, Heinzinger M, Aird SD, et al. Domain loss enabled evolution of novel functions in the snake three-finger toxin gene superfamily. Nat Commun. 2023;14:4861.

75. Fuentes D, Molina M, Chorostecki U, Capella-Gutiérrez S, Marcet-Houben M, Gabaldón T. PhylomeDB V5: an expanding repository for genome-wide catalogues of annotated gene phylogenies. Nucleic Acids Research. 2022;50:D1062–8.

76. Nachtigall PG, Durham AM, Rokyta DR, Junqueira-de-Azevedo ILM. ToxCodAn-Genome: an automated pipeline for toxin-gene annotation in genome assembly of venomous lineages. Gigascience. 2024;13:giad116.

77. Hogan MP, Holding ML, Nystrom GS, Colston TJ, Bartlett DA, Mason AJ, et al. The genetic regulatory architecture and epigenomic basis for age-related changes in rattlesnake venom. Proc Natl Acad Sci U S A. 2024;121:e2313440121.

78. Li D, Purushotham D, Harrison JK, Hsu S, Zhuo X, Fan C, et al. WashU Epigenome Browser update 2022. Nucleic Acids Res. 2022;50:W774–81.

79. Bryant DM, Johnson K, DiTommaso T, Tickle T, Couger MB, Payzin-Dogru D, et al. A tissue-mapped axolotl de novo transcriptome enables identification of limb regeneration factors. Cell Reports. 2017;18:762–76.

80. MacManes MD. The Oyster River Protocol: a multi-assembler and kmer approach for *de novo* transcriptome assembly. PeerJ. 2018;6:e5428.

81. Finn RD, Clements J, Eddy SR. HMMER web server: interactive sequence similarity searching. Nucleic Acids Res. 2011;39:W29–37.

82. Blum M, Chang H-Y, Chuguransky S, Grego T, Kandasaamy S, Mitchell A, et al. The InterPro protein families and domains database: 20 years on. Nucleic Acids Research. 2021;49:D344–54.

83. Mistry J, Chuguransky S, Williams L, Qureshi M, Salazar GA, Sonnhammer ELL, et al. Pfam: The protein families database in 2021. Nucleic Acids Research. 2021;49:D412–9.

84. Wang J, Chitsaz F, Derbyshire MK, Gonzales NR, Gwadz M, Lu S, et al. The conserved domain database in 2023. Nucleic Acids Res. 2022;51:D384–8.

85. Almeida D, Domínguez-Pérez D, Matos A, Agüero-Chapin G, Osório H, Vasconcelos V, et al. Putative antimicrobial peptides of the posterior salivary glands from the cephalopod *Octopus vulgaris* revealed by exploring a composite protein database. Antibiotics. 2020;9:757.

86. Macrander J, Panda J, Janies D, Daly M, Reitzel AM. Venomix: a simple bioinformatic pipeline for identifying and characterizing toxin gene candidates from transcriptomic data. PeerJ. 2018;6:e5361.

87. Gacesa R, Barlow DJ, Long PF. Machine learning can differentiate venom toxins from other proteins having non-toxic physiological functions. PeerJ Comput Sci. 2016;2:e90.

88. Cole TJ, Brewer MS. TOXIFY: a deep learning approach to classify animal venom proteins. PeerJ. 2019;7:e7200.

89. Ringeval A, Farhat S, Fedosov A, Gerdol M, Greco S, Mary L, et al. DeTox: a pipeline for the detection of toxins in venomous organisms. Briefings in Bioinformatics. 2024;25:bbae094.

90. Pan X, Zuallaert J, Wang X, Shen H-B, Campos EP, Marushchak DO, et al. ToxDL: deep learning using primary structure and domain embeddings for assessing protein toxicity. Bioinformatics. 2020;36:5159–68.

91. Törönen P, Medlar A, Holm L. PANNZER2: a rapid functional annotation web server. Nucleic Acids Res. 2018;46:W84–8.

92. Zancolli G, Reijnders M, Waterhouse RM, Robinson-Rechavi M. Convergent evolution of venom gland transcriptomes across Metazoa. PNAS. 2022;119:e2111392119.

93. Perry BW, Schield DR, Westfall AK, Mackessy SP, Castoe TA. Physiological demands and signaling associated with snake venom production and storage illustrated by transcriptional analyses of venom glands. Scientific Reports. 2020;10:18083.

94. Haney RA, Ayoub NA, Clarke TH, Hayashi CY, Garb JE. Dramatic expansion of the black widow toxin arsenal uncovered by multi-tissue transcriptomics and venom proteomics. BMC Genomics. 2014;15:366.

95. Bray NL, Pimentel H, Melsted P, Pachter L. Near-optimal probabilistic RNA-seq quantification. Nat Biotechnol. 2016;34:525–7.

96. Robinson MD, McCarthy DJ, Smyth GK. edgeR: a Bioconductor package for differential expression analysis of digital gene expression data. Bioinformatics. 2010;26:139–40.

97. Chen C, Hou J, Tanner JJ, Cheng J. Bioinformatics methods for mass spectrometry-based proteomics data analysis. International Journal of Molecular Sciences. 2020;21:2873.

98. Perkins DN, Pappin DJC, Creasy DM, Cottrell JS. Probability-based protein identification by searching sequence databases using mass spectrometry data. Electrophoresis. 1999;20:3551–67.

99. Zhang J, Xin L, Shan B, Chen W, Xie M, Yuen D, et al. PEAKS DB: De novo sequencing assisted database search for sensitive and accurate peptide identification. Molecular & Cellular Proteomics. 2012;11:M111.010587.

100. Orsburn BC. Proteome discoverer—A community enhanced data processing suite for protein informatics. Proteomes. 2021;9:15.

101. Eng JK, McCormack AL, Yates JR. An approach to correlate tandem mass spectral data of peptides with amino acid sequences in a protein database. J Am Soc Mass Spectrom. 1994;5:976–89.

102. Bern M, Kil YJ, Becker C. Byonic: Advanced peptide and protein identification software. Curr Protoc Bioinformatics. 2012;40:13.20.1-13.20.14.

103. Chi H, Liu C, Yang H, Zeng W-F, Wu L, Zhou W-J, et al. Comprehensive identification of peptides in tandem mass spectra using an efficient open search engine. Nat Biotechnol. 2018;36:1059–61.

104. Kong AT, Leprevost FV, Avtonomov DM, Mellacheruvu D, Nesvizhskii AI. MSFragger: Ultrafast and comprehensive peptide identification in mass spectrometry–based proteomics. Nat Methods. 2017;14:513–20.

105. Vaudel M, Burkhart JM, Zahedi RP, Oveland E, Berven FS, Sickmann A, et al. PeptideShaker enables reanalysis of MS-derived proteomics data sets. Nat Biotechnol. 2015;33:22–4.

106. Cox J, Mann M. MaxQuant enables high peptide identification rates, individualized p.p.b.-range mass accuracies and proteome-wide protein quantification. Nat Biotechnol. 2008;26:1367–72.

107. MacLean B, Tomazela DM, Shulman N, Chambers M, Finney GL, Frewen B, et al. Skyline: an open source document editor for creating and analyzing targeted proteomics experiments. Bioinformatics. 2010;26:966–8.

108. Demichev V, Messner CB, Vernardis SI, Lilley KS, Ralser M. DIA-NN: neural networks and interference correction enable deep proteome coverage in high throughput. Nat Methods. 2020;17:41–4.

109. Sinitcyn P, Hamzeiy H, Salinas Soto F, Itzhak D, McCarthy F, Wichmann C, et al. MaxDIA enables library-based and library-free data-independent acquisition proteomics. Nat Biotechnol. 2021;39:1563–73.

110. Doerr A. DIA mass spectrometry. Nat Methods. 2015;12:35–35.

111. Ma B. Novor: Real-time peptide de novo sequencing software. J Am Soc Mass Spectrom. 2015;26:1885–94.

112. Yang H, Chi H, Zeng W-F, Zhou W-J, He S-M. pNovo 3: precise de novo peptide sequencing using a learning-to-rank framework. Bioinformatics. 2019;35:i183–90.

113. Melani RD, Nogueira FCS, Domont GB. It is time for top-down venomics. Journal of Venomous Animals and Toxins including Tropical Diseases. 2017;23:44.

114. Röst HL, Sachsenberg T, Aiche S, Bielow C, Weisser H, Aicheler F, et al. OpenMS: a flexible open-source software platform for mass spectrometry data analysis. Nat Methods. 2016;13:741–8.

115. Schmid R, Heuckeroth S, Korf A, Smirnov A, Myers O, Dyrlund TS, et al. Integrative analysis of multimodal mass spectrometry data in MZmine 3. Nat Biotechnol. 2023;41:447–9.

116. Liu X, Inbar Y, Dorrestein PC, Wynne C, Edwards N, Souda P, et al. Deconvolution and database search of complex tandem mass spectra of intact proteins. Molecular & Cellular Proteomics. 2010;9:2772–82.

117. Adusumilli R, Mallick P. Data Conversion with ProteoWizard msConvert. In: Comai L, Katz JE, Mallick P, editors. Proteomics: Methods and Protocols. New York: Springer US; 2017. p. 339–68.

118. Liu X, Sirotkin Y, Shen Y, Anderson G, Tsai YS, Ting YS, et al. Protein identification using top-down spectra. Molecular & Cellular Proteomics. 2012;11:M111.008524.

119. Guner H, Close PL, Cai W, Zhang H, Peng Y, Gregorich ZR, et al. MASH Suite: A user-friendly and versatile software interface for high-resolution mass spectrometry data interpretation and visualization. J Am Soc Mass Spectrom. 2014;25:464–70.

120. Sun R-X, Luo L, Wu L, Wang R-M, Zeng W-F, Chi H, et al. pTop 1.0: A high-accuracy and high-efficiency search engine for intact protein identification. Anal Chem. 2016;88:3082–90.

121. Kou Q, Wu S, Tolić N, Paša-Tolić L, Liu Y, Liu X. A mass graph-based approach for the identification of modified proteoforms using top-down tandem mass spectra. Bioinformatics. 2017;33:1309–16.

122. Kou Q, Xun L, Liu X. TopPIC: a software tool for top-down mass spectrometry-based proteoform identification and characterization. Bioinformatics. 2016;32:3495–7.

123. Park J, Piehowski PD, Wilkins C, Zhou M, Mendoza J, Fujimoto GM, et al. Informed-Proteomics: open-source software package for top-down proteomics. Nat Methods. 2017;14:909–14.

124. Gupta S, Kapoor P, Chaudhary K, Gautam A, Kumar R, Consortium OSDD, et al. *In silico* approach for predicting toxicity of peptides and proteins. PLOS ONE. 2013;8:e73957.

125. Sharma N, Naorem LD, Jain S, Raghava GPS. ToxinPred2: an improved method for predicting toxicity of proteins. Brief Bioinform. 2022;23:bbac174.

126. Mooney C, Haslam NJ, Pollastri G, Shields DC. Towards the improved discovery and design of functional peptides: Common features of diverse classes permit generalized prediction of bioactivity. PLOS ONE. 2012;7:e45012.

127. Lamiable A, Thévenet P, Rey J, Vavrusa M, Derreumaux P, Tufféry P. PEP-FOLD3: faster de novo structure prediction for linear peptides in solution and in complex. Nucleic Acids Research. 2016;44:W449–54.

128. Rey J, Murail S, de Vries S, Derreumaux P, Tuffery P. PEP-FOLD4: a pH-dependent force field for peptide structure prediction in aqueous solution. Nucleic Acids Res. 2023;51:W432–7.

129. Mirdita M, Schütze K, Moriwaki Y, Heo L, Ovchinnikov S, Steinegger M. ColabFold: making protein folding accessible to all. Nat Methods. 2022;19:679–82.

130. Baek M, DiMaio F, Anishchenko I, Dauparas J, Ovchinnikov S, Lee GR, et al. Accurate prediction of protein structures and interactions using a three-track neural network. Science. 2021;373:871–6.

131. Källberg M, Wang H, Wang S, Peng J, Wang Z, Lu H, et al. Template-based protein structure modeling using the RaptorX web server. Nat Protoc. 2012;7:1511–22.

132. Choi M, Carver J, Chiva C, Tzouros M, Huang T, Tsai T-H, et al. MassIVE.quant: A community resource of quantitative mass spectrometry-based proteomics datasets. Nat Methods. 2020;17:981–4.

133. Wishart DS, Tzur D, Knox C, Eisner R, Guo AC, Young N, et al. HMDB: the Human Metabolome Database. Nucleic Acids Res. 2007;35:D521-526.

134. Alonso LL, Slagboom J, Casewell NR, Samanipour S, Kool J. Metabolome-based classification of snake venoms by bioinformatic tools. Toxins (Basel). 2023;15:161.

135. Wang M, Carver JJ, Phelan VV, Sanchez LM, Garg N, Peng Y, et al. Sharing and community curation of mass spectrometry data with Global Natural Products Social Molecular Networking. Nat Biotechnol. 2016;34:828–37.

136. Fischer T, Riedl R. Paracelsus' legacy in the faunal realm: Drugs deriving from animal toxins. Drug Discovery Today. 2022;27:567–75.

137. Crnković A, Srnko M, Anderluh G. Biological nanopores: Engineering on demand. Life. 2021;11:27.

138. Tyagi A, Tuknait A, Anand P, Gupta S, Sharma M, Mathur D, et al. CancerPPD: a database of anticancer peptides and proteins. Nucleic Acids Research. 2015;43:D837–43.

139. di Micco P, Antolin AA, Mitsopoulos C, Villasclaras-Fernandez E, Sanfelice D, Dolciami D, et al. canSAR: update to the cancer translational research and drug discovery knowledgebase. Nucleic Acids Research. 2023;51:D1212–9.

140. Faraji N, Arab SS, Doustmohammadi A, Daly NL, Khosroushahi AY. ApInAPDB: a database of apoptosis-inducing anticancer peptides. Sci Rep. 2022;12:21341.

141. Cadow J, Born J, Manica M, Oskooei A, Rodríguez Martínez M. PaccMann: a web service for interpretable anticancer compound sensitivity prediction. Nucleic Acids Research. 2020;48:W502–8.

142. Petrov I, Alexeyenko A. EviCor: Interactive web platform for exploration of molecular features and response to anti-cancer drugs. J Mol Biol. 2022;434:167528.

143. Romano JD, Tatonetti NP. VenomKB, a new knowledge base for facilitating the validation of putative venom therapies. Sci Data. 2015;2:150065.

144. SIB Swiss Institute of Bioinformatics RDF Group Members. The SIB Swiss Institute of Bioinformatics Semantic Web of data. Nucleic Acids Research. 2023;gkad902.

145. Sima AC, Mendes de Farias T, Zbinden E, Anisimova M, Gil M, Stockinger H, et al. Enabling semantic queries across federated bioinformatics databases. Database. 2019;2019:baz106.

146. Galgonek J, Vondrášek J. IDSM ChemWebRDF: SPARQLing small-molecule datasets. Journal of Cheminformatics. 2021;13:38.

147. Groza T, Gomez FL, Mashhadi HH, Muñoz-Fuentes V, Gunes O, Wilson R, et al. The International Mouse Phenotyping Consortium: Comprehensive knockout phenotyping underpinning the study of human disease. Nucleic Acids Research. 2023;51:D1038–45.

148. Howe DG, Bradford YM, Eagle A, Fashena D, Frazer K, Kalita P, et al. The Zebrafish Model Organism Database: New support for human disease models, mutation details, gene expression phenotypes and searching. Nucleic Acids Res. 2017;45:D758–68.

149. Gramates LS, Agapite J, Attrill H, Calvi BR, Crosby MA, dos Santos G, et al. FlyBase: A guided tour of highlighted features. Genetics. 2022;220:iyac035.

150. Grosdidier A, Zoete V, Michielin O. SwissDock, a protein-small molecule docking web service based on EADock DSS. Nucleic Acids Res. 2011;39:W270-277.

151. Romero-Molina S, Ruiz-Blanco YB, Mieres-Perez J, Harms M, Münch J, Ehrmann M, et al. PPI-Affinity: A web tool for the prediction and optimization of protein–peptide and protein–protein binding affinity. J Proteome Res. 2022;21:1829–41.

152. Lei Y, Li S, Liu Z, Wan F, Tian T, Li S, et al. A deep-learning framework for multi-level peptide–protein interaction prediction. Nat Commun. 2021;12:5465.

153. Vidal-Limon A, Aguilar-Toalá JE, Liceaga AM. Integration of molecular docking analysis and molecular dynamics simulations for studying food proteins and bioactive peptides. J Agric Food Chem. 2022;70:934–43.

154. Almeida JR, Palacios ALV, Patiño RSP, Mendes B, Teixeira CAS, Gomes P, et al. Harnessing snake venom phospholipases A2 to novel approaches for overcoming antibiotic resistance. Drug Development Research. 2019;80:68–85.

155. Clark GC, Casewell NR, Elliott CT, Harvey AL, Jamieson AG, Strong PN, et al. Friends or foes? Emerging impacts of biological toxins. Trends in Biochemical Sciences. 2019;44:365–79.

156. Holmes DE. The data explosion. In: Holmes DE, editor. Big Data: A very short introduction. Oxford: Oxford Academic; 2017. online edn.

157. Ong E, Xiang Z, Zhao B, Liu Y, Lin Y, Zheng J, et al. Ontobee: A linked ontology data server to support ontology term dereferencing, linkage, query and integration. Nucleic Acids Research. 2017;45:D347–52.

158. Emam M, Tarek A, Soudy M, Antunes A, Hadidi ME, Hamed M. Comparative evaluation of multiomics integration tools for the study of prediabetes: insights into the earliest stages of type 2 diabetes mellitus. Netw Model Anal Health Inform Bioinforma. 2024;13:8.

159. Wilkinson MD, Dumontier M, Aalbersberg IjJ, Appleton G, Axton M, Baak A, et al. The FAIR Guiding Principles for scientific data management and stewardship. Sci Data. 2016;3:160018.

160. Whetzel PL, Noy NF, Shah NH, Alexander PR, Nyulas C, Tudorache T, et al. BioPortal: enhanced functionality via new web services from the National Center for Biomedical Ontology to access and use ontologies in software applications. Nucleic Acids Res. 2011;39:W541-545.

161. European Commission, Directorate-General for Research and Innovation. Turning FAIR into reality – Final report and action plan from the European Commission expert group on FAIR data. Publications Office; 2018. https://data.europa.eu/doi/10.2777/1524

Figure legend

Figure 1. Specialised and generalist web resources, databases and tools used in venom research. In a typical venom research workflow, raw data from venoms or venom glands are deposited in primary databases and information is generally subsequently stored in secondary and specialised databases. Such information can be accessed and analysed using different tools for a variety of research purposes. Dbs = databases.

Additional files

Additional file 1.pdf: Survey on web resources in venom research. Questions included in the survey sent to the members of the EUVEN COST Action and the participants of the 1st International EUVEN Congress in 2021.

Additional file 2.csv: Answers to the survey. Anonymised answers to the survey.

Additional file 3.tsv: Summary of venom research areas. Contingency table of the research areas represented by the respondents of the survey used to create Figure S1.

Additional file 4.tsv: Summary of organisms studied in venom research. Contingency table of the organisms studied by the respondents of the survey used to create Figure S2.

Figure 1

Click here to access/download;Figure;Figure1.jpg

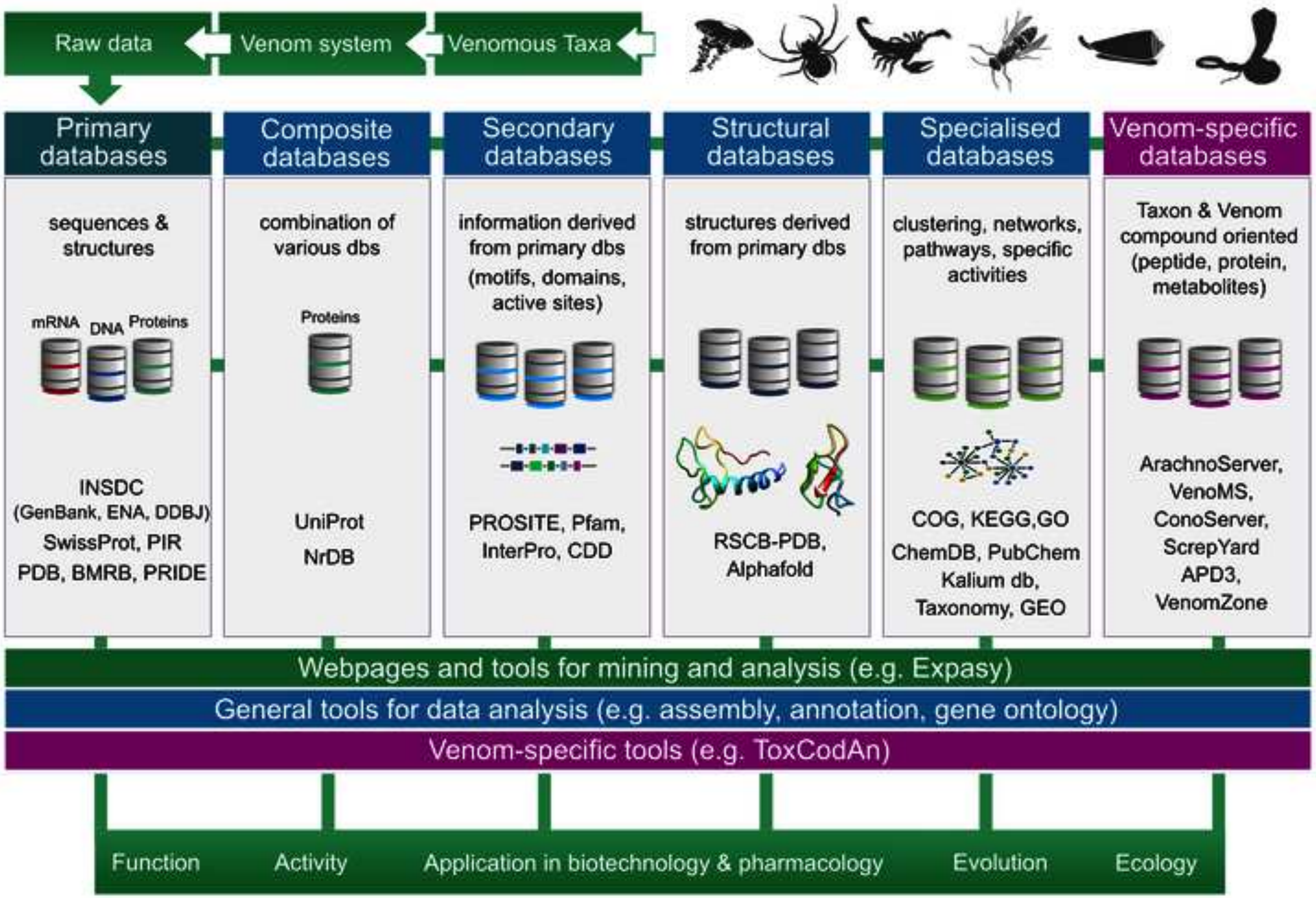

Click here to access/download
Supplementary Material
Zancolli_et_al_Supplementary_Information_submission.pdf

Additional file 1

Click here to access/download
Supplementary Material
Additional file 1.pdf

Additional file 2

Click here to access/download
Supplementary Material
Additional file 2.csv

Additional file 3

Click here to access/download
Supplementary Material
Additional file 3.tsv

Additional file 4

Click here to access/download

Supplementary Material

Additional file 4.tsv
