# Supplementary material for: Web of venom: exploration of big data resources in animal toxin research
Source: Gigascience. 2024 Sep 9;13:giae054. doi: 10.1093/gigascience/giae054 (PMC11382406; doi:10.1093/gigascience/giae054)
Supplement: giae054_Supplemental_Files [file giae054_supplemental_files.zip › Additional file 1.pdf]

WG4 - Web Resources Survey

European Venom Network (EUVEN) – Web resource survey
This questionnaire was created within the framework of the working group on web resources (WG4). It aims to identify the needs and difficulties of scientists working on venoms. It is strictly limited to EUVEN COST action members. Depending on the answers, and to the possible extent, it could give a direction of what to list and implement in the future in terms of new tools, protocols, etc.

giulia.zancolli@gmail.com Switch accounts
Not shared

* Indicates required question

What is your name and Institute/Lab? *

Your answer

In which venomics area do you work? *

- [ ] Genomics
- [ ] Transcriptomics
- [ ] Proteomics
- [ ] Metabolomics
- [ ] Functional morphology and spatial omics
- [ ] Toxin structure and structure prediction
- [ ] Toxin function
- [ ] Clinical toxinology
- [ ] Antivenom
- [ ] Evolution
- [ ] Phylogenetics
- [ ] Quantitative genetics
- [ ] Other:

Which organisms do you mainly work on? *

- [ ] Sea anemones
- [ ] Scorpions
- [ ] Spiders
- [ ] Insects
- [ ] Cone snails
- [ ] Other invertebrates (please specify which taxon in 'Other')
- [ ] Fishes
- [ ] Amphibians
- [ ] Snakes
- [ ] Other reptiles (please specify which taxon in 'Other')
- [ ] Mammals
- [ ] No taxon specific
- [ ] Other:

Which database do you regularly use? *

- [ ] VenomZone
- [ ] ArachnoServer
- [ ] ConoServer
- [ ] European Scorpion database (https://www.ntnu.no/ub/scorpion-files/european_scorp.php)
- [ ] Kalium DB
- [ ] UniProtKB/Swiss-Prot (Tox-Prot program dedicated to toxins)
- [ ] UniProtKB in general (Swiss-Prot and TrEMBL) for toxins and targets
- [ ] WHO (https://apps.who.int/bloodproducts/snakeantivenoms/database/default.htm)
- [ ] Ensembl
- [ ] NCBI Genbank
- [ ] Other:

Do you use VenomZone and which pages you are interested in? *

- [ ] I don't use VenomZone
- [ ] Venom composition page per species (e.g. https://venomzone.expasy.org/5277)
- [ ] Toxin targets found in each taxonomic group (e.g. https://venomzone.expasy.org/1217)
- [ ] Nomenclature page (e.g. https://venomzone.expasy.org/1877)
- [ ] Protein families in taxonomic groups (e.g. https://venomzone.expasy.org/2256)
- [ ] Do you have any suggestions for improving VenomZone (please answer in 'Other')?
- [ ] Other:

What kind of web resources do you regularly use? *

- [ ] Database
- [ ] Repository
- [ ] Bioinformatic tools (alignment, translation, etc. e.g. Blast)
- [ ] Multi-tool resource portal (e.g. Expasy, Galaxy)
- [ ] Tools for prediction and modelling (e.g. SWISS-MODEL, InterPro)
- [ ] Other:

Which softwares do you regularly use?

Your answer

Which analytic tools would you like to see developed? *

Your answer

Which difficulties do you encounter when using bioinformatics tools?

Your answer

Do you have any suggestions for improvement of the currently available web resources?

Your answer

Submit

Clear form

Never submit passwords through Google Forms.

This content is neither created nor endorsed by Google. Report Abuse - Terms of Service - Privacy Policy

Google Forms
