# Supplementary material for: Web of venom: exploration of big data resources in animal toxin research
Source: Gigascience. 2024 Sep 9;13:giae054. doi: 10.1093/gigascience/giae054 (PMC11382406; doi:10.1093/gigascience/giae054)
Supplement: giae054_Supplemental_Files [file giae054_supplemental_files.zip › Additional file 5.pdf]

Additional results

General overview of the venom research community in Europe: research areas, target organisms, and resources

A survey was initiated in March 2021 and targeted the members of the European Venom Network (EUVEN) COST Action CA19144, and the participants of the 1st International EUVEN Congress, a virtual event held from 14th to 16th September 2021. Following the congress, a total of 116 participants responded to the survey.

The landscape of venom research of the survey participants, mostly Europe-based, exhibits remarkable diversity, devoid of pronounced biases towards specific research domains (Fig. S1). Within this spectrum, researchers investigating the molecular targets of toxins and their functional implications constituted 21% of the respondents. An additional 18% were engaged in proteomic analyses to decipher venom composition, while 10% focused on toxin gene expression. Conversely, areas of more specialised focus, such as the exploration of microbial components and their translational applications in disease treatments, or anticancer investigations, remained relatively underrepresented, each accounting for only 1% of respondents.

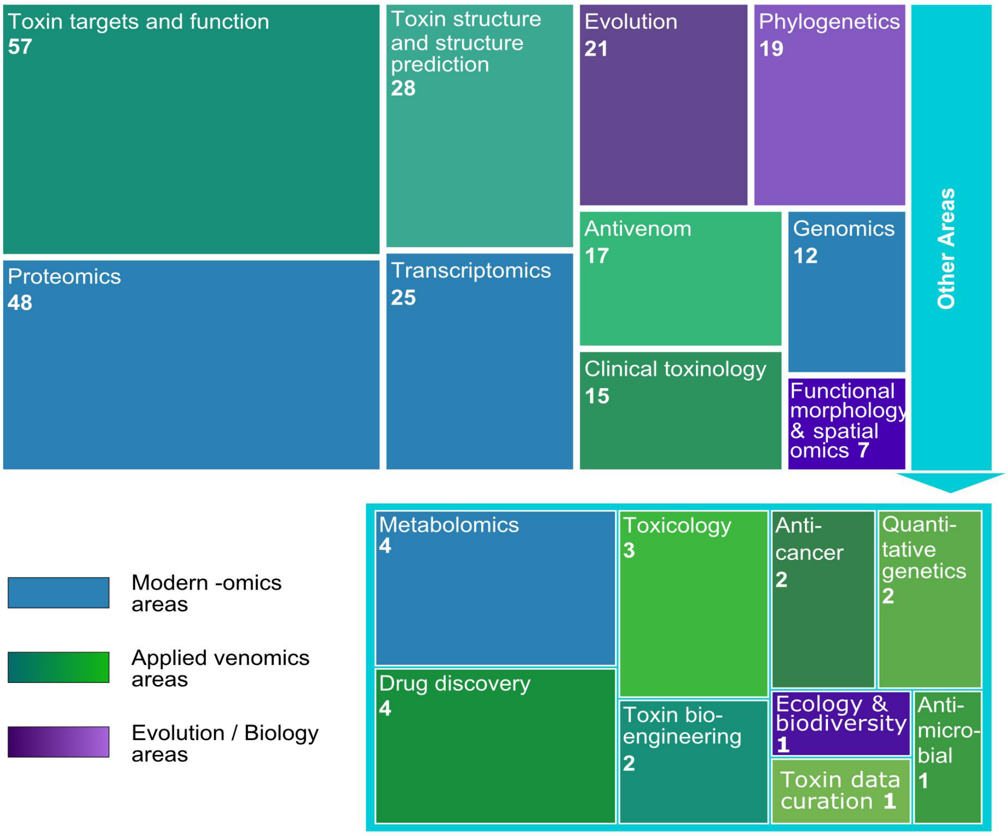

Figure S1. Represented research domains in venom research. The size of rectangles reflects the percentage, and the absolute numbers are indicated. The colour code indicates the major fields in venom research.

Among the animals subject to venom research, snakes were the most common (23%), followed by insects at 14%, scorpions, spiders, and cone snails at 9% (Fig. S2). A minority of participants were engaged in studying fungi, bacteria, and viruses, thereby broadening the scope of the EUVEN network beyond animal venoms.

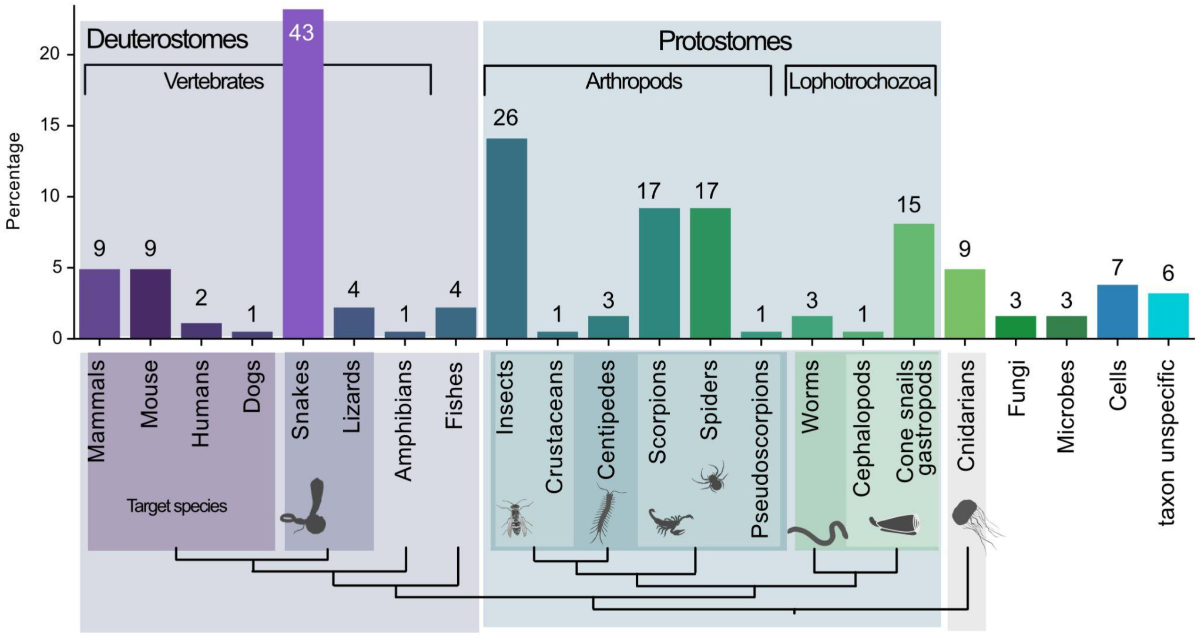

Figure S2. Identified species that are subject of venom research. The taxa are arranged according to the phylogeny of the animal kingdom. The mammal representatives and cells are rather utilised as target organisms to study, for instance, the effects of venom injection or toxin activity, rather than sources of venom. The bars represent the percentage among all respondents, and the absolute values are given on top.

Among the web resources used by venom researchers, protein and nucleotide sequences databases are the most common, with UniProtKB and NCBI Genbank on top of the rank with over 50% of the responders using them (Fig. S3). Among the venom-specialised resource, Tox-Prot and VenomZone were the most common, followed by ConoServer and ArachnoServer.

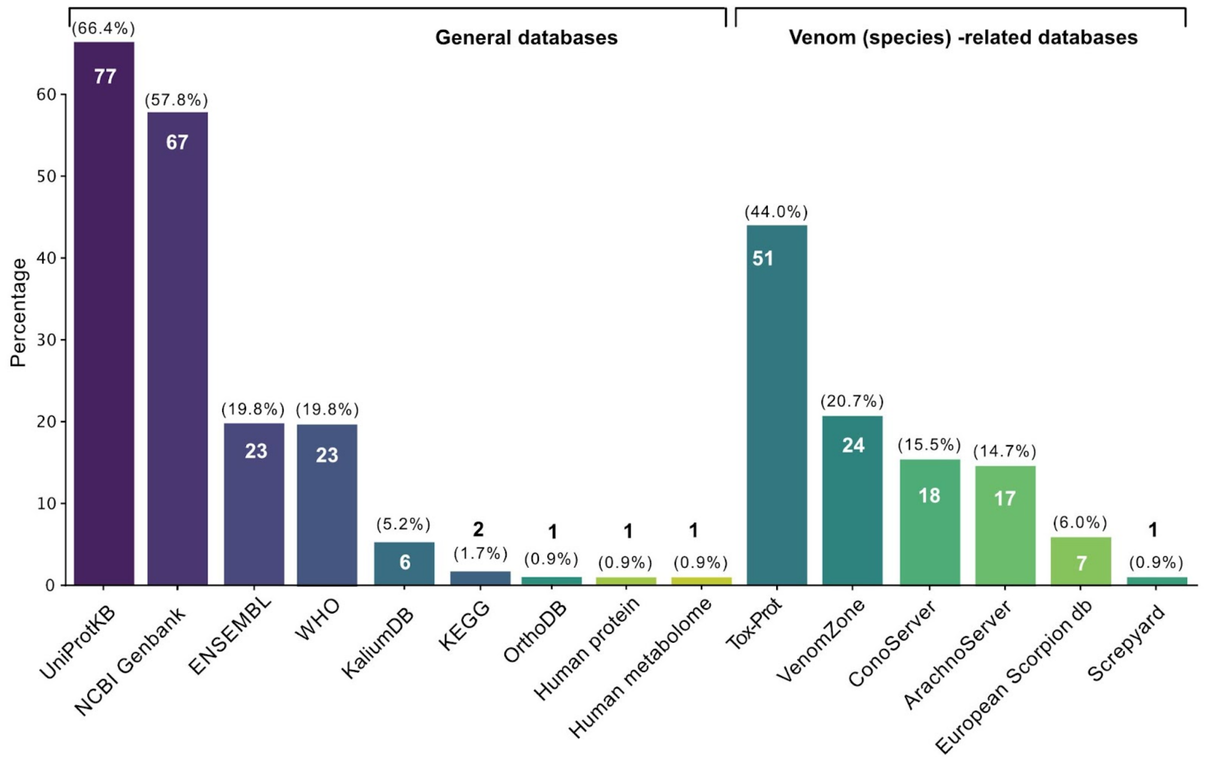

Figure S3. Overview of the most widely used web resources. On the y-axis the percentage of responders is reported per database, while the absolute numbers are given on top of the bars.

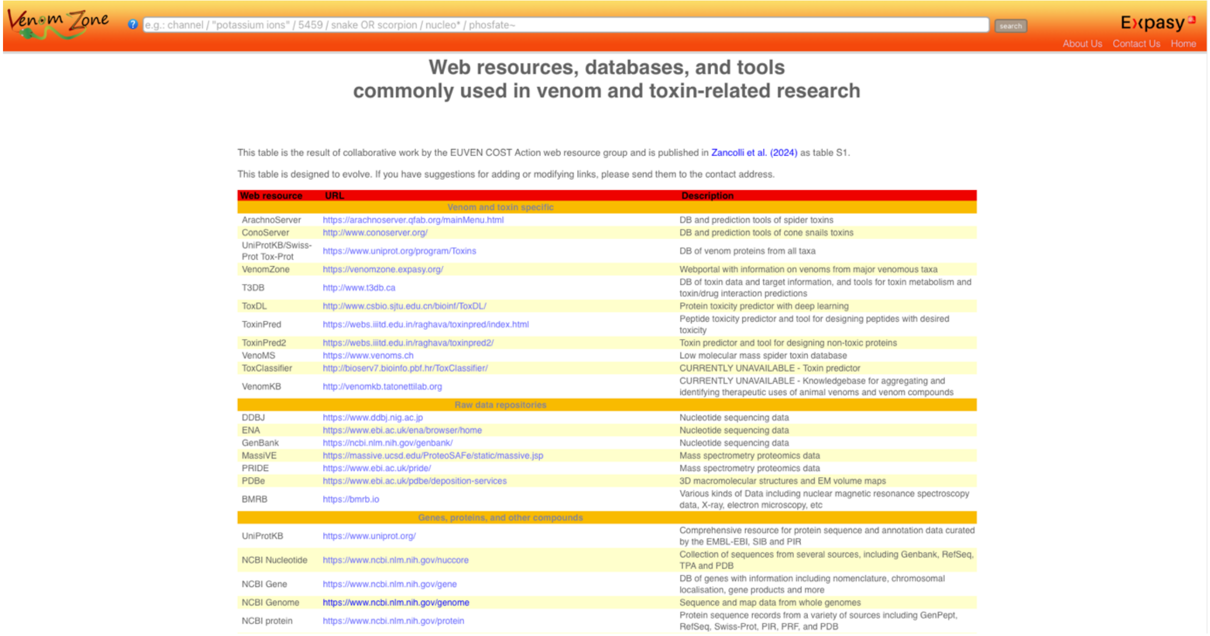

VenomZone | e.g.: channel / "potassium ions" / 5459 / snake OR scorpion / nucleo* / phosfate~ | search | Expasy
About Us | Contact Us | Home

Web resources, databases, and tools commonly used in venom and toxin-related research

This table is the result of collaborative work by the EUVEN COST Action web resource group and is published in Zancolli et al. (2024) as table S1.

This table is designed to evolve. If you have suggestions for adding or modifying links, please send them to the contact address.

Web resource	URL	Description
**Venom and toxin specific**		
ArachnoServer	https://arachnoserver.qfab.org/mainMenu.html	DB and prediction tools of spider toxins
ConoServer	http://www.conoserver.org/	DB and prediction tools of cone snails toxins
UniProtKB/Swiss-Prot Tox-Prot	https://www.uniprot.org/program/Toxins	DB of venom proteins from all taxa
VenomZone	https://venomzone.expasy.org/	Webportal with information on venoms from major venomous taxa
T3DB	http://www.t3db.ca	DB of toxin data and target information, and tools for toxin metabolism and toxin/drug interaction predictions
ToxDL	http://www.csbio.sjtu.edu.cn/bioinf/ToxDL/	Protein toxicity predictor with deep learning
ToxinPred	https://webs.iiitd.edu.in/raghava/toxinpred/index.html	Peptide toxicity predictor and tool for designing peptides with desired toxicity
ToxinPred2	https://webs.iiitd.edu.in/raghava/toxinpred2/	Toxin predictor and tool for designing non-toxic proteins
VenoMS	https://www.venoms.ch	Low molecular mass spider toxin database
ToxClassifier	http://bioserv7.bioinfo.pbf.hr/ToxClassifier/	CURRENTLY UNAVAILABLE - Toxin predictor
VenomKB	http://venomkb.tatonettilab.org	CURRENTLY UNAVAILABLE - Knowledgebase for aggregating and identifying therapeutic uses of animal venoms and venom compounds
**Raw data repositories**		
DDBJ	https://www.ddbj.nig.ac.jp	Nucleotide sequencing data
ENA	https://www.ebi.ac.uk/ena/browser/home	Nucleotide sequencing data
GenBank	https://ncbi.nlm.nih.gov/genbank/	Nucleotide sequencing data
MassIVE	https://massive.ucsd.edu/ProteoSAFe/static/massive.jsp	Mass spectrometry proteomics data
PRIDE	https://www.ebi.ac.uk/pride/	Mass spectrometry proteomics data
PDBe	https://www.ebi.ac.uk/pdbe/deposition-services	3D macromolecular structures and EM volume maps
BMRB	https://bmrb.io	Various kinds of Data including nuclear magnetic resonance spectroscopy data, X-ray, electron microscopy, etc
**Genes, proteins, and other compounds**		
UniProtKB	https://www.uniprot.org/	Comprehensive resource for protein sequence and annotation data curated by the EMBL-EBI, SIB and PIR
NCBI Nucleotide	https://www.ncbi.nlm.nih.gov/nuccore	Collection of sequences from several sources, including Genbank, RefSeq, TPA and PDB
NCBI Gene	https://www.ncbi.nlm.nih.gov/gene	DB of genes with information including nomenclature, chromosomal localisation, gene products and more
NCBI Genome	https://www.ncbi.nlm.nih.gov/genome	Sequence and map data from whole genomes
NCBI protein	https://www.ncbi.nlm.nih.gov/protein	Protein sequence records from a variety of sources including GenPept, RefSeq, Swiss-Prot, PIR, PRF, and PDB

Figure S4. Screenshot of the VenomZone webpage listing the compiled web resources utilised in venom research. This webpage currently lists all the web resources, databases and tools gathered within the framework of this paper. New relevant databases or online tools, as well as changes in the already compiled resources, can be added by contacting the VenomZone curator using the “Contact Us” link on the top-right.

Table S1. List of web resources and online tools utilised in venom research.

	Web resource	URL	Description
**Venom and toxin specific**	ArachnoServer	https://arachnoserver.qfab.org/mainMenu.html	DB and prediction tools of spider toxins
	ConoServer	http://www.conoserver.org/	DB and prediction tools of cone snails toxins
	UniProtKB/Swiss-Prot Tox-Prot	https://www.uniprot.org/program/Toxins	DB of venom proteins from all taxa
	VenomZone	https://venomzone.expasy.org/	Webportal with information on venoms from major venomous taxa
	T3DB	http://www.t3db.ca	DB of toxin data and target information, and tools for toxin metabolism and toxin/drug interaction predictions
	ToxDL	http://www.csbio.sjtu.edu.cn/bioinf/ToxDL/	Protein toxicity predictor with deep learning
	ToxinPred	https://webs.iiitd.edu.in/raghava/toxinpred/index.html	Peptide toxicity predictor and tool for designing peptides with desired toxicity
	ToxinPred2	https://webs.iiitd.edu.in/raghava/toxinpred2/	Toxin predictor and tool for designing non-toxic proteins
	VenoMS	https://www.venoms.ch	Low molecular mass spider toxin database
	ToxClassifier	http://bioserv7.bioinfo.pbf.hr/ToxClassifier/	CURRENTLY UNAVAILABLE - Toxin predictor
	VenomKB	http://venomkb.tatonettilab.org	CURRENTLY UNAVAILABLE - Knowledgebase for aggregating and identifying therapeutic uses of animal venoms and venom compounds
**Raw data repositories**	DDBJ	https://www.ddbj.nig.ac.jp	Nucleotide sequencing data
	ENA	https://www.ebi.ac.uk/ena/browser/home	Nucleotide sequencing data
	GenBank	https://ncbi.nlm.nih.gov/genbank/	Nucleotide sequencing data
	MassiVE	https://massive.ucsd.edu/ProteoSAFe/static/massive.jsp	Mass spectrometry proteomics data
	PRIDE	https://www.ebi.ac.uk/pride/	Mass spectrometry proteomics data
	PASSEL	https://peptideatlas.org/passel/	Selected reaction monitoring (SRM/MRM) data
	PDBe	https://www.ebi.ac.uk/pdbe/deposition-services	3D macromolecular structures and EM volume maps
	BMRB	https://bmrb.io	Various kinds of data including nuclear magnetic resonance spectroscopy data, X-ray, electron microscopy, etc
	EMDB	https://www.ebi.ac.uk/emdb/	Repository for cryogenic-sample Electron Microscopy (cryoEM) volumes and representative tomograms of macromolecular complexes and subcellular structures
**Genes, proteins, and other compounds**	UniProtKB	https://www.uniprot.org/	Comprehensive resource for protein sequence and annotation data curated by the EMBL-EBI, SIB and PIR
	NCBI Nucleotide	https://www.ncbi.nlm.nih.gov/nuccore	Collection of sequences from several sources, including Genbank, RefSeq, TPA and PDB
	NCBI Gene	https://www.ncbi.nlm.nih.gov/gene	DB of genes with information including nomenclature, chromosomal localisation, gene products and more
	NCBI Genome	https://www.ncbi.nlm.nih.gov/genome	Sequence and map data from whole genomes
	NCBI protein	https://www.ncbi.nlm.nih.gov/protein	Protein sequence records from a variety of sources including GenPept, RefSeq, Swiss-Prot, PIR, PRF, and PDB

	NCBI PubChem	https://pubchem.ncbi.nlm.nih.gov	DB containing chemical information on a variety of compounds
	NCBI TSA	https://www.ncbi.nlm.nih.gov/Traces/wgs/?view=TSA	DB of computationally assembled transcript sequences from primary data such as ESTs and RNA-Seq technologies
	Ensembl	https://www.ensembl.org/	Genome browser for vertebrate genomes with tools for comparative analysis and predictions
	Ensembl Metazoa	http://metazoa.ensembl.org/index.html	Gene and genome data for Metazoan species
	Ensembl Genome	http://ensemblgenomes.org	Genome data for non-vertebrate species with tools for manipulation, analysis and visualisation
	GenomeArk	https://www.genomeark.org	DB of genome sequencing and assembly data generated by the Earth BioGenome Project, the Vertebrate Genomes Project, the Telomere-to-Telomere Consortium, and other related projects.
	PeptideAtlas	https://peptideatlas.org	Multiorganism compendium of peptides identified from mass spectrometry data
	Kalium DB	https://kaliumdb.org/	DB of polypeptidic ligands of potassium channels
	ScrepYard	https://screpyard.org	DB of secreted cysteine-rich repeat peptides
	APD3	https://aps.unmc.edu	Antimicrobial peptide DB
**3d structure and peptide prediction**	RCSB-PDB	https://www.rcsb.org/	Archive of 3D structure data for large biological molecules curated by the US data center for the global protein data bank (PDB)
	PDBe	https://www.ebi.ac.uk/pdbe/	Archive data on biological macromolecular structures curated by EMBL-EBI
	BMRB	https://bmrb.io	Archive spectral and quantitative data derived from nuclear magnetic resonance
	AlphaFold Protein Structure Database	https://alphafold.ebi.ac.uk/	DB of protein structure predictions based on an AI system developed by GoogleDeepMind
	AlphaFold2	https://colab.research.google.com/github/sokrypton/ColabFold/blob/main/AlphaFold2.ipynb	Server for the prediction of 3D structure form amino acid sequences
	RoseTTAFold	https://robetta.bakerlab.org/	Protein structure and interaction prediction server including RoseTTAFold and TrRosetta
	KNOTTIN	https://www.dsimb.inserm.fr/KNOTTIN/index.php	DB and prediction tools of small disulfide-rich proteins characterised by cysteine knots
	Peptide Ranker	http://distilldeep.ucd.ie/PeptideRanker/	Server for the prediction of bioactive peptides based on a neural network
	PEP-FOLD3	https://bioserv.rpbs.univ-paris-diderot.fr/services/PEP-FOLD3/	Tool for prediction of peptide structure from amino acid sequences
	PEP-FOLD4	http://bioserv.rpbs.univ-paris-diderot.fr/services/PEP-FOLD4	Improved version of PEP-FOLD3 that considers pH and ionic strength variation
**Gene function, interactions and networks**	Gene ontology (GO) knowledgebase	http://geneontology.org/	Human- and machine-readable source of information on the functions of genes
	STRING	https://string-db.org/	DB of protein associations, including both physical interactions and functional associations
	KEGG	https://www.genome.jp/kegg/	DB for understanding high-level functions and utilities of the biological system, such as the cell, the organism and the ecosystem, from genomic and molecular-level information
	IntAct	https://www.ebi.ac.uk/intact/home	DB and analysis tools for molecular interaction data
	Bgee	https://www.bgee.org	DB for retrieval and comparison of gene expression patterns across animal species
	NCBI GEO	https://www.ncbi.nlm.nih.gov/geo/	Gene expression repository from array- and sequencing-based data

	Pannzer2	http://ekhidna2.biocenter.helsinki.fi/sanspanz/	Fully automated service for functional annotation of proteins of unknown function
	Philius	https://www.yeastrc.org/philius/pages/philius/runPhilius.jsp	Transmembrane prediction server
	SignalP	https://services.healthtech.dtu.dk/services/SignalP-6.0/	Prediction of signal peptides and their cleavage sites from amino acid sequences
	Phobius	https://phobius.sbc.su.se	Combined transmembrane topology and signal peptide predictor tool
	WoLF PSORT	https://wolfpsort.hgc.jp	Protein subcellular localisation prediction
	The Eukaryotic Linear Motif resource for	http://elm.eu.org/	Resource for annotation and detection of Eukaryotic Linear Motifs (ELM) or Short Linear Motifs (SLiMs) of adjacent amino acids
	Rhea - reaction knowledgebase	https://www.rhea-db.org	DB for chemical and transport reactions
	WashU Epigenome Browser	https://epigenomegateway.wustl.edu/browser/	Genomics data exploration tool for the visualisation, integration adn analysis of epigenomic datasets
**Gene families and classification**	Pfam	http://pfam.xfam.org/	Large collection of protein families, each represented by multiple sequence alignments and hidden Markov models (HMMs)
	Interpro	http://www.ebi.ac.uk/interpro/	Resource providing functional analysis of proteins by classifying them into families and predicting domains and important sites
	PANTHER	https://www.pantherdb.org	Comprehensive information about the evolution of protein-coding gene families
	Quest for Orthologs	https://questfororthologs.org/orthology_databases	List of orthology databases
	OrthoDB	https://www.orthodb.org/	Hierarchical catalog of orthologs - mapping genomics to functional data
	OMA	https://omabrowser.org/oma/home/	DB and method for the inference of orthologs among complete genomes
	NCBI COG	https://www.ncbi.nlm.nih.gov/research/cog	DB of clusters of orthologous genes
	PhylomeDB	http://phylomedb.org	DB of catalogues of gene phylogenies with phylogenetic trees, orthology predictions and multi-sequence alignments
	BLAST	https://blast.ncbi.nlm.nih.gov/Blast.cgi	Basic Local Alignment Search Tool which finds regions of similarity between nucleotide or amino acid sequences
	PROSITE	https://prosite.expasy.org	DB of protein domains, families and functional sites
	NCBI CDD	https://www.ncbi.nlm.nih.gov/Structure/cdd/cdd.shtml	Collection of well-annotated multiple sequence alignment models for ancient domains and full-length proteins
**Small chemical compounds**	MetaNetX	https://www.metanetx.org/	Resource unifying metabolites and biochemical reactions between 12 different resources
	HMDB	https://hmdb.ca/	DB of small metabolites found in the human body
	ChEBI	https://www.ebi.ac.uk/chebi/	Dictionary of molecular entities focused on 'small' chemical compounds
	ChemDB	http://cdb.ics.uci.edu/	Chemical DB of small molecules with information such as 3D structure, physicochemical properties, and many tools
**Taxonomy**	NCBI Taxonomy	https://www.ncbi.nlm.nih.gov/taxonomy/	Primary DB for taxonomy of any organism
	GBIF	https://www.gbif.org/	Collection of data about all types of life on Earth
	European Scorpion database	https://www.ntnu.no/ub/scorpion-files	Catalog of European scorpions
	The Reptile Database	http://reptile-database.org	Catalog of reptiles
	SnakeDB	http://snakedb.org/	Catalog of snakes and relative venom toxicity (LD(50)

	Spiders of Europe	https://araneae.nmbe.ch	Catalog of European spiders
	World Spider Catalog	https://wsc.nmbe.ch	Catalog of world spiders
	Repfocus	http://www.repfocus.dk	Catalog of reptiles
	Snakebd	http://www.snakebd.com/	Catalog of indigenous snake species of Bangladesh with respective venom composition
	Worms	https://www.marinespecies.org	Authoritative classification and catalogue of marine species
**Antimicrobial peptides**	CAMPR3	http://www.camp3.bicnirrh.res.in	Collection of anti-microbial peptides containing information related to sequence, protein definition, accession numbers, activity, source organism, target organisms and more.
	LAMP2	http://biotechlab.fudan.edu.cn/database/lamp/	Resource and tools for antimicrobial peptide studies
	DRAMP	http://dramp.cpu-bioinfor.org	Data Repository of Antimicrobial Peptides harboring annotations including patents and clinical trials and therapeutic applications
	DBAASP	https://dbaasp.org/home	Database of antimicrobial activity and structure providing information and analytical resources for designing antimicrobial compounds with a high therapeutic index
**Anticancer peptides and proteins**	CancerPPD	http://crdd.osdd.net/raghava/cancerppd/	DB of experimentally verified anticancer peptides and proteins
	canSAR	https://cansar.ai	Portal for multidisciplinary data search and AI predictions useful for drug discovery
	ApInAPDB	http://bioinf.modares.ac.ir/software/ApInAPDB/	Apoptosis-inducing anticancer peptides database
	PaccMann	https://huggingface.co/spaces/jannisborn/paccmann	Web service for anticancer compound sensitivity prediction
	Evicor	https://www.evicor.org	Web platform for exploration of molecular features and response to anti-cancer drugs
**Molecular docking**	SwissDock	http://www.swissdock.ch	Web service to predict molecular interactions a target protein and a small molecule
**Morphology**	Uberon	http://obophenotype.github.io/uberon/	Integrated cross-species anatomy ontology
	Morphobank	https://morphobank.org/	Homology of phenotypes over the web and a DB of peer-reviewed morphological matrices
**Organism-specific**	SIMRbase	https://simrbase.stowers.org	Genome Browser for various species including the sea anemone Nematostella vectensis and the worm anemone Scolanthus callimorphus
	Hydra 2.0 Web portal	https://research.nhgri.nih.gov/hydra/	Genome Browser and data search for Hydra vulgaris
	Flybase	https://flybase.org/	DB of Drosophila genes and genomes
	Human protein Atlas	https://www.proteinatlas.org/	Resource for exploration of the human proteome
	Mouse Genome Informatics	https://www.informatics.jax.org/	DB for the laboratory mouse, providing integrated genetic, genomic, and biological data
	Mousephenotype	https://www.mousephenotype.org	Resource for mouse gene function
	Rat Genome Database	https://rgd.mcw.edu/	DB for genetic, genomic, phenotype, and disease-related data generated from rat research
	Zebrafish	https://zfin.org	DB of genetic and genomic data for the zebrafish (Danio rerio) as a model organism
	MolluscDB	http://mgbase.qnlm.ac	Integrated genomics DB for Mollusca

	World Spider Trait database	https://spidertraits.sci.muni.cz	DB of morphological, ecological and other biological traits of spiders
	GeneCards	https://www.genecards.org	Human Gene DB providing comprehensive information on all annotated and predicted human genes
**Recombinant expression**	Addgene	https://www.addgene.org/	Repository of plasmids and antibodies
**Pharmacology**	IUPHAR	https://www.guidetopharmacology.org/	Resource of pharmacological targets and substances that act on them
**Resource portals**	NCBI	https://www.ncbi.nlm.nih.gov	Comprehensive resource portal for biomedical and genomic data and tools from the National Library of Medicine
	Expasy	https://www.expasy.org/	Bioinformatics resource portal of the Swiss Institute of Bioinformatics (SIB) providing access to over 160 databases and software tools for a range of life science domains.
	Galaxy	https://usegalaxy.org	Web-based platform for genomics data including hundreds of tools
	EMBL-EBI services	https://www.ebi.ac.uk/services	Molecular data resources and analysis tools maintained by the European Bioinformatics Institute (EMBL-EBI)
	Database Commons	https://ngdc.cncb.ac.cn/databasecommons/	Catalog of worldwide biological DBs
	Ontobee	https://ontobee.org	Linked data server to facilitate ontology data sharing, visualization, query, integration, and analysis
	OLS	https://www.ebi.ac.uk/ols4/	Repository for biomedical ontologies that aims to provide a single point of access to the latest ontology versions
	BioPortal	https://bioportal.bioontology.org	Repository of biomedical ontologies
**Toxicology and toxinology**	Toxbase	https://www.toxbase.org/	Subscription-based clinical toxicology DB of the UK National Poisons Information Service
	Merative Micromedex	https://www.micromedexsolutions.com/home/dispatch/ssl/true	Subscription-based resource for healthcare professionals including toxicology information
	Munich AntiVenom Index (MAVIN)	https://www.antivenoms.toxinfo.med.tum.de	List of antivenom holding centers, venomous animals, and antivenoms
	Clinical Toxinology Resources	http://www.toxinology.com	Repository of information on venomous and poisonous animals, plants and mushrooms.
	AfriTox	https://www.afritox.co.za	Subscription-based poisons information focused on Africa
	Merative Micromedex	https://www.micromedexsolutions.com/home/dispatch	Subscription-based clinical resources for healthcare professionals
	Toxinz	https://www.toxinz.com	Subscription-based resource of evidence-based information for the clinical management of poisoned patients
	WHO Snakebite	https://www.who.int/teams/control-of-neglected-tropical-diseases/snakebite-envenoming/snakebite-information-and-data-platform	WHO snakebite information and data platform
	WHO Antivenom	https://www.who.int/teams/control-of-neglected-tropical-diseases/snakebite-envenoming/antivenoms	WHO antivenom guidelines
**International organisations**	WHO	https://www.who.int	World Health Organisation with a section specific to snake bites and antivenoms

	IUCN	https://www.iucn.org/	Global authority on the status of the natural world and the measures needed to safeguard it
	WAHIS	https://wahis.oie.int	Global animal health reference DB of the World Organisation for Animal Health (WOAH)
	IST	https://www.toxinology.org	The International Society of Toxinology
	SFT	https://www.sftox.com	French Society of Toxinology
	EAPCCT	https://www.eapcct.org	European Association of Poisons Control and Clinical Toxicology
	AACT	https://www.clintox.org	American Academy of Clinical Toxicology
