# Supplementary material for: Web of venom: exploration of big data resources in animal toxin research
Source: Gigascience. 2024 Sep 9;13:giae054. doi: 10.1093/gigascience/giae054 (PMC11382406; doi:10.1093/gigascience/giae054)
Supplement: giae054_Supplemental_Files [file giae054_supplemental_files.zip › Zancolli_et_al_Supplementary_Information_revision.docx]

**Additional results**

*General overview of the venom research community in Europe: research areas, target organisms, and resources*

A survey was initiated in March 2021 and targeted the members of the European Venom Network (EUVEN) COST Action CA19144, and the participants of the 1^st^ International EUVEN Congress, a virtual event held from 14^th^ to 16^th^ September 2021. Following the congress, a total of 116 participants responded to the survey.

The landscape of venom research of the survey participants, mostly Europe-based, exhibits remarkable diversity, devoid of pronounced biases towards specific research domains (Fig. S1). Within this spectrum, researchers investigating the molecular targets of toxins and their functional implications constituted 21% of the respondents. An additional 18% were engaged in proteomic analyses to decipher venom composition, while 10% focused on toxin gene expression. Conversely, areas of more specialised focus, such as the exploration of microbial components and their translational applications in disease treatments, or anticancer investigations, remained relatively underrepresented, each accounting for only 1% of respondents.


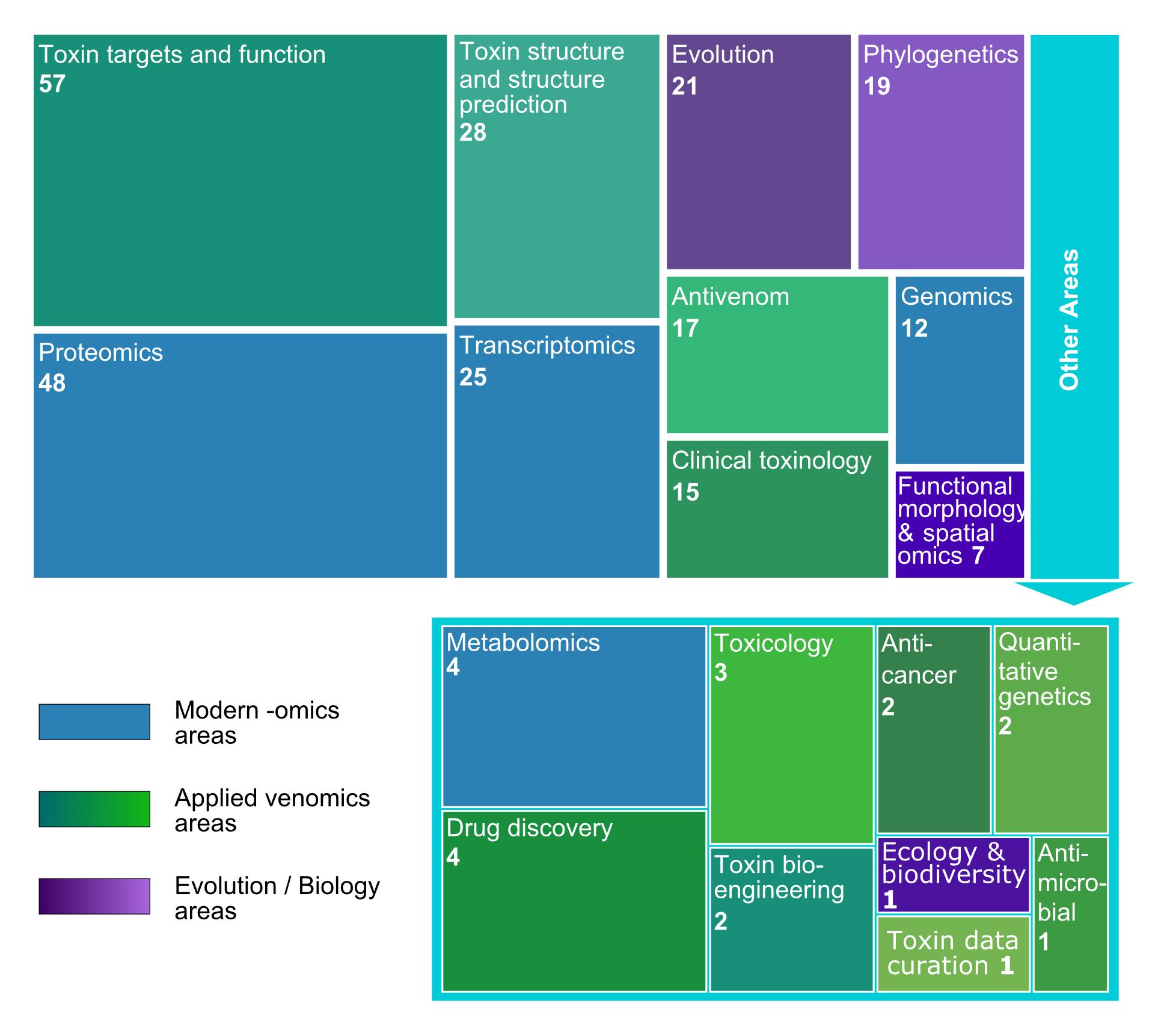


**Figure S1. Represented research domains in venom research.** The size of rectangles reflects the percentage, and the absolute numbers are indicated. The colour code indicates the major fields in venom research.

Among the animals subject to venom research, snakes were the most common (23%), followed by insects at 14%, scorpions, spiders, and cone snails at 9% (Fig. S2). A minority of participants were engaged in studying fungi, bacteria, and viruses, thereby broadening the scope of the EUVEN network beyond animal venoms.


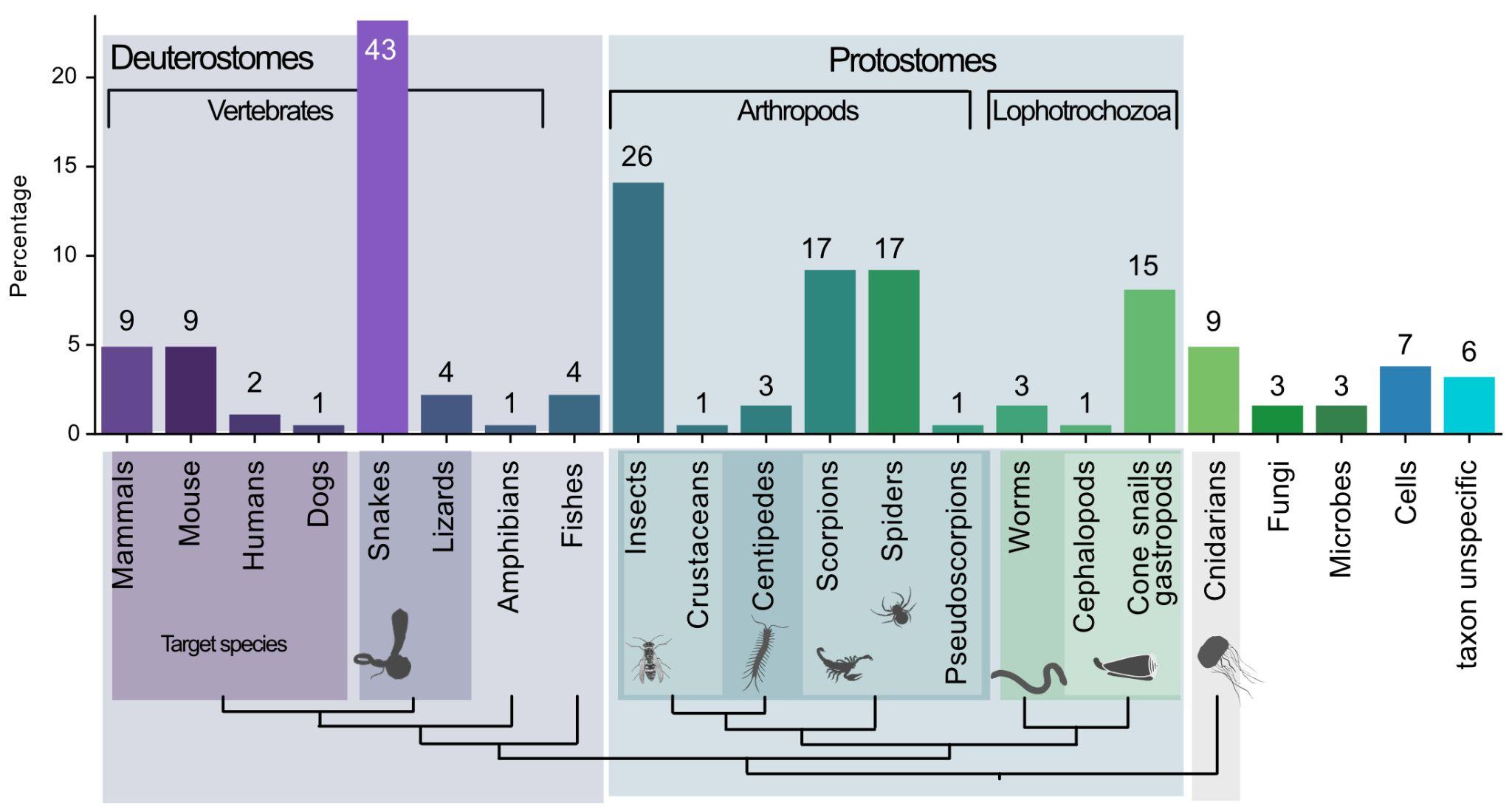


**Figure S2. Identified species that are subject of venom research.** The taxa are arranged according to the phylogeny of the animal kingdom. The mammal representatives and cells are rather utilised as target organisms to study, for instance, the effects of venom injection or toxin activity, rather than sources of venom. The bars represent the percentage among all respondents, and the absolute values are given on top.

Among the web resources used by venom researchers, protein and nucleotide sequences databases are the most common, with UniProtKB and NCBI Genbank on top of the rank with over 50% of the responders using them (Fig. S3). Among the venom-specialised resource, Tox-Prot and VenomZone were the most common, followed by ConoServer and ArachnoServer.


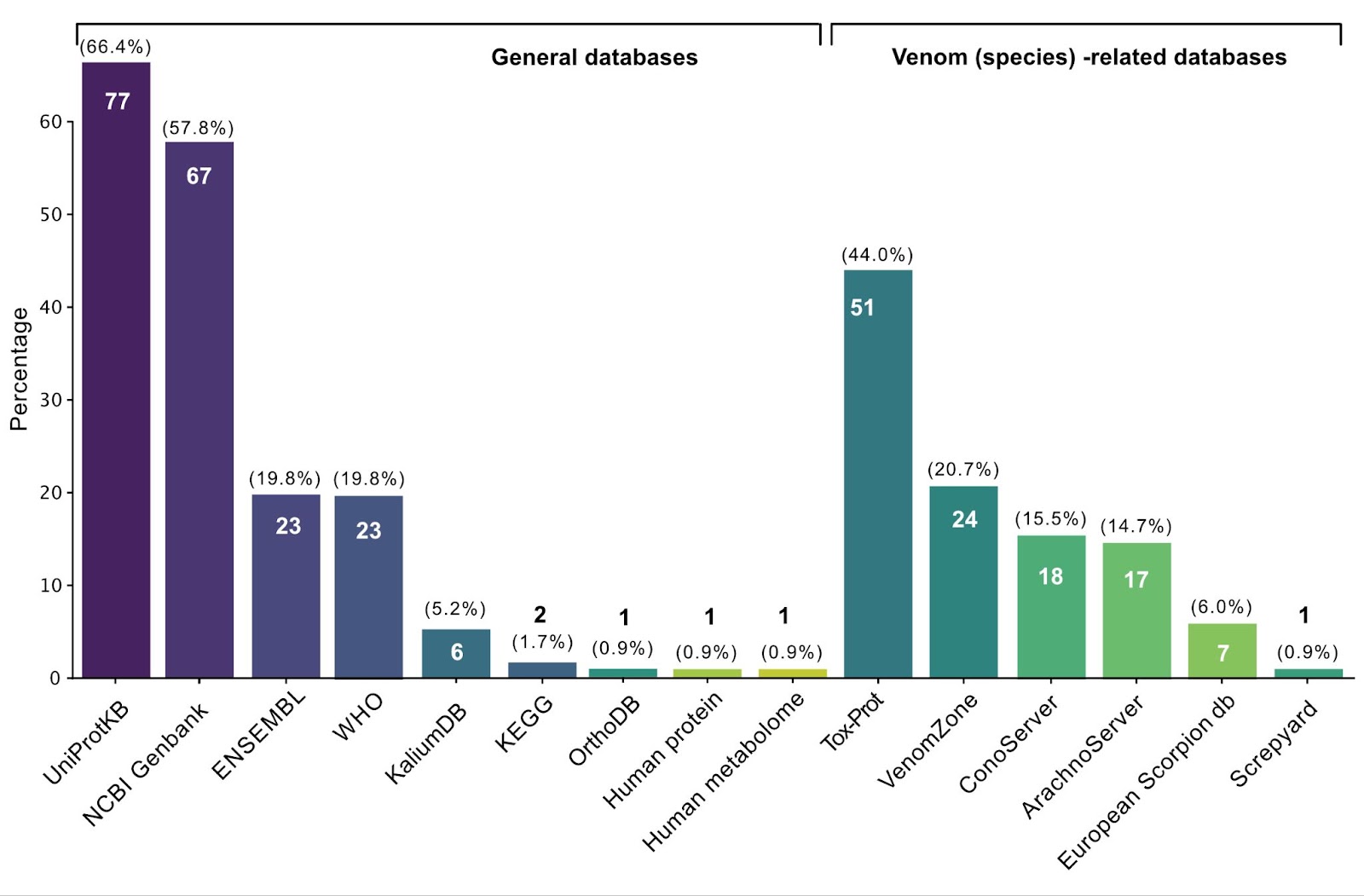


**Figure S3.** **Overview of the most widely used web resources.** On the y-axis the percentage of responders is reported per database, while the absolute numbers are given on top of the bars.


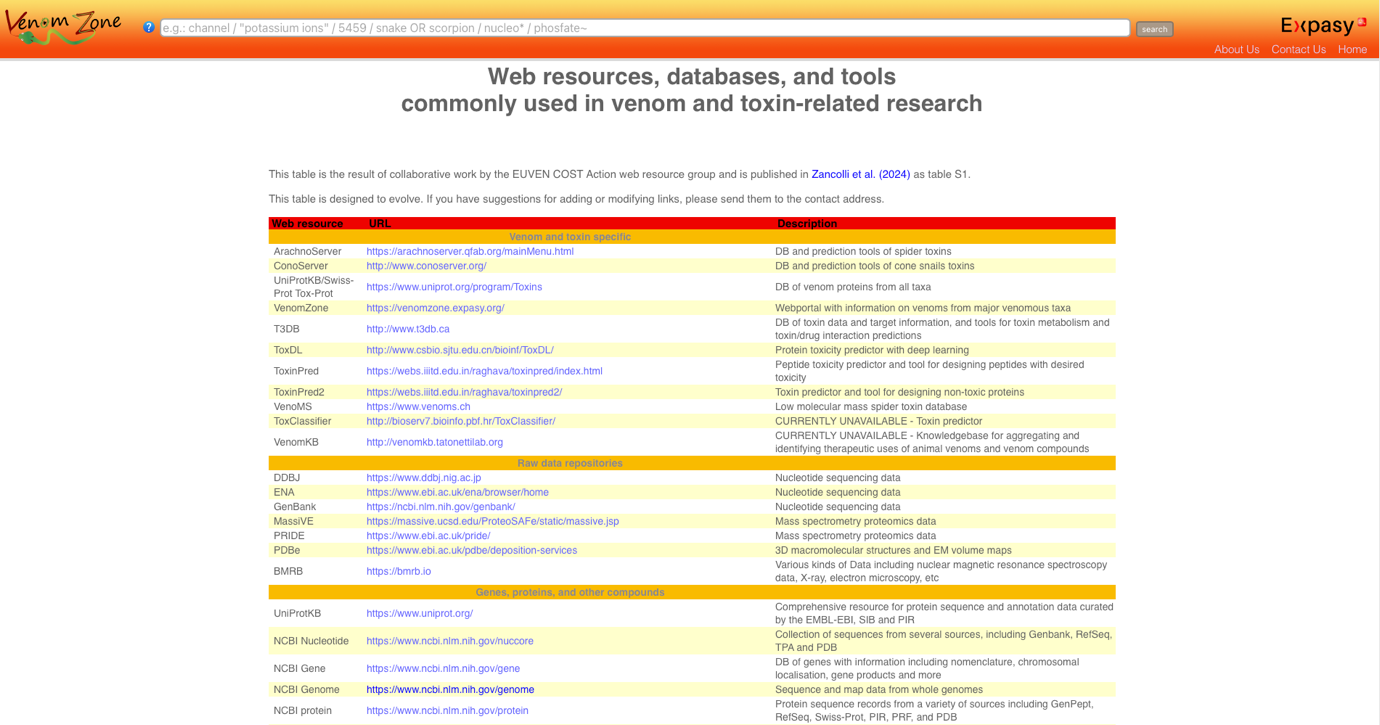


**Figure S4. Screenshot of the VenomZone webpage listing the compiled web resources utilised in venom research.** This webpage currently lists all the web resources, databases and tools gathered within the framework of this paper. New relevant databases or online tools, as well as changes in the already compiled resources, can be added by contacting the VenomZone curator using the “Contact Us” link on the top-right.

**Table S1. List of web resources and online tools utilised in venom research.**

|  | Web resource | URL | Description |
| --- | --- | --- | --- |
| Venom and toxin specific | ArachnoServer | <https://arachnoserver.qfab.org/mainMenu.html> | DB and prediction tools of spider toxins |
|  | ConoServer | <http://www.conoserver.org/> | DB and prediction tools of cone snails toxins |
|  | UniProtKB/Swiss-Prot Tox-Prot | <https://www.uniprot.org/program/Toxins> | DB of venom proteins from all taxa |
|  | VenomZone | <https://venomzone.expasy.org/> | Webportal with information on venoms from major venomous taxa |
|  | T3DB | [http://www.t3db.ca](http://www.t3db.ca/) | DB of toxin data and target information, and tools for toxin metabolism and toxin/drug interaction predictions |
|  | ToxDL | <http://www.csbio.sjtu.edu.cn/bioinf/ToxDL/> | Protein toxicity predictor with deep learning |
|  | ToxinPred | <https://webs.iiitd.edu.in/raghava/toxinpred/index.html> | Peptide toxicity predictor and tool for designing peptides with desired toxicity |
|  | ToxinPred2 | <https://webs.iiitd.edu.in/raghava/toxinpred2/> | Toxin predictor and tool for designing non-toxic proteins |
|  | VenoMS | [https://www.venoms.ch](https://www.venoms.ch/) | Low molecular mass spider toxin database |
|  | ToxClassifier | <http://bioserv7.bioinfo.pbf.hr/ToxClassifier/> | CURRENTLY UNAVAILABLE - Toxin predictor |
|  | VenomKB | [http://venomkb.tatonettilab.org](http://venomkb.tatonettilab.org/) | CURRENTLY UNAVAILABLE - Knowledgebase for aggregating and identifying therapeutic uses of animal venoms and venom compounds |
|  |  |  |  |
| Raw data repositories | DDBJ | [https://www.ddbj.nig.ac.jp](https://www.ddbj.nig.ac.jp/index-e.html) | Nucleotide sequencing data |
|  | ENA | <https://www.ebi.ac.uk/ena/browser/home> | Nucleotide sequencing data |
|  | GenBank | <https://ncbi.nlm.nih.gov/genbank/> | Nucleotide sequencing data |
|  | MassiVE | <https://massive.ucsd.edu/ProteoSAFe/static/massive.jsp> | Mass spectrometry proteomics data |
|  | PRIDE | <https://www.ebi.ac.uk/pride/> | Mass spectrometry proteomics data |
|  | PASSEL | <https://peptideatlas.org/passel/> | Selected reaction monitoring (SRM/MRM) data |
|  | PDBe | <https://www.ebi.ac.uk/pdbe/deposition-services> | 3D macromolecular structures and EM volume maps |
|  | BMRB | [https://bmrb.io](https://bmrb.io/) | Various kinds of data including nuclear magnetic resonance spectroscopy data, X-ray, electron microscopy, etc |
|  | EMDB | <https://www.ebi.ac.uk/emdb/> | Repository for cryogenic-sample Electron Microscopy (cryoEM) volumes and representative tomograms of macromolecular complexes and subcellular structures |
|  |  |  |  |
| Genes, proteins, and other compounds | UniProtKB | <https://www.uniprot.org/> | Comprehensive resource for protein sequence and annotation data curated by the EMBL-EBI, SIB and PIR |
|  | NCBI Nucleotide | <https://www.ncbi.nlm.nih.gov/nuccore> | Collection of sequences from several sources, including Genbank, RefSeq, TPA and PDB |
|  | NCBI Gene | <https://www.ncbi.nlm.nih.gov/gene> | DB of genes with information including nomenclature, chromosomal localisation, gene products and more |
|  | NCBI Genome | <https://www.ncbi.nlm.nih.gov/genome> | Sequence and map data from whole genomes |
|  | NCBI protein | <https://www.ncbi.nlm.nih.gov/protein> | Protein sequence records from a variety of sources including GenPept, RefSeq, Swiss-Prot, PIR, PRF, and PDB |
|  | NCBI PubChem | [https://pubchem.ncbi.nlm.nih.gov](https://pubchem.ncbi.nlm.nih.gov/) | DB containing chemical information on a variety of compounds |
|  | NCBI TSA | <https://www.ncbi.nlm.nih.gov/Traces/wgs/?view=TSA> | DB of computationally assembled transcript sequences from primary data such as ESTs and RNA-Seq technologies |
|  | Ensembl | <https://www.ensembl.org/> | Genome browser for vertebrate genomes with tools for comparative analysis and predictions |
|  | Ensembl Metazoa | <http://metazoa.ensembl.org/index.html> | Gene and genome data for Metazoan species |
|  | Ensembl Genome | [http://ensemblgenomes.org](http://ensemblgenomes.org/) | Genome data for non-vertebrate species with tools for manipulation, analysis and visualisation |
|  | GenomeArk | [https://www.genomeark.org](https://www.genomeark.org/) | DB of genome sequencing and assembly data generated by the Earth BioGenome Project, the Vertebrate Genomes Project, the Telomere-to-Telomere Consortium, and other related projects. |
|  | PeptideAtlas | [https://peptideatlas.org](https://peptideatlas.org/) | Multiorganism compendium of peptides identified from mass spectrometry data |
|  | Kalium DB | <https://kaliumdb.org/> | DB of polypeptidic ligands of potassium channels |
|  | ScrepYard | [https://screpyard.org](https://screpyard.org/) | DB of secreted cysteine-rich repeat peptides |
|  | APD3 | [https://aps.unmc.edu](https://aps.unmc.edu/) | Antimicrobial peptide DB |
|  |  |  |  |
| 3d structure and peptide prediction | RCSB-PDB | <https://www.rcsb.org/> | Archive of 3D structure data for large biological molecules curated by the US data center for the global protein data bank (PDB) |
|  | PDBe | <https://www.ebi.ac.uk/pdbe/> | Archive data on biological macromolecular structures curated by EMBL-EBI |
|  | BMRB | [https://bmrb.io](https://bmrb.io/) | Archive spectral and quantitative data derived from nuclear magnetic resonance |
|  | AlphaFold Protein Structure Database | <https://alphafold.ebi.ac.uk/> | DB of protein structure predictions based on an AI system developed by GoogleDeepMind |
|  | AlphaFold2 | <https://colab.research.google.com/github/sokrypton/ColabFold/blob/main/AlphaFold2.ipynb> | Server for the prediction of 3D structure form amino acid sequences |
|  | RoseTTAFold | <https://robetta.bakerlab.org/> | Protein structure and interaction prediction server including RoseTTAFold and TrRosetta |
|  | KNOTTIN | <https://www.dsimb.inserm.fr/KNOTTIN/index.php> | DB and prediction tools of small disulfide-rich proteins characterised by cysteine knots |
|  | Peptide Ranker | <http://distilldeep.ucd.ie/PeptideRanker/> | Server for the prediction of bioactive peptides based on a neural network |
|  | PEP-FOLD3 | <https://bioserv.rpbs.univ-paris-diderot.fr/services/PEP-FOLD3/> | Tool for prediction of peptide structure from amino acid sequences |
|  | PEP-FOLD4 | <http://bioserv.rpbs.univ-paris-diderot.fr/services/PEP-FOLD4> | Improved version of PEP-FOLD3 that considers pH and ionic strength variation |
|  |  |  |  |
| Gene function, interactions and networks | Gene ontology (GO) knowledgebase | <http://geneontology.org/> | Human- and machine-readable source of information on the functions of genes |
|  | STRING | <https://string-db.org/> | DB of protein associations, including both physical interactions and functional associations |
|  | KEGG | <https://www.genome.jp/kegg/> | DB for understanding high-level functions and utilities of the biological system, such as the cell, the organism and the ecosystem, from genomic and molecular-level information |
|  | IntAct | <https://www.ebi.ac.uk/intact/home> | DB and analysis tools for molecular interaction data |
|  | Bgee | [https://www.bgee.org](https://www.bgee.org/) | DB for retrieval and comparison of gene expression patterns across animal species |
|  | NCBI GEO | <https://www.ncbi.nlm.nih.gov/geo/> | Gene expression repository from array- and sequencing-based data |
|  | Pannzer2 | <http://ekhidna2.biocenter.helsinki.fi/sanspanz/> | Fully automated service for functional annotation of proteins of unknown function |
|  | Philius | <https://www.yeastrc.org/philius/pages/philius/runPhilius.jsp> | Transmembrane prediction server |
|  | SignalP | <https://services.healthtech.dtu.dk/services/SignalP-6.0/> | Prediction of signal peptides and their cleavage sites from amino acid sequences |
|  | Phobius | [https://phobius.sbc.su.se](https://phobius.sbc.su.se/) | Combined transmembrane topology and signal peptide predictor tool |
|  | WoLF PSORT | [https://wolfpsort.hgc.jp](https://wolfpsort.hgc.jp/) | Protein subcellular localisation prediction |
|  | The Eukaryotic Linear Motif resource for | <http://elm.eu.org/> | Resource for annotation and detection of Eukaryotic Linear Motifs (ELM) or Short Linear Motifs (SLiMs) of adjacent amino acids |
|  | Rhea - reaction knowledgebase | [https://www.rhea-db.org](https://www.rhea-db.org/) | DB for chemical and transport reactions |
|  | WashU Epigenome Browser | <https://epigenomegateway.wustl.edu/browser/> | Genomics data exploration tool for the visualisation, integration adn analysis of epigenomic datasets |
|  |  |  |  |
| Gene families and classification | Pfam | <http://pfam.xfam.org/> | Large collection of protein families, each represented by multiple sequence alignments and hidden Markov models (HMMs) |
|  | Interpro | <http://www.ebi.ac.uk/interpro/> | Resource providing functional analysis of proteins by classifying them into families and predicting domains and important sites |
|  | PANTHER | [https://www.pantherdb.org](https://www.pantherdb.org/) | Comprehensive information about the evolution of protein-coding gene families |
|  | Quest for Orthologs | <https://questfororthologs.org/orthology_databases> | List of orthology databases |
|  | OrthoDB | <https://www.orthodb.org/> | Hierarchical catalog of orthologs - mapping genomics to functional data |
|  | OMA | <https://omabrowser.org/oma/home/> | DB and method for the inference of orthologs among complete genomes |
|  | NCBI COG | <https://www.ncbi.nlm.nih.gov/research/cog> | DB of clusters of orthologous genes |
|  | PhylomeDB | [http://phylomedb.org](http://phylomedb.org/) | DB of catalogues of gene phylogenies with phylogenetic trees, orthology predictions and multi-sequence alignments |
|  | BLAST | <https://blast.ncbi.nlm.nih.gov/Blast.cgi> | Basic Local Alignment Search Tool which finds regions of similarity between nucleotide or amino acid sequences |
|  | PROSITE | [https://prosite.expasy.org](https://prosite.expasy.org/) | DB of protein domains, families and functional sites |
|  | NCBI CDD | <https://www.ncbi.nlm.nih.gov/Structure/cdd/cdd.shtml> | Collection of well-annotated multiple sequence alignment models for ancient domains and full-length proteins |
|  |  |  |  |
| Small chemical compounds | MetaNetX | <https://www.metanetx.org/> | Resource unifying metabolites and biochemical reactions between 12 different resources |
|  | HMDB | <https://hmdb.ca/> | DB of small metabolites found in the human body |
|  | ChEBI | <https://www.ebi.ac.uk/chebi/> | Dictionary of molecular entities focused on ‘small’ chemical compounds |
|  | ChemDB | <http://cdb.ics.uci.edu/> | Chemical DB of small molecules with information such as 3D structure, physicochemical properties, and many tools |
|  |  |  |  |
| Taxonomy | NCBI Taxonomy | <https://www.ncbi.nlm.nih.gov/taxonomy/> | Primary DB for taxonomy of any organism |
|  | GBIF | <https://www.gbif.org/> | Collection of data about all types of life on Earth |
|  | European Scorpion database | <https://www.ntnu.no/ub/scorpion-files> | Catalog of European scorpions |
|  | The Reptile Database | [http://reptile-database.org](http://reptile-database.org/) | Catalog of reptiles |
|  | SnakeDB | <http://snakedb.org/> | Catalog of snakes and relative venom toxicity (LD(50) |
|  | Spiders of Europe | [https://araneae.nmbe.ch](https://araneae.nmbe.ch/) | Catalog of European spiders |
|  | World Spider Catalog | [https://wsc.nmbe.ch](https://wsc.nmbe.ch/) | Catalog of world spiders |
|  | Repfocus | [http://www.repfocus.dk](http://www.repfocus.dk/) | Catalog of reptiles |
|  | Snakebd | <http://www.snakebd.com/> | Catalog of indigenous snake species of Bangladesh with respective venom composition |
|  | Worms | [https://www.marinespecies.org](https://www.marinespecies.org/) | Authoritative classification and catalogue of marine species |
|  |  |  |  |
| Antimicrobial peptides | CAMPR3 | [http://www.camp3.bicnirrh.res.in](http://www.camp3.bicnirrh.res.in/) | Collection of anti-microbial peptides containing information related to sequence, protein definition, accession numbers, activity, source organism, target organisms and more. |
|  | LAMP2 | <http://biotechlab.fudan.edu.cn/database/lamp/> | Resource and tools for antimicrobial peptide studies |
|  | DRAMP | [http://dramp.cpu-bioinfor.org](http://dramp.cpu-bioinfor.org/) | Data Repository of Antimicrobial Peptides harboring annotations including patents and clinical trials and therapeutic applications |
|  | DBAASP | <https://dbaasp.org/home> | Database of antimicrobial activity and structure providing information and analytical resources for designing antimicrobial compounds with a high therapeutic index |
|  |  |  |  |
| Anticancer peptides and proteins | CancerPPD | <http://crdd.osdd.net/raghava/cancerppd/> | DB of experimentally verified anticancer peptides and proteins |
|  | canSAR | [https://cansar.ai](https://cansar.ai/) | Portal for multidisciplinary data search and AI predictions useful for drug discovery |
|  | ApInAPDB | <http://bioinf.modares.ac.ir/software/ApInAPDB/> | Apoptosis-inducing anticancer peptides database |
|  | PaccMann | <https://huggingface.co/spaces/jannisborn/paccmann> | Web service for anticancer compound sensitivity prediction |
|  | Evicor | [https://www.evicor.org](https://www.evicor.org/) | Web platform for exploration of molecular features and response to anti-cancer drugs |
|  |  |  |  |
| Molecular docking | SwissDock | [http://www.swissdock.ch](http://www.swissdock.ch/) | Web service to predict molecular interactions a target protein and a small molecule |
|  |  |  |  |
| Morphology | Uberon | [http://obophenotype.github.io/uberon/](https://www.google.com/url?q=http://obophenotype.github.io/uberon/&sa=D&source=editors&ust=1710845703500320&usg=AOvVaw0Dbiw1E-6EwTH4n4vktyzM) | Integrated cross-species anatomy ontology |
|  | Morphobank | <https://morphobank.org/> | Homology of phenotypes over the web and a DB of peer-reviewed morphological matrices |
|  |  |  |  |
| Organism-specific | SIMRbase | [https://simrbase.stowers.org](https://simrbase.stowers.org/) | Genome Browser for various species including the sea anemone Nematostella vectensis and the worm anemone Scolanthus callimorphus |
|  | Hydra 2.0 Web portal | <https://research.nhgri.nih.gov/hydra/> | Genome Browser and data search for Hydra vulgaris |
|  | Flybase | <https://flybase.org/> | DB of Drosophila genes and genomes |
|  | Human protein Atlas | <https://www.proteinatlas.org/> | Resource for exploration of the human proteome |
|  | Mouse Genome Informatics | [https://www.informatics.jax.org/](https://www.google.com/url?q=https://www.informatics.jax.org/&sa=D&source=editors&ust=1712311497019836&usg=AOvVaw2gwqzTYbrZccAydCeecAeO) | DB for the laboratory mouse, providing integrated genetic, genomic, and biological data |
|  | Mousephenotype | [https://www.mousephenotype.org](https://www.mousephenotype.org/) | Resource for mouse gene function |
|  | Rat Genome Database | <https://rgd.mcw.edu/> | DB for genetic, genomic, phenotype, and disease-related data generated from rat research |
|  | Zebrafish | [https://zfin.org](https://zfin.org/) | DB of genetic and genomic data for the zebrafish (Danio rerio) as a model organism |
|  | MolluscDB | [http://mgbase.qnlm.ac](http://mgbase.qnlm.ac/) | Integrated genomics DB for Mollusca |
|  | World Spider Trait database | [https://spidertraits.sci.muni.cz](https://spidertraits.sci.muni.cz/) | DB of morphological, ecological and other biological traits of spiders |
|  | GeneCards | [https://www.genecards.org](https://www.genecards.org/) | Human Gene DB providing comprehensive information on all annotated and predicted human genes |
|  |  |  |  |
| Recombinant expression | Addgene | <https://www.addgene.org/> | Repository of plasmids and antibodies |
|  |  |  |  |
| Pharmacology | IUPHAR | <https://www.guidetopharmacology.org/> | Resource of pharmacological targets and substances that act on them |
|  |  |  |  |
| Resource portals | NCBI | [https://www.ncbi.nlm.nih.gov](https://www.ncbi.nlm.nih.gov/) | Comprehensive resource portal for biomedical and genomic data and tools from the National Library of Medicine |
|  | Expasy | <https://www.expasy.org/> | Bioinformatics resource portal of the Swiss Institute of Bioinformatics (SIB) providing access to over 160 databases and software tools for a range of life science domains. |
|  | Galaxy | [https://usegalaxy.org](https://usegalaxy.org/) | Web-based platform for genomics data including hundreds of tools |
|  | EMBL-EBI services | <https://www.ebi.ac.uk/services> | Molecular data resources and analysis tools maintained by the European Bioinformatics Institute (EMBL-EBI) |
|  | Database Commons | <https://ngdc.cncb.ac.cn/databasecommons/> | Catalog of worldwide biological DBs |
|  | Ontobee | [https://ontobee.org](https://ontobee.org/) | Linked data server to facilitate ontology data sharing, visualization, query, integration, and analysis |
|  | OLS | <https://www.ebi.ac.uk/ols4/> | Repository for biomedical ontologies that aims to provide a single point of access to the latest ontology versions |
|  | BioPortal | [https://bioportal.bioontology.org](https://bioportal.bioontology.org/) | Repository of biomedical ontologies |
|  |  |  |  |
| Toxicology and toxinology | Toxbase | <https://www.toxbase.org/> | Subscription-based clinical toxicology DB of the UK National Poisons Information Service |
|  | Merative Micromedex | <https://www.micromedexsolutions.com/home/dispatch/ssl/true> | Subscription-based resource for healthcare professionals including toxicology information |
|  | Munich AntiVenom Index (MAVIN) | [https://www.antivenoms.toxinfo.med.tum.de](https://www.antivenoms.toxinfo.med.tum.de/) | List of antivenom holding centers, venomous animals, and antivenoms |
|  | Clinical Toxinology Resources | [http://www.toxinology.com](http://www.toxinology.com/) | Repository of information on venomous and poisonous animals, plants and mushrooms. |
|  | AfriTox | [https://www.afritox.co.za](https://www.afritox.co.za/) | Subscription-based poisons information focused on Africa |
|  | Merative Micromedex | <https://www.micromedexsolutions.com/home/dispatch> | Subscription-based clinical resources for healthcare professionals |
|  | Toxinz | [https://www.toxinz.com](https://www.toxinz.com/) | Subscription-based resource of evidence-based information for the clinical management of poisoned patients |
|  | WHO Snakebite | <https://www.who.int/teams/control-of-neglected-tropical-diseases/snakebite-envenoming/snakebite-information-and-data-platform> | WHO snakebite information and data platform |
|  | WHO Antivenom | <https://www.who.int/teams/control-of-neglected-tropical-diseases/snakebite-envenoming/antivenoms> | WHO antivenom guidelines |
|  |  |  |  |
| International organisations | WHO | [https://www.who.int](https://www.who.int/) | World Health Organisation with a section specific to snake bites and antivenoms |
|  | IUCN | <https://www.iucn.org/> | Global authority on the status of the natural world and the measures needed to safeguard it |
|  | WAHIS | [https://wahis.oie.int](https://wahis.oie.int/) | Global animal health reference DB of the World Organisation for Animal Health (WOAH) |
|  | IST | [https://www.toxinology.org](https://www.toxinology.org/) | The International Society of Toxinology |
|  | SFT | [https://www.sftox.com](https://www.sftox.com/) | French Society of Toxinology |
|  | EAPCCT | [https://www.eapcct.org](https://www.eapcct.org/) | European Association of Poisons Control and Clinical Toxicology |
|  | AACT | [https://www.clintox.org](https://www.clintox.org/) | American Academy of Clinical Toxicology |
