# Supplementary material for: Web of venom: exploration of big data resources in animal toxin research
Source: Gigascience. 2024 Sep 9;13:giae054. doi: 10.1093/gigascience/giae054 (PMC11382406; doi:10.1093/gigascience/giae054)
Supplement: giae054_Response_to_Reviewer_Comments_Original_Submission [file giae054_response_to_reviewer_comments_original_submission.pdf]

Author's Response To Reviewer Comments

Dear Dr Hongling Zhou,

Thank you for taking the time to review our manuscript. We have throughly read the reviewer's feedback and revised our manuscript accordingly.
In addition to addressing the reviewers' comments, we updated the reference [86] (Almeida et al. 2020) with the following more recent publication:

86. Agüero-Chapin G, Domínguez-Pérez D, Marrero-Ponce Y, Castillo-Mendieta K, Antunes A. 2024. Unveiling Encrypted Antimicrobial Peptides from Cephalopods' Salivary Glands: A Proteolysis-Driven Virtual Approach. ACS Omega https://doi.org/10.1021/acsomega.4c01959

Below we provide point-by-point answers to the comments.

Reviewer reports:
Reviewer #1: The authors established a website of the VenomZone with the main aim to provide a combined web resource of venom and toxin-related platforms. It may be helpful for convenient exploration of big data in animal toxin research. This manuscript can become acceptable after the authors make minor revisions. For example, concerning the VenomZone, the authors should offer a screenshot of the webpage (as a figure), and provide more explanations of this website (such as its structure and how to fulfil some special functions).

Thank you for your feedback. We would like to clarify that VenomZone was not built in this paper, but is an existing website developed many years ago as part of the Expasy suite. In this paper, we added a page on the website correspinding to Table S1. However, considering that there is no existing paper describing VenomZone, we have now added a more comprehensive description of the website's structure (see lines 215 – 220).
Additionally, we have added a screenshot of the webpage as Supplementary Materials Figure S4, and cited in the main text at line 700.

Reviewer #2: This article explores the significant hurdles and promising opportunities presented by the broad and encompassing field of venom research using heterogeneous data types. The authors provide a comprehensive review of existing databases and tools integral to various aspects of venom research, such as genomics, transcriptomics, proteomics, metabolomics, and translational research.

The manuscript highlights the challenge of navigating the dispersed nature of venom and toxin data, emphasizing the need for standardized terminologies and classification systems across different taxa, databases, and research pipelines. Although this is a perpetual problem, the manuscript is a first step towards concatenating the tools to make tackling this issue possible. The pitfalls and triumphs of the current infrastructure are outlined manuscript underscores the importance of various initiatives and organizations in place to make this possible. The article also emphasizes the importance of data and method sharing, advocating for greater transparency and accessibility to propel venom research forward.

The manuscript concludes by proposing the creation of a unified, venom-specific web resource, suggesting the existing VenomZone platform as a potential foundation. This comprehensive database would consolidate information on venomous species, venom composition, toxin structures and activities, and their potential applications in medicine and other fields. The authors acknowledge the substantial resources and international collaboration required for such an undertaking. They propose their interactive table, available on the VenomZone website, as a step towards achieving this goal.

One limitation of the manuscript, and potentially this endeavor overall, is that it does not clearly outline the limitations in keeping VenomZone updated, curated, and operational. The authors reference several resources that are outdated or require maintenance, highlighting this as a drawback of current approaches. Therefore, it seems essential to have a plan in place for the curation and upkeep of such an ambitious

project. New data, databases, pipelines, and resources will undoubtedly emerge following the publication of this manuscript. Including a detailed plan for supporting the longevity and continued updates of VenomZone would enhance the manuscript significantly. Although this is a minor suggestion, it is crucial for ensuring the long-term value of this important resource.

Thank you for the comprehensive summary of our paper and your constructive feedback. Indeed, keeping web resources and databases alive is a fundamental issue in the era of big data. In this revised version of the manuscript, we have acknowledged this aspect, and discussed it at lines 601-606.

Minor Comments Manuscript
• Line 182: PDB abbreviation used (and later on line 398), but RCSB PDB abbreviation used in line 180, suggest switching abbreviation in line 180 to simply PDB instead of RCSB PDB.
Done.
• Line 212: UniProtKB/Tox-Prot is referenced here, but earlier (line 170) is UniProtKB/Swiss- Prot is referenced as host to Tox-Prot. I think Tox-Prot would be relevant for the reference here instead of UniProtKB/Tox-Prot.
Corrected to only Tox-Prot at line 212 and 217 as suggested.
• Line 236: Include the citation for STAR (https://doi.org/10.1093%2Fbioinformatics%2Fbts635)
Done, thank you.
• Line 270 – 275: This sentence seems long and a bit convoluted, I'd suggest splitting this up, or perhaps just replace unravel with unraveling (line 273)
We have shorten this sentence and replaced unravel with unravelling (lines 273-277).
• Line 276: Replace "known," functionally characterized would be better.
Done (line 278).
• Line 284: Rephrase, as written "neglects" seems odd.
Changed 'neglets' with 'overlooks'.
• Line 299: Rephrase to "... most widely employed ..."
Done.
• Line 342 – 344: Are there examples of this that would help reinforce this statement?
Yes, of course. We have now added some references as examples (ref. 98-102).
• Line 377: Why is it pertinent to specify it's freely available? If others are not this could be emphasized as a limitation for broad adoption for venom research.
Indeed. We have now removed it.
• Line 503 – 504: Rephrase, I presume scientists working in most areas are interested in a variety of information.
We have now linked this sentence to the next one, making this statement more precise (see lines 504-507).
• Line 590: The use of thesauri here is confusing, not sure what the authors are intending here.
We have changed "thesauri" with "glossaries" which is more generic and understandable.
• "Contact Us" link on the VenomZone website doesn't work
Thank you for pointing this out. We have now fixed the issue.
