# Supplementary material for: Web of venom: exploration of big data resources in animal toxin research
Source: Gigascience. 2024 Sep 9;13:giae054. doi: 10.1093/gigascience/giae054 (PMC11382406; doi:10.1093/gigascience/giae054)
Supplement: giae054_Reviewer_2_Report_Original_Submission — Jason Macrander, Ph. D. -- 6/22/2024 Reviewed [file giae054_reviewer_2_report_original_submission.pdf]

Reviewer Report

Title: Web of venom: exploration of big data resources in animal toxin research

Version: Original Submission **Date:** 6/22/2024

Reviewer name: Jason Macrander, Ph. D.

Reviewer Comments to Author:

This article explores the significant hurdles and promising opportunities presented by the broad and encompassing field of venom research using heterogeneous data types. The authors provide a comprehensive review of existing databases and tools integral to various aspects of venom research, such as genomics, transcriptomics, proteomics, metabolomics, and translational research.

The manuscript highlights the challenge of navigating the dispersed nature of venom and toxin data, emphasizing the need for standardized terminologies and classification systems across different taxa, databases, and research pipelines. Although this is a perpetual problem, the manuscript is a first step towards concatenating the tools to make tackling this issue possible. The pitfalls and triumphs of the current infrastructure are outlined manuscript underscores the importance of various initiatives and organizations in place to make this possible. The article also emphasizes the importance of data and method sharing, advocating for greater transparency and accessibility to propel venom research forward.

The manuscript concludes by proposing the creation of a unified, venom-specific web resource, suggesting the existing VenomZone platform as a potential foundation. This comprehensive database would consolidate information on venomous species, venom composition, toxin structures and activities, and their potential applications in medicine and other fields. The authors acknowledge the substantial resources and international collaboration required for such an undertaking. They propose their interactive table, available on the VenomZone website, as a step towards achieving this goal.

One limitation of the manuscript, and potentially this endeavor overall, is that it does not clearly outline the limitations in keeping VenomZone updated, curated, and operational. The authors reference several resources that are outdated or require maintenance, highlighting this as a drawback of current approaches. Therefore, it seems essential to have a plan in place for the curation and upkeep of such an ambitious project. New data, databases, pipelines, and resources will undoubtedly emerge following the publication of this manuscript. Including a detailed plan for supporting the longevity and continued updates of VenomZone would enhance the manuscript significantly. Although this is a minor suggestion, it is crucial for ensuring the long-term value of this important resource.

Minor comments are in the attached pdf.

Methods

Are the methods appropriate to the aims of the study, are they well described, and are necessary controls included? Choose an item.

Conclusions

Are the conclusions adequately supported by the data shown? Choose an item.

Reporting Standards

Does the manuscript adhere to the journal's guidelines on minimum standards of reporting? Choose an item.

Choose an item.

Statistics

Are you able to assess all statistics in the manuscript, including the appropriateness of statistical tests used? Choose an item.

Quality of Written English

Please indicate the quality of language in the manuscript: Choose an item.

Declaration of Competing Interests

Please complete a declaration of competing interests, considering the following questions:

- Have you in the past five years received reimbursements, fees, funding, or salary from an organisation that may in any way gain or lose financially from the publication of this manuscript, either now or in the future?
- Do you hold any stocks or shares in an organisation that may in any way gain or lose financially from the publication of this manuscript, either now or in the future?
- Do you hold or are you currently applying for any patents relating to the content of the manuscript?
- Have you received reimbursements, fees, funding, or salary from an organization that holds or has applied for patents relating to the content of the manuscript?
- Do you have any other financial competing interests?
- Do you have any non-financial competing interests in relation to this paper?

If you can answer no to all of the above, write 'I declare that I have no competing interests' below. If your reply is yes to any, please give details below.

I declare that I have no competing interests.

I agree to the open peer review policy of the journal. I understand that my name will be included on my report to the authors and, if the manuscript is accepted for publication, my named report including any attachments I upload will be posted on the website along with the authors' responses. I agree for my

report to be made available under an Open Access Creative Commons CC-BY license (http://creativecommons.org/licenses/by/4.0/). I understand that any comments which I do not wish to be included in my named report can be included as confidential comments to the editors, which will not be published.

Choose an item.

To further support our reviewers, we have joined with Publons, where you can gain additional credit to further highlight your hard work (see: https://publons.com/journal/530/gigascience). On publication of this paper, your review will be automatically added to Publons, you can then choose whether or not to claim your Publons credit. I understand this statement.

Yes Choose an item.
